# Unveiling the Potential of *Ent*-Kaurane Diterpenoids: Multifaceted Natural Products for Drug Discovery

**DOI:** 10.3390/ph17040510

**Published:** 2024-04-16

**Authors:** Shadrack Kibet, Njogu M. Kimani, Syombua S. Mwanza, Cynthia M. Mudalungu, Cleydson B. R. Santos, Chrysantus M. Tanga

**Affiliations:** 1 Department of Physical Sciences, University of Embu, Embu P.O. Box 6-60100, Kenya; skibet@icipe.org (S.K.); smwanza@icipe.org (S.S.M.); 2 International Centre of Insects Physiology and Ecology, Nairobi P.O. Box 30772-00100, Kenya; cmudalungu@icipe.org; 3 Natural Product Chemistry and Computational Drug Discovery Laboratory, Embu P.O. Box 6-60100, Kenya; 4 School of Chemistry and Material Science, The Technical University of Kenya, Nairobi P.O. Box 52428-00200, Kenya; 5 Graduate Program in Medicinal Chemistry and Molecular Modelling, Health Science Institute, Federal University of Pará, Belém 66075-110, Brazil; breno@unifap.br; 6 Laboratory of Modelling and Computational Chemistry, Department of Biological and Health Sciences, Federal University of Amapá, Macapá 68902-280, Brazil

**Keywords:** *ent*-kauranes, natural products, diterpenoids, ADMET properties, drug-likeness, pharmacokinetics

## Abstract

Natural products hold immense potential for drug discovery, yet many remain unexplored in vast libraries and databases. In an attempt to fill this gap and meet the growing demand for effective drugs, this study delves into the promising world of *ent*-kaurane diterpenoids, a class of natural products with huge therapeutic potential. With a dataset of 570 *ent*-kaurane diterpenoids obtained from the literature, we conducted an in silico analysis, evaluating their physicochemical, pharmacokinetic, and toxicological properties with a focus on their therapeutic implications. Notably, these natural compounds exhibit drug-like properties, aligning closely with those of FDA-approved drugs, indicating a high potential for drug development. The ranges of the physicochemical parameters were as follows: molecular weights—288.47 to 626.82 g/mol; number of heavy atoms—21 to 44; the number of hydrogen bond donors and acceptors—0 to 8 and 1 to 11, respectively; the number of rotatable bonds—0 to 11; fraction Csp3—0.65 to 1; and TPSA—20.23 to 189.53 Å². Additionally, the majority of these molecules display favorable safety profiles, with only 0.70%, 1.40%, 0.70%, and 46.49% exhibiting mutagenic, tumorigenic, reproduction-enhancing, and irritant properties, respectively. Importantly, *ent*-kaurane diterpenoids exhibit promising biopharmaceutical properties. Their average lipophilicity is optimal for drug absorption, while over 99% are water-soluble, facilitating delivery. Further, 96.5% and 28.20% of these molecules exhibited intestinal and brain bioavailability, expanding their therapeutic reach. The predicted pharmacological activities of these compounds encompass a diverse range, including anticancer, immunosuppressant, chemoprotective, anti-hepatic, hepatoprotectant, anti-inflammation, antihyperthyroidism, and anti-hepatitis activities. This multi-targeted profile highlights *ent*-kaurane diterpenoids as highly promising candidates for further drug discovery endeavors.

## 1. Introduction

Natural products have for a long time been used as nutraceuticals [1,2,3]. The search for natural products continues to grow due to their unique and vast chemical and biological properties. These properties have been subjected to natural selection and evolutionary processes, shaping their utility over hundreds of thousands of years. Consequently, they display a great variety of biological and therapeutic potentials spanning anti-tumor, antimicrobial, antiviral, antitripanosomal, α-Glucosidase inhibitory activity, and immunosuppressive actions to the inhibition of vascular smooth muscle contraction [4,5,6,7,8,9,10,11,12]. More than one-third of the drugs so far approved by the United States’ Food and Drug Administration (FDA) are either derived from natural product precursors or are purely natural products [13,14,15].

Many of the natural products with therapeutic potential have been isolated from various sources. Nonetheless, their progression towards clinical utilization has often been met by significant impediments, attributable to multifaceted obstacles. These challenges include stringent regulatory guidelines governing experimental tests, especially when dealing with human subjects [16]. The conventional process of drug development is also laborious and financially stretched. For instance, a single drug requires 8–12 years of research and approximately USD 2.7 billion to be developed [17]. However, there is a very high attrition and failure rate (80–90%) of potential drugs in drug discovery pipelines, leading to huge financial losses [18]. To circumvent these, several in silico approaches have been brought upfront to determine the drug-likeness of a molecule. These approaches have linked the reduced likelihood of attrition of a potential drug to the control of physicochemical and ADMET properties [19,20,21,22,23,24,25].

In the existing libraries of natural products, there is proof that resources and labor-intensive approaches have been employed to isolate and characterize bioactive compounds [26]. Some of these compounds possess several bioactivities [27,28,29] and yet are relegated to natural product databases because of the high possibility of their failures if pursued in the classical drug discovery pipelines. Previous in silico studies on compounds isolated from diverse genera have shown that these compounds have great potential but are underexplored [30,31,32]. One particular class of vastly bioactive set of compounds that have been isolated from a diverse source is the *ent*-kaurane diterpenoids.

*Ent*-kaurane diterpenoids are an important class of natural products derived majorly from *Jungermania* species of liverworts and higher plants of the genus Isodon [5,6,7,33,34] in addition to other minor sources such as corn silk [35] and genus Chelonopsis [9]. Structurally, these molecules are constituted by a perhydrophenanthrene subunit (rings A, B and C) and a cyclopentane, ring D. *Ent*-kauranes differ from kauranes by the inversion of carbon configurations at C-5, C-9, and C-10 [36]. 

Their history dates back to the year 1961 after the discovery of *ent*-kaurene from the leaf essential oil of the New Zealand kauri [5]. These biomolecules have been reported to cause cell cycle arrest and apoptosis while exhibiting moderate-to-low toxicities on normal cell lines. Therefore, they have been the center of focus in the continuous search for active biomolecules. The objective of this study was to review the existing literature on *ent*-kaurane diterpenoids with a focus on phytochemistry. Further, the study involves an extensive investigation using in silico approaches to evaluate the compounds’ drug-like properties, with specificity on absorptivity, distribution, metabolism, excretion, and toxicity (ADMET). Additionally, bioactivity scores, biological potentials, and macromolecular targets of a selected subset are determined in silico.

## 2. Results

### 2.1. Bioactivity of Ent-Kaurane Diterpenoids

**Figure 1:**
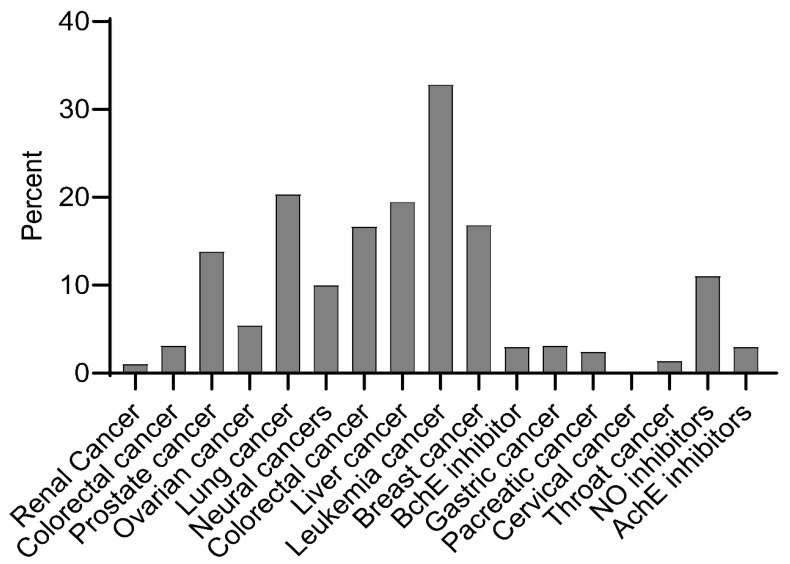
A bar graph showing the percentage of molecules exhibiting various bioactivities.

This review utilized a dataset of 570 *ent*-kaurane diterpenoids accessed from the literature (Supplementary Information S1). These molecules have diverse bioactivities including anticancer and the inhibition of NO production (Figure 1, Supplementary Information S2 and S3). Figure 1 presents the number of molecules demonstrating different bioactivities, expressed as a percentage of the total molecules (*n* = 570).

### 2.2. Physicochemical Properties of Ent-Kaurane Diterpenoids

**Figure 2:**
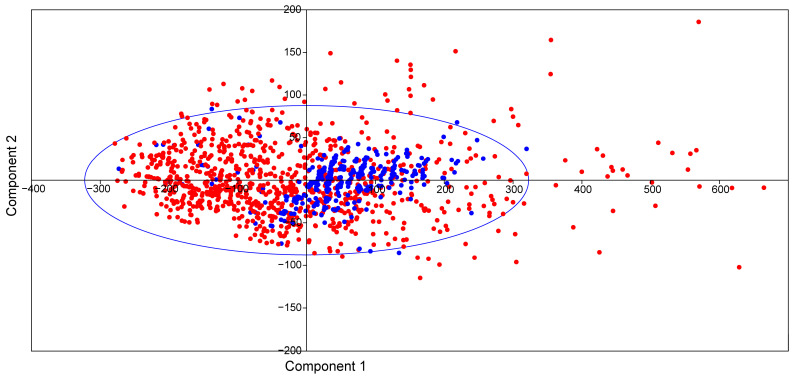
The scatter (PCA) representation of the physicochemical parameters of *ent*-kaurane diterpenoids (blue-colored data points) in comparison to the FDA-approved drugs (red-colored data points).

The physicochemical properties were similar to those of FDA-approved drugs, as shown by the scatter plot in Figure 2. The considered properties included the molecular weights, the number of heavy atoms, the number of aromatic heavy atoms, fraction Csp3, the number of rotatable bonds, the number of hydrogen bond acceptors and donors, topological polar surface (TPSA), and molar refractivity (MR). Based on this initial screening, the distribution of the physicochemical parameters of the *ent*-kaurane diterpenoids (blue colored data points) was within the chemical spaces of the FDA-approved drugs (red colored data points).

**Figure 3:**
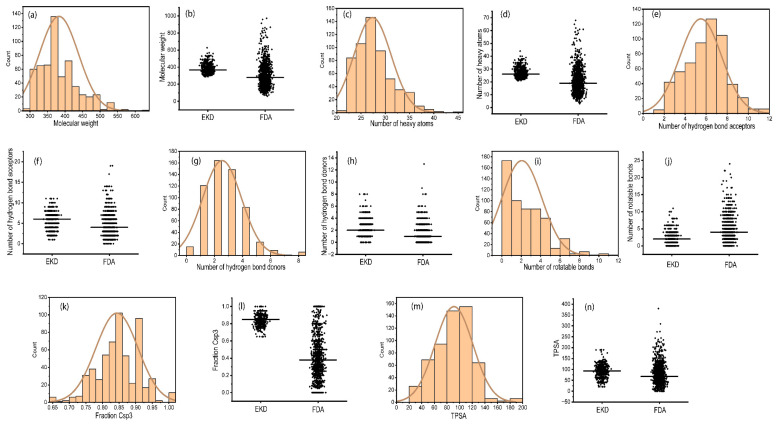
(**a**,**c**,**e**,**g**,**i**,**k**,**m**) Distribution plots showing a characteristic distribution of various physicochemical properties of sampled *ent*-kaurane diterpenoids. (**b**,**d**,**f**,**h**,**j**,**l**,**n**) Data distribution plots of various physicochemical properties of *ent*-kaurane diterpenoids in comparison to those of FDA-approved drugs.

To gain a more detailed understanding of the distribution of these physicochemical properties of *ent*-kaurane diterpenoids, individual physicochemical parameters were analyzed. The molecular weights varied from 288.47 to 626.82 g/mol. The median molecular weight was 366.45, whereas the mean was 382.72 g/mol (Figure 3a). In contrast, the FDA-approved drugs utilized for comparison had molecular weights varying from 60.06 to 972.66, a median of 278.32, and a mean of 302.30 (Figure 3b). The mean tally of heavy atoms among the scrutinized *ent*-kaurane diterpenoids stood at 27.37, exhibiting a notable diversity, and the minimum number of heavy atoms was 21, while the maximum number of heavy atoms was 44 (Figure 3c). The FDA-approved drugs used for comparison, on the other hand, displayed an average number of heavy atoms of 20.99, spanning from 3 to 68 (Figure 3d).

The mean tally of the number of hydrogen bond donors was 2.54 while that of FDA-approved drugs was 1.24 (Figure 3g,h). It is worth noting that some FDA-approved drugs exhibited anomalously higher quantum of hydrogen bond donors, reaching a zenith of 13 in comparison to the maximum value of 8 recorded for the sampled *ent*-kauranes. The number of hydrogen bond acceptors for the examined *ent*-kaurane diterpenoids displayed a range of 1 to 11 (Figure 3e) while that of FDA-approved drugs exhibited a broader range of 0 to 19 (Figure 3f). The mean number of hydrogen bond acceptors was 5.50, while that of FDA-approved drugs was 4.26, and the median values for *ent*-kauranes and FDA-approved drugs were 6 and 4, respectively. The number of rotatable bonds exhibited a span of 0 to a maximum of 11, with an average of 2.06 and a median of 2 (Figure 3i). On the other hand, among the FDA-approved drugs, the range extended from 0 to 24, with an average of 4.71 and a median of 4 (Figure 3j). The variation in fraction Csp3 values among the scrutinized *ent*-kaurane diterpenoids and FDA-endorsed pharmaceuticals exhibited extensive ranges, i.e., 0.65 to 1 (Figure 3k) and 0.00 to 1 (Figure 3l), respectively. The mean of this parameter for the surveyed *ent*-kaurane diterpenoids was 0.84, in stark contrast to the value of 0.41 for FDA-approved drugs. The median fraction Csp3 value observed for the sampled *ent*-kaurane diterpenoids was notably elevated at 0.85, compared to the corresponding value of 0.38 delineated for FDA-approved drugs. The mean TPSA observed among the sampled *ent*-kaurane diterpenoids in this study was 91.19 Å^2^, whereas the corresponding TPSA value for FDA-approved drugs was slightly lower at 76.65 Å^2^. The ranges were recorded at 20.23 to 189.53 Å^2^ for *ent*-kauranes (Figure 3m) and 0 to 379.82 Å^2^ for FDA-approved drugs (Figure 3n). The median TPSA values were 94.06 Å² for *ent*-kauranes and 67.40 Å^2^ for FDA-approved drugs.

### 2.3. Lipophilicity and Water Solubility of Ent-Kaurane Diterpenoids

**Figure 4:**
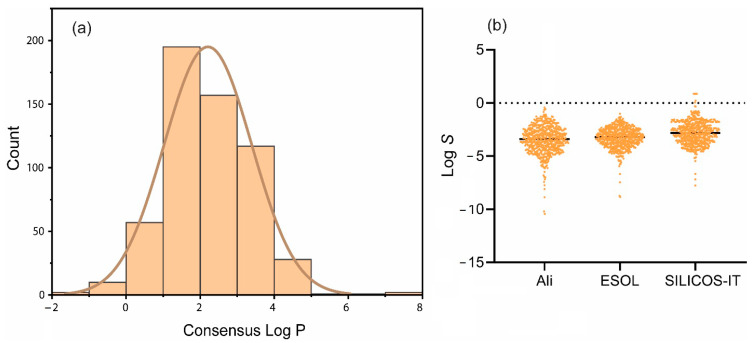
The lipophilicities (**a**) and water solubilities (**b**) of *ent*-kaurane diterpenoids.

The lipophilicity values spanned from −1.09 to a maximum of 7.58 with an average of 2.21. The distribution plot (Figure 4a) shows that most of these compounds possessed lipophilicities of ≤5, whereas water solubilities ranged from −10.44 to −0.4300 with an average of −3.46 according to the Ali approach. According to ESOL and SILICOS-IT approaches, the ranges were −8.85 to −2.01 and −7.77 to 0.86, and the means were −3.29 and −2.81, respectively (Figure 4b).

### 2.4. Pharmacokinetic Properties

**Figure 5:**
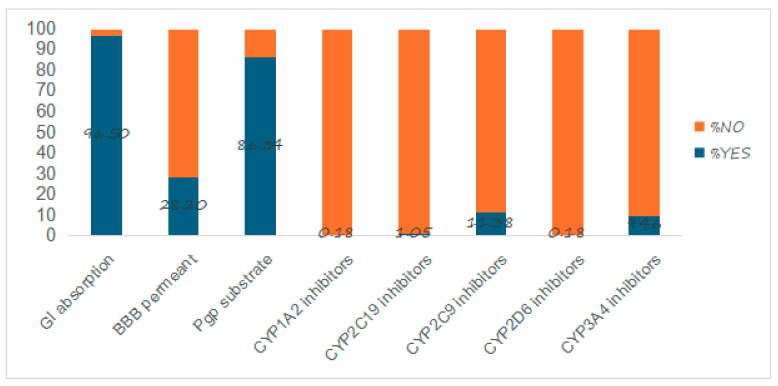
Stacked percentage column graph showing different pharmacokinetic properties of *ent*-kaurane diterpenoids.

Of the sampled molecules, 96.5% had high gastro-intestinal (GI) absorption, whereas the remaining subset was classified to possess low GI absorption. Only 28.20% of the molecules were predicted as blood–brain barrier (BBB) permeant. A majority of these molecules, i.e., 86.34%, were flagged as P-glycoprotein (P-gp) substrates. Further, a bigger subset did not inhibit the CYP1A2, CYP2C19, CYP2C9, CYP2D6, and CYP3A4 isoforms (Figure 5).

### 2.5. Toxicological Properties

**Figure 6:**
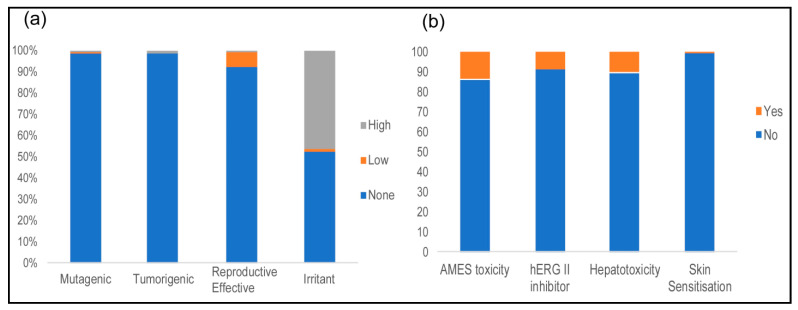
The toxicological properties of *ent*-kaurane diterpenoids: (**a**) stacked bar graphs showing the percent of mutagenic, tumorigenic, reproduction-enhancing, and irritant molecules determined using the DataWarrior and (**b**) stacked bar graphs showing the AMES toxicity, hERG II inhibition, hepatotoxicity, and skin sensitization properties determined using pKCSM.

The findings (Figure 6a) showed that the majority of the sampled compounds, accounting for 99.12%, do not exhibit mutagenic properties. A small fraction, constituting 0.70% of the compounds, was identified as highly mutagenic, while a further 0.18% exhibited low mutagenicity. In terms of tumorigenicity, 98.42% of the compounds displayed no tumorigenic potential, while 1.4% and 0.18% of the compounds exhibited high and low tumorigenic potentials, respectively. The determination of the reproductive effects revealed that 92.28% of the compounds have no discernible impact on reproduction, whereas 7.02% and 0.70% of the compounds were associated with low and high reproductive effects, respectively. Investigation of irritant properties revealed that a significant subset, 46.49% of the compounds, displayed high irritant properties, while the other subset was either devoid of irritant properties (52.36%) or had low irritant properties (1.05%).

AMES, hERG II inhibition, and hepatotoxicity determination using pKCSM revealed that a significant portion of these molecules are not toxic. Only 13.87%, 8.77%, 10.18%, and 0.35% were, in this respective order, flagged as AMES toxic, hERG II inhibitors, hepatotoxic, and skin sensitizers (Figure 6b). Not a single molecule was flagged as an hERG I inhibitor.

### 2.6. Synthetic Accessibility, PAINS, BRENK, and Lead-likeness

**Figure 7:**
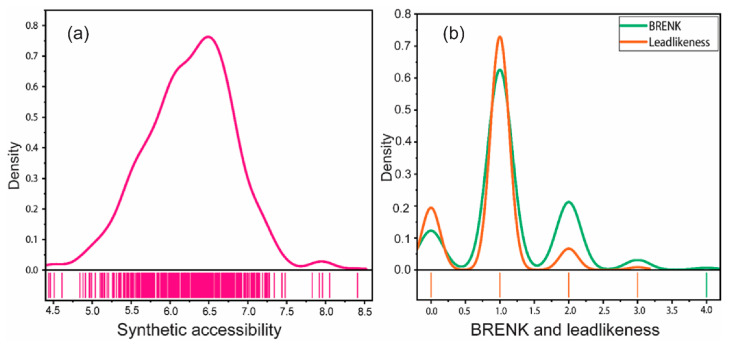
The distribution and rug plots showing the medicinal chemistry properties of *ent*-kaurane diterpenoids: (**a**) the synthetic accessibility and (**b**) BRENK and lead-likeness.

The synthetic accessibility scores for most of the studied *ent*-kauranes ranged from 5.0 to 7.5 (Figure 7a). Not a single *ent*-kaurane studied was flagged as PAINS. Approximately 64% of the examined compounds displayed a single BRENK alert, indicating the presence of a fragment possibly associated with unfavorable characteristics. Furthermore, about 5% of the molecules had two BRENK alerts and about 2% of the molecules had three BRENK alerts, suggesting a higher level of complexity in terms of potential undesired attributes. As shown in Figure 7b, about 74% of the studied *ent*-kauranes violated one rule of lead-likeness and about 5% violated two rules and about 1% violated three rules.

### 2.7. Bioactivity Scores and Macromolecular Targets of Selected Molecules

**Figure 8:**
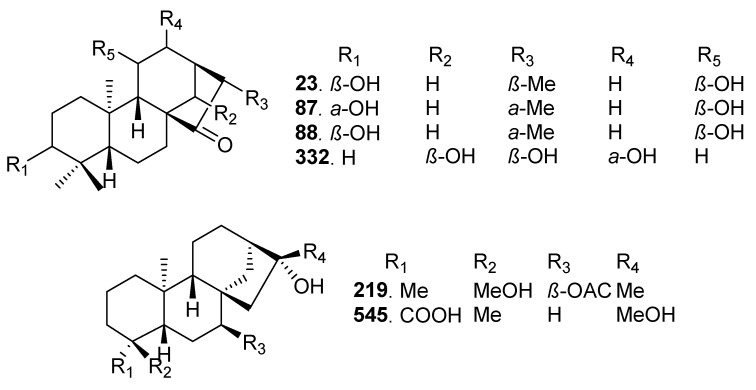
Selected drug-like *ent*-kaurane diterpenoids.

Six *ent*-kaurane diterpenoid molecules were selected for further investigation based on their adherence to drug-likeness criteria, specifically, the absence of any violations and demonstrated non-toxicity. These were 11*β*-hydroxy-*ent*-16-kauren-15-one (23) isolated from Jungermania tetragona, 16R-3α,11*β*-dihydroxy-ent-9(11)-kauren-15-one (87) and 16*S*-3*α*,11*β*-dihydroxy-*ent*-9(11)-kauren-15-one (88) isolated from *Jungermania* sp., *Ent*-7α-acetoxy-16*β*,18-dihydroxy-kaurane (219) isolated from *Sideris congesta*, Coetsanoic acid (332) isolated from *Isodon albopilosus,* and acid (545) isolated from *Siegesbeckia pubescens* (Figure 8).

As shown in Table 1, these molecules showed activity for the GPCR ligands and as ion channel modulators, nuclear receptor ligands, protease inhibitors, and enzyme inhibitors, and moderate activity as kinase inhibitors.

The further prediction of pharmacological activities of these representative molecules using PASS online revealed that *ent*-kaurane diterpenoids have immense potential biological activities including antineoplastic, antileukemic, apoptosis induction, immunosuppressant, chemoprotective, anti-hepatic, and hepatoprotectant activities, among others (Table 2).

**Figure 9:**
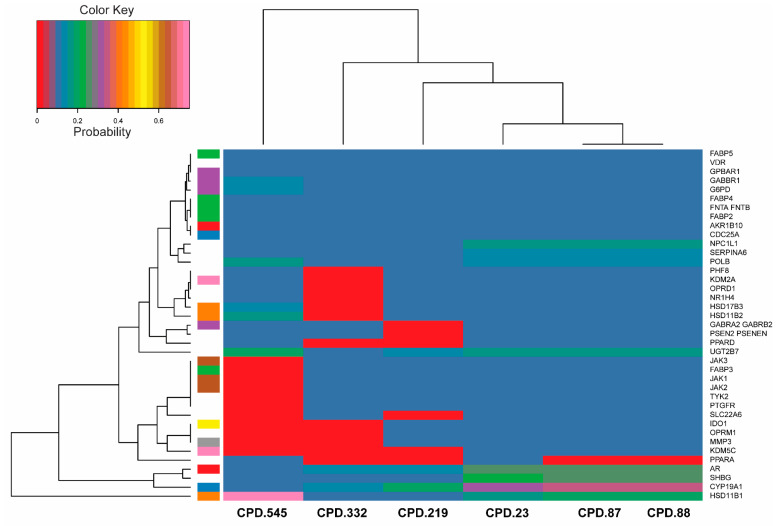
A heat map showing the macromolecular targets of selected *ent*-kaurane diterpenoids.

Macromolecular target prediction using the SwissTargetPrediction indicated that these molecules could also target enzymes associated with metabolic diseases (HSD11B1), could be aromatase inhibitors (CYP19A1), and exhibited anti-hepatitis and antihyperthyroidism (SHBG) activities, among others (Figure 9).

## 3. Discussion

### 3.1. Sources and Bioactivity of Ent-Kaurane Diterpenoids

The genesis of discovery of *ent*-kaurane diterpenoids can be traced back to the year 1961, when *ent*-kaurene was isolated from the leaf essential oil of *Agathis,* commonly known as the New Zealand Kauri pine [5]. This molecule was assigned the name “*ent*-kaurene” due to its negative optical rotation and enantiomeric nature, with “*ent*” denoting its enantiomeric status. Since then, a vast number of *ent*-kauranes with diverse bioactivities have been isolated and characterized, predominantly derived from plants of the *Isodon* family among the higher plants and also the *Jungermania* species of liverworts [5,37]. Synthetically and semi-synthetically, various other *ent*-kaurane diterpenoids have been accessed [38,39,40,41].

Biologically, a relatively higher number of sampled molecules have activities against leukemia and lung cancer, suggesting a potentially higher responsiveness of these cancer types to this class of molecules. Bioactivities against NO production and AchE and BchE inhibition were also found to be prevalent, further asserting the versatility and wider spectrum of bioactivities of this class of molecules. The widely accepted mechanism of action of these diterpenoids is the Michael addition of soft nucleophiles such as thiols and protein sulfihydryl (SH) groups onto the *α*,*β*-unsaturated ketone moiety, leading to the deactivation of SH enzymes or SH coenzymes and the accumulation of Reactive Oxygen Species (ROS) [37,42]. The deactivation of SH enzymes or SH coenzymes causes the retardation of intracellular redox homeostasis, while the accumulation of ROS, on the other hand, leads to apoptosis since high levels of ROS cause oxidative damage to tumor cells [37]. However, it is important to note that the bioactivity results presented are based on in vitro tests and translating these findings into effective and safe treatments for human use requires in-depth, laborious, and resource-intensive research, necessitating the use of computational chemistry as a filtration step. Further, these in vitro tests are often targeted to few specific targets, and this calls for polypharmacology prognostication. However, the pharmacological potential of a molecule is affected by several factors including physicochemical, pharmacokinetic, and toxicological properties.

### 3.2. Physicochemical Properties 

Molecular weight is an important physicochemical parameter that is considered in drug discovery as it directly affects the diffusion of molecules across cell membranes [43]. The Lipinski tenet, positing that an ideal molecular weight for effective absorption and distribution of a biomolecule should fall below 500 g/mol, serves as a foundational principle in drug designing [19]. Most *ent*-kauranes studied complied with this tenet, boasting molecular weights below 500 g/mol. However, a subset of these molecules deviated from this requirement, surpassing the mark. Notably, quite a number of FDA-approved drugs, a benchmark of pharmaceutical excellence, surpassed this mark by a bigger margin (max = 972.66 g/mol) as compared to the *ent*-kaurane diterpenoids (max = 626.82 g/mol). This divergence of FDA-approved drugs from Lipinski’s prescribed limit of molecular weight, yet being bioavailable molecules, suggests a multifaceted interplay with other molecular parameters. This beckons the need to unravel other complexities underlying successful drug absorption and distribution beyond the canons of Lipinski.

The number of heavy atoms refers to the collective count of non-hydrogen atoms within the chemical structure of a molecule. They typically include carbon, nitrogen, oxygen, sulfur, and other elements within the higher atomic masses. This physicochemical parameter wields a direct influence on ligand efficiency (LE), a metric calculated by dividing the binding free energy by the count of heavy atoms [44]. The relationship between ligand efficiency and drug potency is that ligand efficiency is a measurement of the binding energy per atom of a ligand to its binding partner such as an enzyme or receptor proteins, and therefore, a heightened LE postulates an elevated drug potency [44]. Based on the means, this study elicited that the sampled *ent*-kaurane diterpenoids generally had a higher number of heavy atoms than the FDA-approved drugs. However, certain FDA-approved drugs surpassed the heavy atom count of some sampled *ent*-kaurane diterpenoids, peaking at 68. This observation suggests that *ent*-kaurane diterpenoids, akin to FDA-approved therapeutics, could exhibit commendable ligand efficiencies if progressed towards drug development.

The quantitation of the numbers of hydrogen bond donors and acceptors stands as a pivotal physicochemical parameter in the prognostication of the oral bioavailability of a drug candidate [45]. By virtue of the influence of these parameters on the mechanisms of passive diffusion across cell membranes, they hold a discernible impact on the processes of adsorption and distribution of pharmaceutical agents. According to the tenets of Lipinski and Veber, a favorable number of hydrogen bond donors is not more than five, and the violation of this rule may indicate potential issues with bioavailability [19,46]. Some *ent*-kaurane diterpenoids sampled for this study violated this rule, and similar violations were also observed for chemotherapeutics already approved by the FDA. This underscores the fact that intricacies of drug development extend beyond the confines of a single parameter, and that those *ent*-kaurane diterpenoids violating this criterion should not be swiftly dismissed as they might harbor latent bioactivities. Alex et al. [47] highlights that more hydrogen bond donors than acceptors may engender deleterious effects on the intricate equilibrium of the drug’s membrane partitioning and permeability. However, this requisite is just a factor to consider in drug designing, and as such, several drugs violating this criterion are in the market such as acetaminophen, ibuprofen, aspirin, caffeine [48], and morphine [49].

The number of rotatable bonds assume a pivotal role in influencing biochemical and physicochemical characteristics of a therapeutic molecule. An excess of the number of rotatable bonds, above a threshold of nine, culminates in compromised oral bioavailability due to hindered diffusion through cell membranes [50,51]. This, in turn, often leads to diminished binding efficiency with the intended target, thereby undermining therapeutic potency. As outlined by Bryant et al. [52], molecules characterized by excessive flexibility may not conform to the criteria of drug-likeness. In this study, a substantial proportion of the investigated *ent*-kaurane diterpenoids exhibited a tally of rotatable bonds below the threshold of nine. Nonetheless, it is worth pointing out that a subset of these molecules displayed counts exceeding nine. However, within the domain of FDA-approved drugs, certain drugs showcased counts of rotatable bonds as high as 24, and yet they were qualified as potent therapeutics.

Fraction Csp3 is the ratio of sp^3^-hybridized carbons to the total carbon count of the molecule. This physicochemical parameter determines the carbon saturation of a molecule and characterizes the spatial structure of molecules. The empirical evidence showcases that the heightened saturation as quantified by the fraction of carbon atoms with sp^3^ hybridization (Csp3) correlates with an augmented rate of favorable clinical outcomes of pharmaceutical agents. This is because highly saturated molecules are more likely to be metabolized and eliminated from the body [53]. This phenomenon is conceivably associated with an elevation in solubility properties, and it has actually been previously used as a solubility predictor [54]. A suitable value of this physicochemical parameter for a drug-like molecule should be at least 0.42; however, just about 82% of marketed drugs meet this criterion [53]. The results emanating from this study impart a noteworthy implication that the assessed *ent*-kaurane diterpenoids exhibited a discernibly heightened state of saturation as opposed to the already approved drugs by the FDA. Evidently, in light of the stipulated threshold criterion (fraction Csp3 of ≥ 0.42), a considerably large number of the investigated *ent*-kaurane diterpenoids presented superior solubility and consequently superior drug-like properties in contrast to the majority of FDA-approved drugs.

The Topological Polar Surface Area (TPSA) of a molecule is defined as the sum of the contributions to the molecular surface area of polar atoms such as nitrogen, oxygen, and their attached hydrogens. This parameter has been shown to have a strong positive correlation with passive molecular transport through membranes and is advantageous as it does not require the generation of 3D structures [55]. It estimates the ability of a drug-like molecule to permeate cell membranes, and consequently, it has been used to study the intestinal absorption and blood–brain barrier penetration of therapeutic molecules [56]. As noted by Verber et al. [46], the TPSA of a suitable drug should be less than 140 Å^2^. A bigger subset of sampled *ent*-kaurane diterpenoids possessed a figure lower than 140 Å^2^, implying that this subset could be bioavailable. Remarkably, certain FDA-approved pharmaceutical agents exhibited TPSA values exceeding 140 Å^2^, thereby contravening the established criterion (TPSA < 140 Å^2^). This observation implies that solely relying on the TPSA metric might not be definitive for disqualifying prospective therapeutic agents, and that other parameters ought to be considered.

### 3.3. Lipophilicity and Water Solubilities

The lipophilicity and water solubility influences the bioavailability of a drug candidate. Lipophilicity, i.e., the *n*-octanol/water partition coefficient (log *P*_o/w_), is a measure of a compound’s capacity to dissolve in fats or lipids. This parameter wields significant control over molecular transportation across membranes, impacts binding to plasma proteins and target receptors, and thus serves as a cornerstone for predicting the biological efficacy of potential drugs. It is generally believed that drug molecules must be generally lipophilic to have good absorption [57]; however, the optimal lipophilicity for a drug-like molecule depends on the intended use and the target protein [58]. A compound that is too lipophilic may have poor solubility and bioavailability, while a compound that is too hydrophilic may have poor membrane permeability [59]. Lipophilicity exceeding five is often linked with undesired drug characteristics including limited water solubility, tissue accumulation, rapid metabolic turnover, and strong plasma protein binding [60,61,62]. Moreover, the literature indicates that compounds with moderate lipophilicity, oscillating around two, often show optimal abilities to reach molecular targets [60]. The mean of lipophilicities of the *ent*-kaurane diterpenoids studied was 2.21, suggesting that these compounds display promise as prospective candidates for drug design and research. 

To predict water solubility, SwissADME incorporates two topological approaches, i.e., the ESOL model [63] and the Ali model [64], and one fragmental approach developed by SILICOS-IT. However, the Ali model has a stronger positive correlation for the predicted water solubility with the experimental water solubility of a compound (R^2^  =  0.81), as compared to SILICOS-IT (R^2^  =  0.75) and the ESOL model (R^2^  =  0.69) [50]. Based on the solubility classification criteria provided by Daina et al. [50], i.e., insoluble < −10 < poorly < −6 < moderately < −4 < soluble < −2 < very < 0 < highly, the solubility of the *ent*-kaurane diterpenoids analyzed ranged from highly soluble to insoluble based on the Ali approach and highly soluble to poorly soluble based on SILICOS-IT and ESOL approaches. Only 0.35% of the compounds were labeled insoluble by the Ali approach. However, empirical evidence suggests that the target log *S* range of −1 to −5 for most drugs reflects a compromise desired to achieve aqueous solubility and, at the same time, hydrophobicity needed for a drug molecule to cross a membrane [65]. Arguably, a bigger subset of *ent*-kauranes studied were concentrated within this range, inferring that these compounds are amenable to crossing cell membranes and getting absorbed.

### 3.4. Pharmacokinetic Properties

Bioavailability is further influenced by pharmacokinetic properties including GI absorption, BBB permeability, P-gp substrates, and the inhibition of main cytochromes. The human GI absorption is quantified as the fraction of a given dose that has reached the portal vein. The extent of absorption by intestinal villi and microvilli determines how much of the administered drug enters the bloodstream. It is a multifaceted parameter influenced by several factors including the physicochemical state of the substance, GI physiology, formulation, and other biopharmaceutical factors [66]. Of the sampled molecules, 96.5% had high GI absorption, suggesting the high bioavailability of these molecules through the intestines. 

The blood–brain barrier (BBB) serves as a semipermeable and selective barrier, preventing the entry of unwanted external substances, and thereby maintaining the homeostatic balance of the central nervous system (CNS) [67]. The permeability of this membrane is maintained at extremely low levels, thus making the delivery of drugs to the brain very difficult [68]. The in silico determination of BBB permeability can help predict the performance of drugs in the CNS [69]. A small subset of *ent*-kaurane diterpenoids (28.20%) was predicted to be BBB permeants, suggesting that *ent*-kaurane diterpenoids are largely non-bioavailable to the brain. Nevertheless, this may not pose a significant challenge since several techniques have been devised to enable the delivery of non-BBB permeant drugs to the brain. These methods encompass the utilization of nanotechnology to encapsulate drugs within BBB-permeant nanoparticles, the use of ultrasound and microbubbles to disrupt the BBB, intranasal drug delivery, efflux transporters inhibition, and direct injections into the brain [70].

P-glycoprotein (P-gp) is a transmembrane protein that plays a crucial role in drug transport and efflux in various tissues, including the GI and the BBB [71]. This protein effluxes those drug substrates that it binds from enterocytes into the GI lumen, thereby limiting their bioavailability and efficacy [72]. Common drugs that are known to be P-gp substrates include vinblastine, indinavir, fostemsavir, mitapivat, etoposide, ritonavir, and digoxin [43]. The overexpression of the MDR1 gene responsible for the synthesis of this protein has been associated with the multidrug resistance of tumor cells [71]. Since this protein is substrate specific, it is imperative to determine if potential drug-like molecules can bind to it, as it helps in the prognostication of possible dosage for administration. A large number of molecules (86.34%) were found to be substrates of P-gp. This implies that a significant subset of these drugs is potentially recognized and effluxed from their targets by P-gp. This recognition will consequently lead to the reduced concentration of the active ingredients and bioavailability, particularly in the GI tract and across the BBB, thereby influencing their therapeutic efficacy in vivo. This result affirms the observed low levels of BBB permeability of the *ent*-kauranes. Interestingly, the GI absorption of these molecules was very high; overall, 96.50% of the studied molecules were flagged to have a high GI absorption. This is in tandem with reports that this protein is only localized to the apical membranes of the small intestines [71]. Therefore, other regions of the small intestines could serve as the alternative regions of absorption of these molecules, and hence, the observed high GI absorptivity of the studied molecules. These results underscore the importance of understanding the substrate–P-gp interactions and the possible ways of evading the binding or liberating the drug substrate from this protein, with a view of ensuring their successful therapeutic outcomes.

Understanding how molecules interact with cytochrome P450 (CYP) is pivotal in the realm of drug development and metabolism. Inhibition of these isoforms is a significant contributor to pharmacokinetics-related interactions between co-administered drugs. When one drug inhibits the activity of these enzymes, it can lead to alterations in the metabolism of the other drugs, potentially leading to their accumulation or production of other metabolites that differ from the original drug, which may be toxic [73]. Using in vitro bioluminescent assays, several P450 cytochrome inhibitors have been determined, and it became apparent that some compounds affect different CYP isoforms while others are selective to specific isoenzymes [74]. A significant number of *ent*-kaurane diterpenoids are the non-inhibitors of P450 cytochromes; thus, these molecules might have a lower likelihood of interfering with metabolism should they be co-administered with other therapeutics and therefore leading to lesser chances of causing toxicities.

### 3.5. Toxicological Properties

Computational toxicology evaluation is an important aspect in the early stages of drug discovery as it helps to identify potentially hazardous compounds. The upfront elimination of molecules with undesirable toxicological properties could help reduce the attrition and failure rates of drug candidates evaluated in drug discovery pipelines [75]. With regard to the studied toxicological endpoints, a significant number of *ent*-kaurane diterpenoids have minimal tumorigenic and mutagenic potentials, underscoring the promising safety profile of the compounds. These results, combined with the bioactivities of these molecules, substantiate the hypothesis that *ent*-kaurane diterpenoids have a great potential as candidates for further exploration and research in the domain of drug discovery.

### 3.6. Medicinal Chemistry: Synthetic Accessibility, PAINS, BRENK, and Lead-likeness

Synthetic accessibility (SA) refers to the ease with which a molecule can be synthesized. It is an important factor to consider when profiling compounds for drug discovery, as an intention to synthesize them may arise at later stages. The SA is normally expressed on a scale of 1 to 10, where a lower score indicates that a molecule is easier to synthesize, while a higher score suggests that synthesis might be more challenging [50]. Majority of the studied *ent*-kaurane diterpenoids could therefore be classified as moderately challenging to synthesize (SA: 5.0–7.5), and this could be attributed to their structural complexity or presence of certain functional groups that may require intricate synthetic steps. This augments the need to come up with novel reaction sequences or unique synthetic strategies to overcome synthetic challenges.

PAINS (Pan Assay Interference Compounds) are molecules that contain substructures showing potent responses in assays, regardless of the protein target. PAINS help identify potentially problematic fragments that may produce false positive responses in in silico assays [76]. Not a single studied *ent*-kaurane was flagged as a PAIN compound, suggesting that their interaction with various protein targets in in silico assays is less susceptible to generating misleading positive responses. The non-inclusion of the studied *ent*-kauranes in the PAINS category underscores the favorable prospect of utilizing these compounds in in silico assays without substantial concerns regarding the generation of false-positive results. 

On the other hand, BRENK alerts identify fragments of compounds that could be toxic, chemically reactive, or metabolically unstable, thus helping in identifying compounds that may have undesirable properties [76]. However, in-depth analysis revealed that the main motif that played a pivotal role in generating the observed heightened BRENK alerts (up to three) was the presence of Michael acceptors. This same motif is paradoxically the very motif that has been determined to confer cytotoxic and anticancer potential to these molecules.

Lead-likeness is a concept that focusses on identifying compounds that are suitable for optimization in drug discovery based on their physicochemical properties [77,78]. Approximately 74% of the compounds exhibited non-compliance with one lead-likeness rule, while around 5% of the compounds demonstrated non-compliance with two rules. Additionally, approximately 1% of the *ent*-kauranes violated three lead-likeness rules. The main physicochemical property of *ent*-kauranes leading to the heightened violation of the rules for lead-likeness was their molecular weights being higher than 350 Da. This means that a significant portion of these molecules are heavier than the recommended weight for lead-likeness and that these molecules could pose challenges during drug optimization processes.

### 3.7. Macromolecular Targets of Selected Molecules

Using Molinspiration cheminformatics, the bioactivity scores of these six molecules were predicted. This model encompasses various drug targets including G protein-coupled receptor (GPCR) ligands, kinase inhibitors, ion channel modulators, enzymes, and nuclear receptors. A higher bioactivity score indicates an increased probability of a compound exhibiting a biological activity. Consequently, a molecule with a bioactivity score exceeding 0.00 is deemed likely to possess biological activity, scores within the range of −0.50 to 0.00 are considered moderately active, and scores below −0.50 are presumed to indicate inactivity [79]. Importantly, the better the bioactivity score, the higher the possibility that the compound will be an active drug. The results showed that *ent*-kaurane diterpenoids exhibits activity for the GPCR ligands, as ion channel modulators, nuclear receptor ligands, protease inhibitors, and enzyme inhibitors, and have moderate activity as kinase inhibitors. Further prediction of pharmacological activities of these representative molecules using PASS online revealed that *ent*-kaurane diterpenoids have immense potential biological activities including antineoplastic, antileukemic, apoptosis induction, immunosuppressant, chemoprotective, anti-hepatic, and hepatoprotectant activities. If Pa score of a molecule is >0.7 on a specific target, it is expected that that molecule will show a high activity against that target [80]. Molecules displaying such potential are sought after, particularly for the management of chronic diseases and disorders. For example, aromatase inhibitors have been shown to be an effective and safer group of drugs for the first-line endocrine therapy of breast cancer [81], while HSD11B1 inhibitors are being explored as a potential strategy for managing conditions such as metabolic syndrome, diabetes, obesity, and inflammatory diseases [82]. Despite compounds 23, 87, and 88 being stereoisomers, they show different probabilities for different targets. The subtle difference in the spatial arrangement of their functional groups can influence their shape, flexibility, electronic properties, solvation, and other factors. These factors, in turn, can significantly impact how each epimer positions itself interacts with and fits into the binding pockets of the protein targets. This ultimately affects the overall binding probability.

## 4. Materials and Methods

### 4.1. Accessing Ent-Kaurane Diterpenoids and FDA-Approved Drugs

The words “*ent*-kaurane diterpenoids” were searched in the published literature such as Google Scholar, Web of Science, Science Direct, Pubmed, and Sci-Finder. The range used was customized to span from the year 1960 to 2023. The relevant and readable papers published in peer-reviewed journals and libraries such as Wiley, Elsevier, American Chemical Society, The Royal Society of Chemistry, etc. were downloaded and reviewed for the natural *ent*-kaurane diterpenoids, filtering out the synthetic and semi-synthetic analogues. To remove duplicates and repetitions, one compound from the dereplicated compounds was picked for this study. The chemical structures of *ent*-kauranes, as reported by the authors, were drawn using a licensed Chemdraw (Professional-2016). The structures were then converted to simplified molecular input line entry system (SMILES) to enable downstream analyses. The physicochemical properties of 1040 drug molecules approved by the United States’ Food and Drug Administration (FDA) were downloaded from the Enamine Bioactive Libraries “https://enamine.net/compound-libraries/bioactive-libraries (accessed on 28 September 2023)”.

### 4.2. Physicochemical and ADME Properties of Ent-Kaurane Diterpenoids 

The SwissADME (http://www.swissadme.ch/, accessed on 7 October 2023), a free online tool, was used to determine the physicochemical properties of the *ent*-kauranes and to predict their pharmacokinetic, lipophilicity, water solubility, and drug-likeness properties [50]. In batches of 100, the SMILES of the compounds were pasted in the “Enter list of SMILES here:” in the SwissADME webpage. The “Run!” icon was clicked, and the analyses were allowed to finish after which the data were exported into Excel in a CSV format. The Excel files for each batch of molecules were then matched.

### 4.3. Toxicological Properties of Ent-Kaurane Diterpenoids

The mutagenic, tumorigenic, reproduction-enhancing, and irritant properties were predicted using DataWarrior: an open-source program for data visualization and analysis with chemical intelligence (https://openmolecules.org/datawarrior/, accessed on 10 October 2023). AMES toxicity, hERG I and II inhibition, hepatotoxicity, and skin sensitization toxicities were predicted using the pKCSM web server (https://biosig.lab.uq.edu.au/pkcsm/, accessed on 10^th^ October 2023). In pKCSM, SMILES for all the molecules were submitted singly for toxicity prediction, and the results were recorded in an Excel spreadsheet. 

### 4.4. Selection of Molecules and Prediction of Their Macromolecular Targets

Molecules were chosen for further analysis by evaluating their physicochemical properties according to the rules set forth by Lipinski, Veber, Ghose, Muegge, and Egan. The Lipinski rule posits that a molecule is likely to be poorly absorbed if HBD > 5, HBA > 10, MW > 500 Da, and log P > 5 [19]. Ghose delineates drug-like attributes within the ranges of 0.4 < log P > 5.6, 160 < MW > 480, molar refractivity (MR) 40 < MR > 130, and a total atom count (TOC) 20 < TOC > 70 [83]. Veber’s rule introduces the concept that desirable oral bioavailability is attainable if a molecule exhibits an RTB of ≤10 and a TPSA of ≤ 140 Å^2^ [46]. Egan criteria posit that a promising oral drug candidate should manifest −1.0 ≤ log P ≤ 5.8 and TPSA ≤ 130 Å^2^. On the other hand, the Muegge criteria stipulate that a drug-like molecule should comply to the following: 200 < MW > 600 Da, −2 < XLogP > 5, TPSA < 150 Å^2^, RTB < 15, and HBD ≤ 5 [84]. *Ent*-kaurane diterpenoid molecules that did not violate these rules and that were not toxic were further scrutinized. The toxicological endpoints considered were hepatotoxicity, skin sensitization, AMES toxicity, tumorigenicity, mutagenicity, hERG I and II inhibitors, irritants, reproductive effectiveness, and BRENK alerts. The chosen molecules underwent the predictive analysis of their bioactivity scores, pharmacological activities, and most probable molecular targets, facilitated by the Molinspiration cheminformatics, PASS online, and SwissTargetPrediction websites. The visualization of data was accomplished by using software tools, including OriginPro (2023b), GraphPad (8.0.1.244), Past (4.06b), and R-vegan (2.6-4).

## 5. Conclusions

This study conducted a review of 570 natural bioactive *ent*-kaurane diterpenoids and the pharmacological effects of their physicochemical properties. The findings indicated that a substantial subset of these molecules demonstrates promising drug-like characteristics. The studied physicochemical parameters of the *ent*-kauranes fell within the ranges of the corresponding physicochemical parameters of the so far FDA-approved drugs, suggesting their potential suitability for drug development. While some compounds exhibited BRENK alerts and poor lead-likeness potential, none were marked as PAINS. Further, the majority of these diterpenoids do not strongly inhibit key cytochrome enzymes, suggesting a lower likelihood of interfering with drug metabolism and reducing the risk of toxic interactions when co-administered with other therapeutics. The observation that a significant subset of these molecules is GI permeant and is particularly promising for the development of drugs for oral administration. However, the low BBB permeability of a significant portion necessitates specialized delivery approaches to the brain cells. Synthetically, the majority of these compounds were classified as moderately challenging to synthesize, and this calls for better synthetic approaches for these compounds. The toxicity studies revealed that the majority of these compounds are not toxic. The curated six compounds demonstrated a diverse potential for the treatment of different cancers, hepatic disorders, and metabolic diseases. These findings underscore the potential of ent-kaurane diterpenoids as viable leads in drug discovery. This warrants further exploration towards the development of novel and efficacious pharmaceutical interventions, contributing to the achievement of Sustainable Development Goal (SDG) 3.

## Figures and Tables

**Table 1:** Bioactivity scores of drug-like *ent*-kaurane diterpenoid compounds against targets.

S/N	Compound	GPCR Ligand	Ion Channel Modulator	Kinase Inhibitor	Nuclear Receptor Ligand	Protease Inhibitor	Enzyme Inhibitor
1	23	0.43	0.36	−0.42	0.72	0.28	0.69
2	87	0.43	0.36	−0.42	0.72	0.28	0.69
3	88	0.43	0.36	−0.42	0.72	0.28	0.69
4	219	0.28	0.25	−0.23	0.79	0.31	0.53
5	332	0.19	0.19	−0.18	0.64	0.21	0.57
6	545	0.37	0.20	−0.19	0.62	0.20	0.53

**Table 2:** Probability of pharmacological activity of the selected *ent*-kaurane diterpenoids.

Activity	Probability of Pharmacological Activity (Pa)			
	Compounds 23, 87, and 88	Compound 219	Compound 332	Compound 545
Antineoplastic	0.946	0.957	0.969	0.712
Acylcarnitine hydrolase inhibitor	0.888	0.845	0.863	NA
Testosterone 17beta-dehydrogenase (NADP+) inhibitor	0.874	0.827	0.846	0.780
Apoptosis agonist	0.836	0.729	0.735	NA
Alkylacetylglycerophosphatase inhibitor	0.824	0.757	0.785	NA
Alkenylglycerophosphocholine hydrolase inhibitor	0.824	0.746	0.782	0.780
CYP2J substrate	0.815	0.832	0.849	0.702
Phosphatase inhibitor	0.780	NA	NA	NA
Antineoplastic (lung cancer)	0.777	0.858	0.899	NA
Caspase 8 stimulant	0.754	0.701	0.723	0.761
Oxidoreductase inhibitor	0.754	NA	NA	NA
Antinociceptive	0.742	NA	NA	NA
Caspase 3 stimulant	0.746	NA	NA	NA
Transcription factor NF kappa B stimulant	0.738	0.713	0.725	0.746
Transcription factor stimulant	0.738	0.713	0.725	0.746
Polarization stimulant	0.732	0.748	NA	NA
Antileukemic	0.731	NA	0.759	NA
CYP3A4 inducer	0.733	NA	NA	NA
Gluconate 2-dehydrogenase (acceptor) inhibitor	0.750	0.763	NA	NA
CYP2J2 substrate	0.740	NA	NA	NA
CYP3A inducer	0.720	NA	NA	NA
Myc inhibitor	0.711	NA	NA	NA
Glyceryl-ether monooxygenase inhibitor	0.711	NA	NA	NA
Immunosuppressant	0.701	NA	NA	NA
Antineoplastic (breast cancer)	NA	NA	0.740	NA
DNA polymerase I inhibitor	NA	NA	NA	0.716
Chemoprotective	NA	NA	NA	0.879
Hepatoprotectant	NA	NA	NA	0.879
Hepatic disorder treatment	NA	NA	NA	0.942

Values are Pa scores, while NA represents not active (Pa < 0.7).

## Data Availability

Data are contained within the article and Supplementary Materials.

## Supplementary Materials

The following supporting information can be downloaded at: https://www.mdpi.com/article/10.3390/ph17040510/s1, Supplementary Information S1: Chemical structures of *ent*-kaurane diterpenoids, Supplementary Information S2: Sources and bioactivities of *ent*-kaurane diterpenoids, and Supplementary Information S3: Bioactivities and molecular descriptors of *ent*-kaurane diterpenoids.
