# Supplementary material for: Unveiling the Potential of Ent-Kaurane Diterpenoids: Multifaceted Natural Products for Drug Discovery
Source: Pharmaceuticals (Basel). 2024 Apr 16;17(4):510. doi: 10.3390/ph17040510 (PMC11054903; doi:10.3390/ph17040510)
Supplement: Supplementary file 1 [file pharmaceuticals-17-00510-s001.zip › pharmaceuticals-2941103 final Supplementary Information -S1 -S2 -S3.pdf]

Review

Unveiling the Potential of *Ent*-kaurane Diterpenoids: Multifaceted Natural Products for Drug Discovery

Shadrack Kibet [1,2], Njogu M. Kimani [1,3*], Syombua S. Mwanza [1,2], Cynthia Muhavi Mudalungu [2,4*], Cleydson B. R. Santos [5,6] and Chrysantus Mbi Tanga [2*]

1 University of Embu, Department of Physical Sciences, P.O Box 6-60100, Embu, Kenya.
2 International Centre of Insects Physiology and Ecology, P.O Box 30772-00100, Nairobi, Kenya.
3 Natural Product Chemistry and Computational Drug Discovery Laboratory, P.O. Box 6-60100, Embu, Kenya.
4 School of Chemistry and Material Science, The Technical University of Kenya, P.O Box 52428-00200, Nairobi, Kenya.
5 Graduate Program in Medicinal Chemistry and Molecular Modelling, Health Science Institute, Federal University of Pará, Belém 66075-110, PA, Brazil.
6 Laboratory of Modelling and Computational Chemistry, Department of Biological and Health Sciences, Federal University of Amapá, Macapá 68902-280, AP, Brazil.
* Correspondence: 1. Cynthia M. Mudalungu; cmudalungu@icipe.org; Tel.: +254714971792
2. Njogu M. Kimani; njogu.mark@embuni.ac.ke; Tel.: +254728132596
3. Chrysantus M. Tanga; ctanga@icipe.org; Tel.: +254702729931

Supporting Information

Citation: To be added by editorial staff during production.

Academic Editor: Firstname Lastname

Received: date
Revised: date
Accepted: date
Published: date

Copyright: © 2024 by the authors. Submitted for possible open access publication under the terms and conditions of the Creative Commons Attribution (CC BY) license (https://creativecommons.org/licenses/by/4.0/).

SUPPLEMENTARY INFORMATION S1: Chemical Structures of *ent*-kaurane diterpenoids

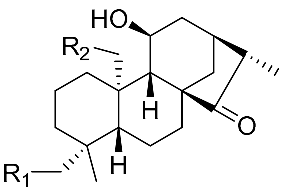

	R_1	R2
1.	OAc	H
2.	OAc	OH
3.	H	OAc
4.	OAc	OAc
5.	H	OH
6.	OH	OAc
7.	H	H

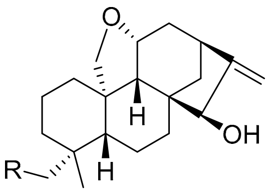

	R
27.	H
28.	OAc

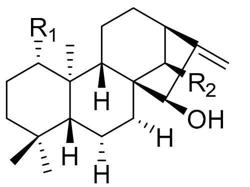

	R_1	R_1
29.	OH	H
30.	H	OH

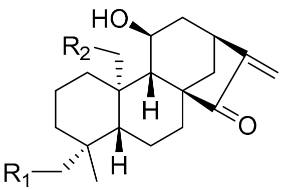

	R_1	R_2
8.	OAc	OAc
9.	OAc	OH
10.	H	OH
11.	OAc	H
12.	H	OAc
13.	H	H

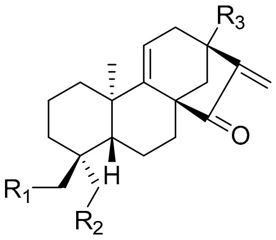

	R_1	R_2	R_3
31.	OH	H	OH
32.	CHO	H	OH
33.	H	OH	OH
34.	OH	H	H
35.	H	H	OH

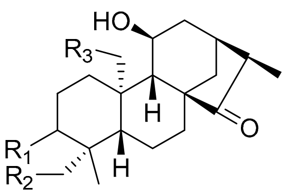

	R_1	R_2	R_3
14.	H	OAc	OH
15.	H	H	OAc
16.	H	OAc	OAc
17.	H	OAc	H
18.	H	H	H
23.	β-OH	H	H

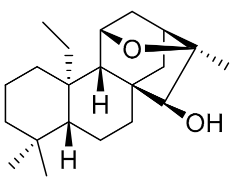

19

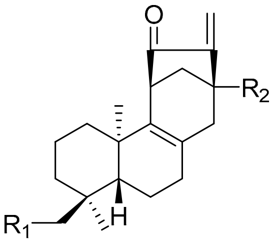

	R_1	R_2
36.	H	OH
37.	OH	OH
38.	OH	H

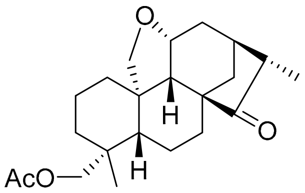

24

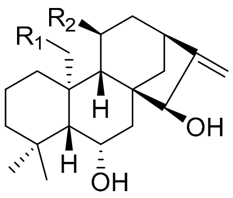

	R_1	R_2
25.	OAc	OH
26.	H	H

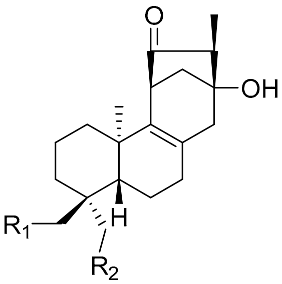

	R1	R2
39.	OH	H
40.	H	H
41.	CHO	H
42.	H	OH

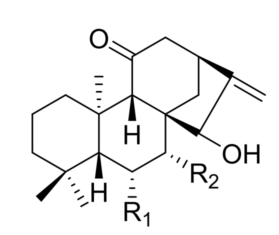

R1	R2
43. OH	H
44. OH	OH
45. H	OH
46. H	H

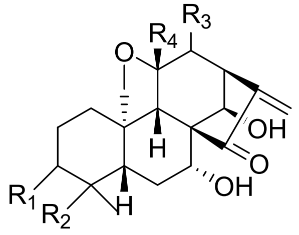

R_1	R_2	R_3	R_4
56. β-OAc	Me	α-OH	OH
58. H	β-CHO	H	OMe
59. H	Me	H	OH

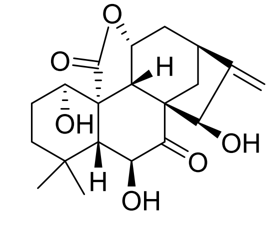

60

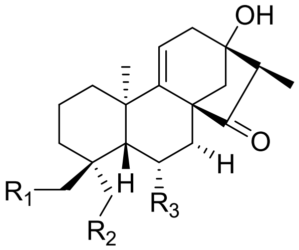

R_1	R_2	R_3
47. H	H	H
48. OH	H	H
49. CHO	H	H
50. H	OH	H
51. OH	H	OH

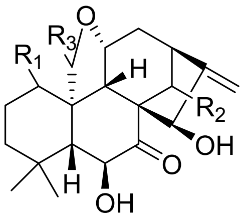

R_1	R_2	R_3
61. α-OH	H	β-OH
62. 2H	β-OH	H
63. α-O	H	β-Me

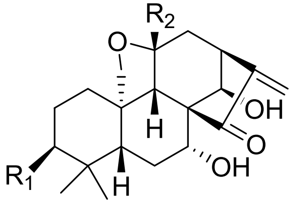

R_1	R_2
52. OAc	OMe
53. OAc	OH

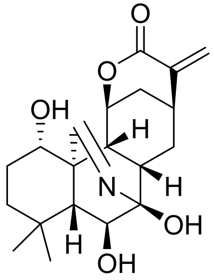

64

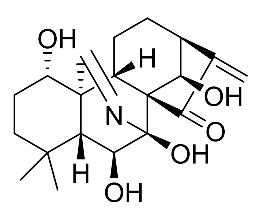

65

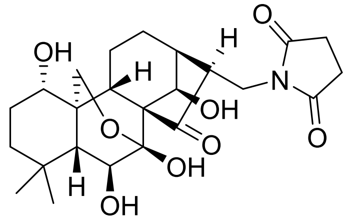

66

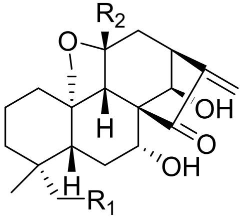

R_1	R_2
54. OAc	OMe
55. OAc	OH

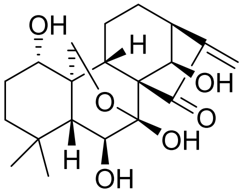

67

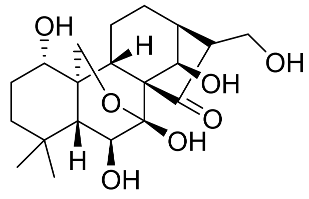

68

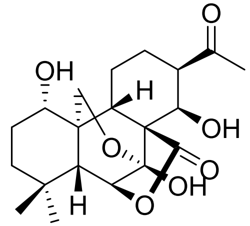

69

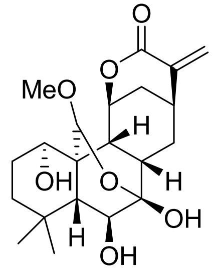

70

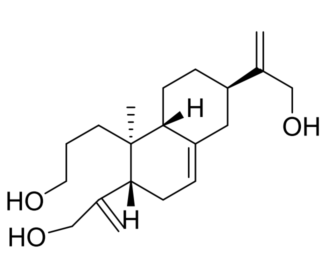

74

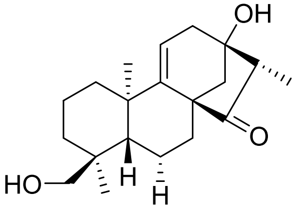

79

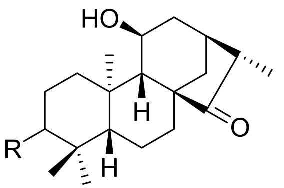

	R
87.	*a*-OH
88.	*ß*-OH
89.	2H

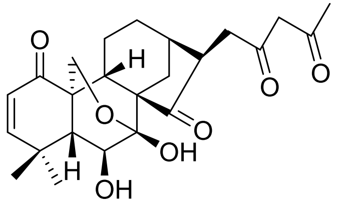

71

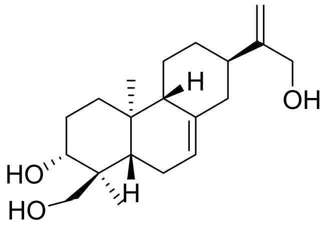

75

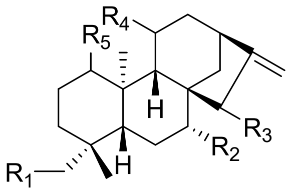

	R_1	R_2	R_3	R_4	R_5
80.	3H	H	β-OH	H	α-OH
81.	3H	H	α-OH	H	α-OH
82.	3H	OH	H	H	α-OH
83.	3H	OAc	H	H	α-OH
84.	3H	Me	β-OH	α-OH	2H
85.	3H	Me	β-OH	α-OH	2H
86.	CH_2OH	Me	β-OH	α-H	2H

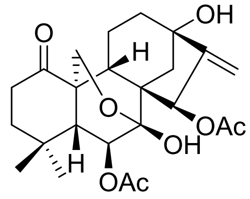

72

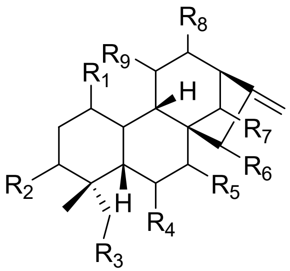

	R_1	R_2	R_3	R_4	R_5	R_6	R_7	R_8	R_9
76.	α-H	H	H	α-H	α-OH	β-OH	β-H	H	H
77.	α-OH	H	H	α-OH	α-H	β-OH	β-H	H	H
78.	α-H	H	H	α-H	α-H	β-OH	β-H	H	H
272.	α-H	β-OAc	H	H	α-OH	=O	β-OH	α-OH	H
273.	α-H	H	OAc	α-OH	α-OH	=O	β-H	H	α-H
278.	α-OH	β-H	H	H	α-OH	=O	β-OH	α-OAc	H

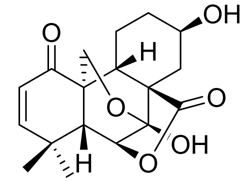

73

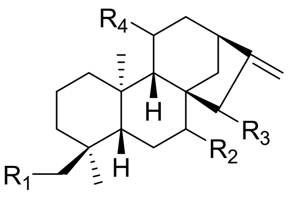

	R_1	R_2	R_3	R_4
90.	H	H	=O	β-OH
91.	H	H	β-OH	β-OH
92.	COOH	H	β-OAc	H
93.	COOH	H	H	H
95.	H	α-OH	α-OH	H
96.	H	α-OH	H	H

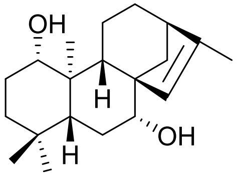

94

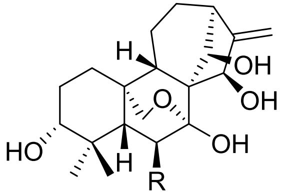

	R
109.	OH
113.	OAc

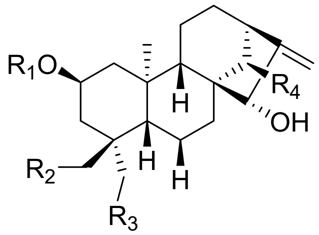

R1	R2	R3	R4
97. H	H	H	H
98. Glc	H	H	H
99. H	MeOH	H	H
100. Glc	MeOH	H	H
101. Glc	H	MeOH	H
102. H	H	H	H
103. H	H	H	OH
104. Glc	H	H	OH

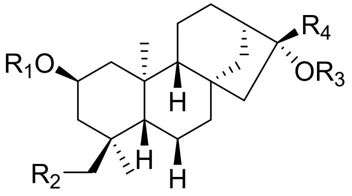

R1	R2	R3	R4
105. H	H	H	Me
106. Glc	H	Glc	Me
107. H	OH	H	Me
108. Glc	H	H	MeOH

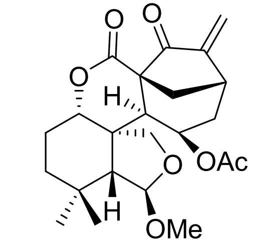

110

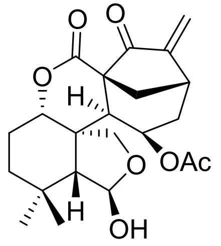

114

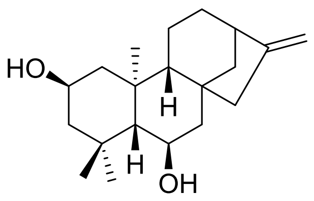

117

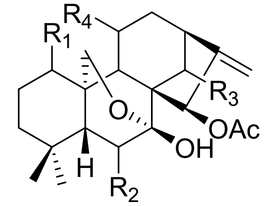

R1	R2	R3	R4
122. H	β-OAc	β-OAc	H
123. H	β-OH	β-OAc	β-OH
124. β-OH	β-OH	β-OAc	H
169. α-OH	β-OH	H	β-OGlc
170. α-OGlc	β-OH	β-OH	H
171. α-OGlc	β-OH	H	H
172. H	β-OGlc	α-OH	β-OH
173. α-OGLc	β-OH	H	β-OH
174. α-OGLc	β-OH	α-OH	H

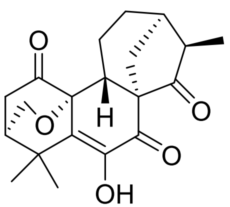

111

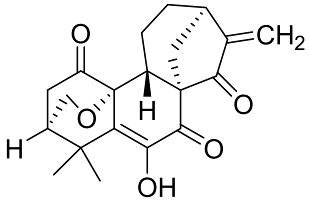

115

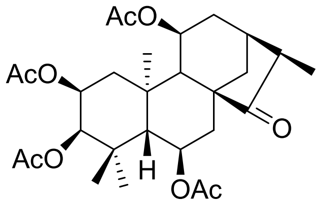

121

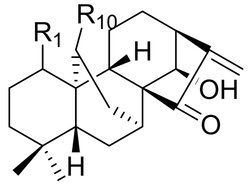

R1	R2
165. α-OH	OEt
166. α-OH	OMe

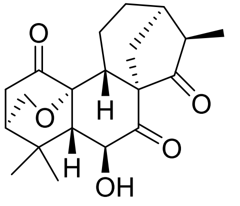

112

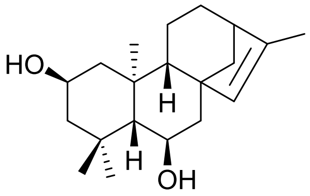

116

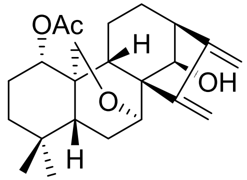

162

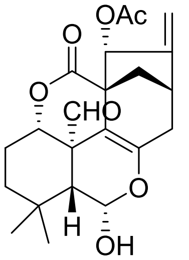

177

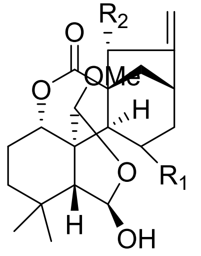

R1	R2
178. H	OAc
179. α-OH	OAc
180. α-OH	OH

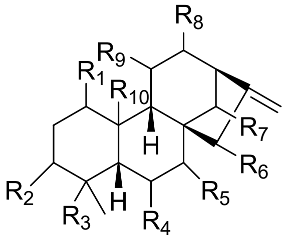

	R_1	R_2	R_3	R_4	R_5	R_6	R_7	R_8	R_9	R_{10}
20.	H	β-H	α–Me	H	H	β-OH	H	H	β-OH	α-Me
21.	H	β-H	α–Me	H	H	β-OH	H	H	β-OH	α-Me
22.	H	β-OAc	α–Me	H	H	β-OH	H	H	β-OH	α-Me
125.	H	H	β-MeOH	H	α-OAc	=O	β-OH	α-OH	H	α-Me
126.	H	H	β-MeOH	H	α-OH	=O	β-OAc	α-OH	H	α-Me
127.	H	H	β-OAc	H	α-OAc	=O	β-OH	α-OH	H	α-Me
128.	H	H	β-OH	H	α-OH	=O	β-H	α-OH	H	α-Me
129.	H	H	β-OH	H	α-OH	=O	β-OH	α-OH	H	α-Me
130.	H	α-OH	Me	H	α-OH	=O	β-OH	α-OH	β-OH	α-Me
131.	H	α-OH	β-MeOH	H	α-OH	=O	β-OH	α-OH	β-OH	α-Me
132.	H	=O	β-MeOH	H	α-H	β-OH	β-H	H	β-H	α-Me
133.	H	OH	β-MeOH	H	α-H	=O	β-H	α-OH	β-H	α-Me
134.	H	α-H	Me	H	α-OAc	β-OH	β-OH	α-OH	β-H	α-Me
135.	H	α-H	Me	α-OH	α-H	=O	β-OH	α-H	β-OH	α-Me
136.	H	α-H	Me	=O	α-H	=O	β-OH	α-H	β-OH	α-Me
137.	H	H	β-MeCOH	H	α-H	=O	β-OH	α-OH	H	α-Me
138.	H	H	β-MeOH	H	α-H	β-OH	β-H	α-OH	H	α-Me
139.	H	H	β-MeOH	H	=O	β-OH	β-OH	H	β-H	α-Me
140.	H	H	β-MeOH	H	α-OH	=O	β-OH	α-OH	H	α-MeOH
141.	H	H	β-MeOH	H	H	=O	β-OH	H	H	α-CHO
142.	H	H	β-MeOH	H	α-OH	=O	H	H	H	α-MeOH
143.	α-OAc	β-OH	Me	α-OAc	β-OAc	β-OH	H	H	β-OAc	α-Me
144.	α-OAc	β-OH	Me	=O	β-OAc	β-OH	H	H	β-OAc	α-Me
145.	α-OAc	β-OH	Me	=O	β-OH	β-OAc	H	H	β-OAc	α-Me
146.	α-OAc	β-OH	Me	=O	β-OH	β-OH	H	H	β-OAc	α-Me
147.	α-OAc	β-OH	Me	=O	β-OH	β-OH	H	H	β-OAc	α-Me
148.	H	H	Me	H	α-OAc	=O	β-OH	α-OH	β-OH	α-Me
149.	H	H	Me	H	α-OH	=O	β-OH	α-OH	H	α-Me
150.	H	H	Me	H	α-OH	=O	β-OH	H	β-OH	α-Me
161.	α-OH	H	β-MeOAc	H	α-OH	=O	β-OH	α-OH	H	H
163.	α-OH	H	Me	H	α-OH	=O	β-OH	α-OH	β-OH	α-Me
164.	α-OH	H	Me	H	α-OH	=O	β-OH	α-OH	β-OH	α-Me
202.	H	H	β-MeOH	H	H	=O	β-OH	α-OH	H	α-Me
203.	H	H	β-MeOH	H	α-OH	=O	β-OH	H	β-OH	α-Me
204.	α-OAc	H	β-CHO	H	α-OH	=O	β-OH	α-OH	H	α-Me
205.	α-OAc	H	Me	H	α-OH	=O	β-OH	α-OH	β-OH	α-Me
206.	H	H	β-MeOH	H	α-OH	α-OAc	β-OH	H	H	H
207.	α-OAc	b-OH	β-MeOH	H	α-OH	=O	β-OH	=O	H	H
208.	α-OH	H	β-Me	H	α-OH	=O	β-OH	H	H	α-MeOAc

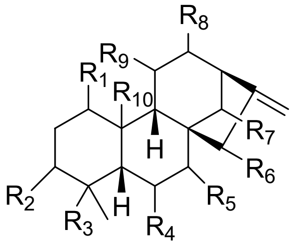

209. α-OAc	H	β-MeOH	H	α-OH	=O	β-OH	H	H	α-MeOAc
210. α-OH	H	β-Me	H	α-OH	=O	β-OH	H	H	α-MeOAc
211. α-H	H	β-Me	H	α-OH	=O	β-OH	H	H	α-Me
215. α-OH	α-OH	Me	H	H	=O	H	H	β-OH	α-Me
216. α-OAc	β-OH	Me	H	H	β-OH	H	H	β-OH	α-Me
217. α-OAc	β-OH	Me	H	=O	β-OAc	H	H	β-OAc	α-Me
218. α-OH	β-OH	Me	H	H	β-OH	H	H	β-OAc	α-Me
	β-OH	Me	=O	β-OH	β-OH	H	H	β-OH	α-Me
	β-OH	Me	=O	β-OH	β-OH	H	H	β-OH	α-Me
	β-OH	Me	=O	β-OH	β-OH	H	H	β-OAc	α-Me
	β-OH	Me	=O	β-OH	β-OH	H	H	β-OH	α-Me
	β-OH	Me	=O	β-OH	β-OH	H	H	β-OH	α-Me
	β-OH	Me	=O	β-OH	β-OH	H	H	β-OAc	α-Me
	β-OH	Me	=O	β-OH	β-OH	H	H	=O	α-Me
	β-OH	Me	=O	β-OH	=O	H	H	β-OAc	α-Me
	β-H	Me	=O	β-OH	=O	H	α-OH	β-OH	α-Me
	β-OAc	Me	H	β-OAc	=O	H	H	β-OH	α-Me
	β-OH	Me	H	β-OAc	=O	H	H	β-OH	α-Me
	β-OH	Me	H	β-OAc	β-OH	H	H	β-OAc	α-Me
	β-OAc	Me	H	β-OAc	β-OH	H	H	β-OH	α-Me

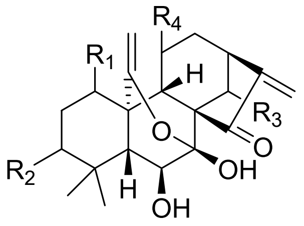

	R_1	R_2	R_3	R_4
181.	H	H	β-OAc	β-OAc
193.	H	β-H	β-OH	α-OH
194.	α-OAc	β-H	β-OH	α-OH
195.	α-OH	β-H	β-OH	H
196.	α-OH	β-H	β-OH	α-OAc

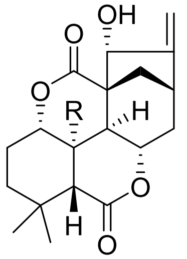

	R
176.	OH
192.	CHO

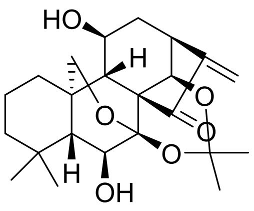

201

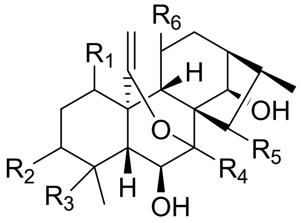

	R_1	R_2	R_3	R_4	R_5	R_6
182.	β-OH	H	α-MeCHO	β-OH	=O	H
183.	β-OH	β-OH	α-MeCHO	β-H	=O	H
184.	H	β-H	Me	β-H	β-OH	β-OH
185.	H	β-H	Me	β-H	β-OH	H

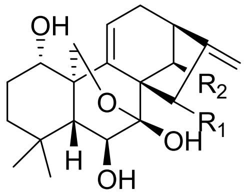

	R_1	R_2
186.	b-OH	b-OH
187.	=O	b-H

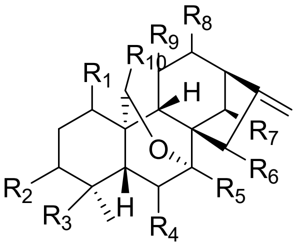

	R1	R2	R3	R4	R5	R6	R7	R8	R9	R10
167.	α-OH	H	β-Me	H	H	=O	β-OH	H	H	OMe
168.	α-OH	H	β-Me	β-OH	β-OH	=O	β-OH	H	H	H
175.	α-OH	H	β-Me	β-OH	β-OH	β-OH	α-OH	H	H	H
188.	α-OH	H	Me	β-OH	β-OH	=O	β-OH	H	β-H	α-OEt
189.	α-OH	H	Me	β-OH	β-OH	=O	β-OH	H	H	β-OEt
190.	α-OH	H	Me	β-OH	β-OH	=O	β-OH	H	H	β-OBu
191.	α-OH	H	Me	=O	β-OH	=O	β-OH	H	H	OH
197.	α-OH	β-H	Me	β-OH	β-OH	β-OH	β-OH	H	H	=O
198.	α-OH	H	Me	β-OH	β-OH	=O	β-OH	H	β-H	β-OMe
199.	α-OH	H	Me	β-OH	β-OH	=O	β-OH	H	β-H	α-OMe
200.	α-OMe	H	Me	β-OH	β-OH	β-OH	β-OH	H	β-H	OMe
230.	H	H	β-Me	β-OAc	β-OH	β-OH	H	b-OH	H	H
234.	H	H	β-Me	β-OAc	β-OH	β-OH	H	H	H	H
235.	H	H	β-Me	β-OH	β-OH	=O	H	α-OAc	H	H
236.	α-OH	H	β-Me	β-OH	β-OH	β-OAc	H	H	β-OH	H
238.	H	H	β-Me	β-OH	β-OH	β-OH	H	β-OH	H	H
239.	H	H	β-Me	β-OAc	β-OH	β-OH	β-OH	H	H	H
240.	b-OH	H	β-Me	β-OAc	β-OH	β-OAc	β-OH	H	H	H
249.	α-OH	H	β-Me	β-OH	β-OH	=O	H	H	α-OH	H

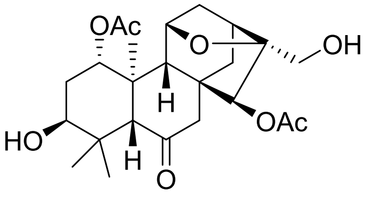

212

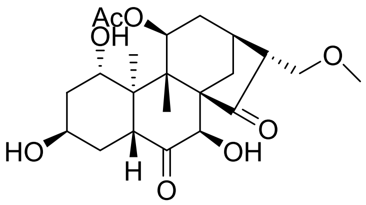

213

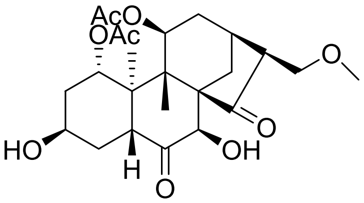

214

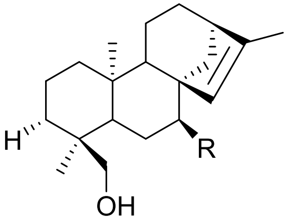

R	
222.	OH
223.	OOAc

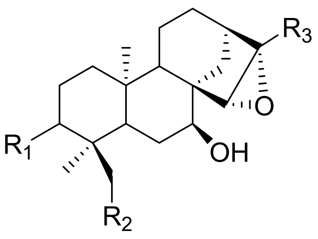

	R1	R2	R3
220.	β-OH	OOAc	H
221.	α-H	OH	β-Me

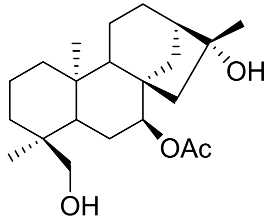

219

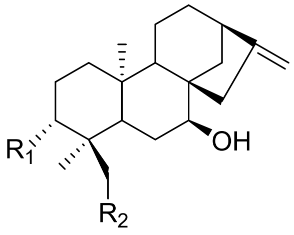

	R1	R2
224.	H	OH
225.	OH	OH
226.	OH	OAc
227.	OAc	OH

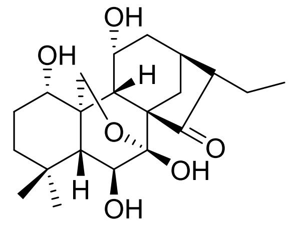

228

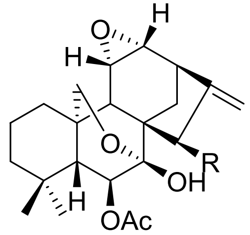

R
231. OAc
243. OH

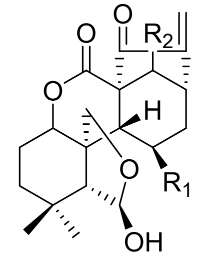

232

R1	R2
232. OAc	H
237. OH	H
248. H	a-OH

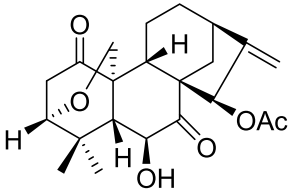

233

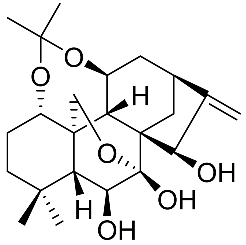

246

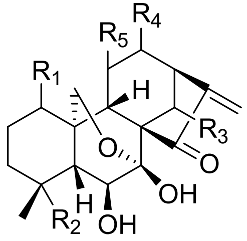

R1	R2	R3	R4	R5
229. H	α-MeOAc	α-OH	α-OH	H
245. α-OH	α-MeOAc	H	H	α-OH
247. H	α-MeOAc	H	α-OH	H
251. H	α-MeOAc	β-OH	H	H
267. α-OAc	α-MeOAc	β-OH	H	α-H
268. α-OH	α-MeOAc	β-H	H	α-OAc

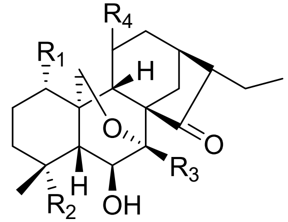

R1	R2	R3	R4
242. H	MeOAc	β-OH	α-OAc
250. OH	Me	H	β-OH

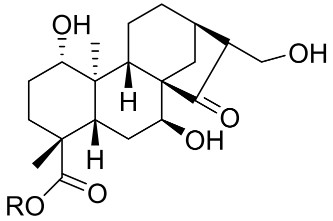

R
257. H
262. Me

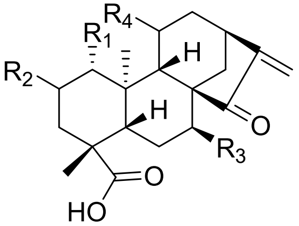

R1	R2	R3	R4
252. OH	α-H	OH	β-H
253. H	α-H	OH	β-H
254. =O	H	OH	H
255. H	α-H	OH	β-OH
256. OH	α-OH	OH	β-H
263. OAc	α-H	OH	β-H
264. OAc	α-H	OAc	β-H

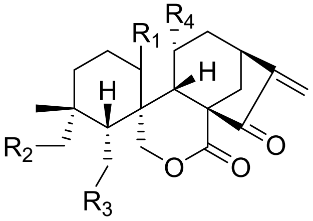

R1	R2	R3	R4
241. H	H	CHO	α-OH
244. α-OAc	H	CHO	β-OH
269. α-Me	OAc	OH	α-H
270. α-Me	OH	OH	α-H
271. α-Me	OAc	CHO	α-H
281. α-Me	OAc	CHO	α-OH

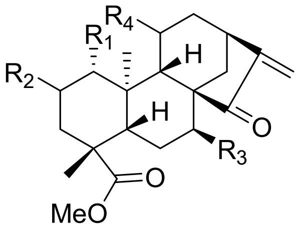

	R1	R2	R3	R4
258.	α-OH	α-H	β-OH	β-H
259.	α-H	α-H	β-OH	β-H
260.	=O	H	β-OH	H
261.	α-OH	α-OH	β-OH	β-H
265.	α-OAc	α-H	β-OH	β-H
266.	α-OAc	a-H	β-OAc	β-H

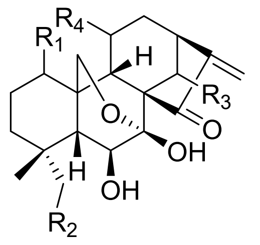

	R1	R2	R3	R4
274.	α-OH	OAc	β-H	α-H
275.	α-H	OAc	β-OH	α-H
276.	α-H	OAc	β-H	α-OH
277.	α-OAc	OAc	β-H	α-H
279.	α-OAc	H	β-OH	α-H
280.	α-OH	H	β-OH	α-H

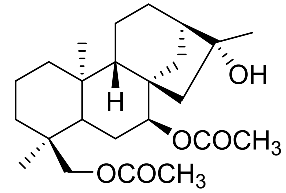

282

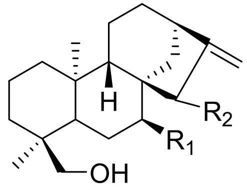

283

	R1	R2
283.	OAc	β-OH
284.	OAc	α-OH
285.	OH	β-OH
287.	OH	H

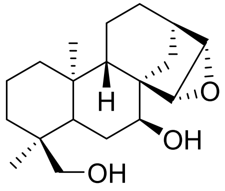

286

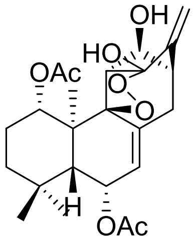

288

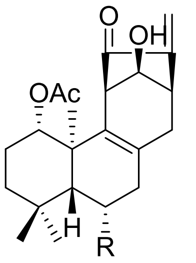

	R
289.	OAc
290.	OH

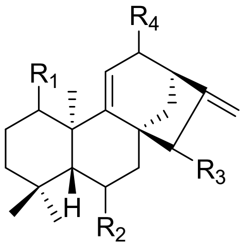

	R1	R2	R3	R4
291.	α-OAc	α-OAc	=O	=O
293.	α-OAc	α-OAc	=O	β-OH
294.	α-OH	α-OAc	=O	β-OH
297.	H	α-OAc	β-OAc	=O

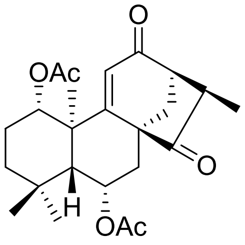

292

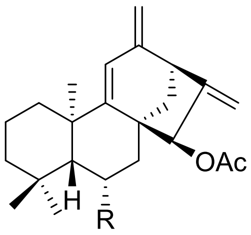

	R
295.	OH
296.	OAc

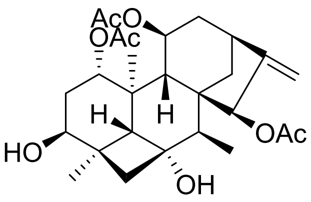

298

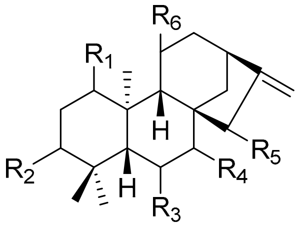

	R_1	R_2	R_3	R_4	R_5	R_6
299.	α-OH	β-OH	α-OAc	β-OAc	=O	β-OAc
300.	α-OH	β-OH	=O	β-OAc	β-OAc	β-OAc
301.	α-OAc	β-OH	a-OH	β-OAc	β-OAc	β-OAc
302.	α-OAc	β-OH	=O	β-OH	β-OH	β-OAc
303.	α-OAc	β-OH	α-OH	β-OAc	β-OH	β-OH
304.	α-OAc	β-OH	α-OAc	β-OAc	β-OH	β-OH
305.	α-OH	β-OH	H	β-OAc	β-OH	β-OAc
306.	α-OH	β-OH	a-OH	β-OAc	β-OH	β-OAc
307.	α-OH	β-OH	=O	β-H	β-OAc	β-OH
308.	α-OAc	β-OH	α-OH	β-OAc	=O	β-OAc
309.	α-OAc	β-OH	=O	β-OAc	β-OAc	β-OAc
310.	α-OAc	β-OH	=O	β-OH	β-OAc	β-OAc
311.	α-OH	β-OAc	α-OH	β-OAc	β-OH	β-OH
312.	α-OH	β-OAc	H	β-OAc	β-OH	β-OH
313.	α-OAc	β-OH	α-OH	β-OAc	=O	β-OAc
314.	α-OAc	β-OH	=O	β-OAc	=O	β-OAc
315.	α-OAc	β-OH	β-OH	β-OAc	β-OH	β-OAc
316.	α-OAc	β-OH	=O	β-OAc	β-OH	β-OAc
317.	α-OH	β-OH	=O	β-OAc	=O	β-OAc

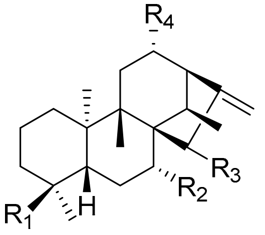

	R1	R_2	R_3	R_4
318.	MeOH	α-OH	β-OAc	α-OH
319.	CHO	α-OH	β-OAc	α-OH
320.	MeOH	α-OH	β-OAc	α-H
323.	MeOH	α-OH	β-H	α-OH
324.	CHO	α-OH	=O	α-H
325.	COOH	α-OH	=O	α-H
327.	AcOHC	α-OH	=O	α-OH
328.	CHO	α-OH	=O	α-OH
329.	MeOH	α-OH	=O	α-OH
330.	MeOH	α-OH	=O	α-H
331.	COOH	α-OH	=O	α-OH

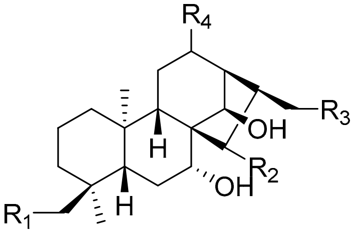

	R_1	R_2	R_3	R_4
321.	OH	β-OH	H	=O
322.	OAc	=O	OH	α-OH
326.	Glc	=O	OH	α-OH
332.	H	=O	H	α-OH

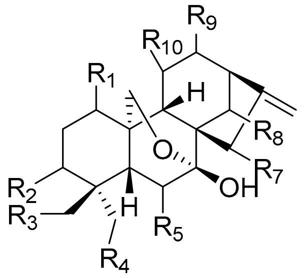

	R1	R_2	R_3	R_4	R_5	R_6	R_7	R_8
334.	α-H	H	H	OAc	β-OH	β-OH	β-H	H
335.	α-H	H	OH	H	β-H	β-OH	β-OH	H
350.	α-OH	H	H	H	β-OH	=O	β-OH	H
351.	α-OH	H	H	H	β-OH	β-OH	β-H	H
406.	α-H	α-OH	H	H	β-OH	=O	β-OH	β-H
407.	α-H	α-H	H	H	β-OH	=O	β-OH	β-OH
408.	α-H	α-H	H	H	β-OAc	β-OAc	β-OH	β-H
409.	α-H	α-H	H	H	β-OH	β-OH	β-OH	β-H
410.	α-OH	α-H	H	H	β-OH	β-OH	β-OH	β-H
411.	α-H	α-H	H	H	β-OH	β-OH	β-OH	β-H
412.	α-H	α-H	H	H	β-OH	=O	α-OH	β-H
418.	H	α-OH	H	H	β-OH	=O	H	H
483.	α-OAc	H	H	H	β-OH	b-OH	β-OH	H
484.	α-OH	H	H	H	β-OH	b-OH	β-OH	H

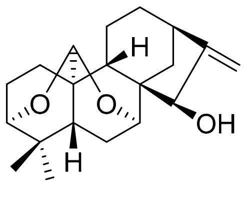

333

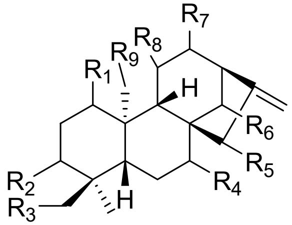

	R1	R_2	R_3	R_4	R_5	R_6	R_7	R_8	R_9
339.	H	H	H	α-OH	=O	β-OH	α-OH	β-OH	OAc
340.	H	H	OH	α-OH	=O	β-OH	α-H	β-H	OAc
341.	H	H	OH	α-OH	=O	β-OH	α-H	β-OH	OAc
342.	H	H	H	α-OH	=O	β-OH	α-OH	β-OH	OH
346.	H	H	H	α-OH	=O	β-OH	α-H	β-OH	OAc
347.	H	H	H	α-OH	=O	β-OH	α-H	β-OH	OH
348.	H	H	H	α-OH	=O	β-OH	α-H	β-OH	H
349.	H	H	H	α-OH	=O	β-OH	α-OH	β-OH	H
357.	H	β-OH	H	α-OH	=O	β-OH	α-OH	H	H
358.	H	α-OH	H	α-OH	=O	β-OH	α-OAc	H	H
359.	H	β-OAc	H	α-OAc	=O	β-OH	=O	H	H
360.	H	β-OH	H	α-OH	β-OH	β-OH	=O	H	H
361.	H	β-OH	H	α-H	β-OH	β-OAc	α-OH	H	H
362.	H	α-OH	H	α-OH	β-OH	β-OH	=O	H	H
363.	α-OH	α-OAc	H	α-OH	=O	β-OH	α-OH	H	H
364.	α-OH	α-OAc	H	α-OH	β-OAc	β-OH	=O	H	H
370.	H	α-OH	H	α-OH	=O	β-OH	a-H	H	H
371.	H	α-OH	H	α-OH	=O	β-OH	α-OH	H	H
372.	H	β-OAc	H	α-OH	=O	β-OH	=O	H	H
373.	α-OH	β-OAc	H	α-OH	=O	β-OH	=O	H	H
374.	H	=O	H	α-OH	=O	β-OH	α-H	H	H
375.	H	=O	H	α-OH	=O	β-OAc	α-H	H	H
376.	α-OH	β-OAc	H	α-OH	=O	β-OH	α-H	H	H
377.	α-OH	β-OH	H	α-OH	=O	β-OH	α-H	H	H
378.	α-OH	H	H	α-OH	=O	β-OH	α-H	H	H
379.	H	β-OH	H	α-OH	=O	β-OH	α-H	H	H
380.	H	β-OAc	H	α-OH	=O	β-OAc	α-H	H	H
381.	α-OH	β-OAc	H	α-OH	=O	β-OH	=O	H	H

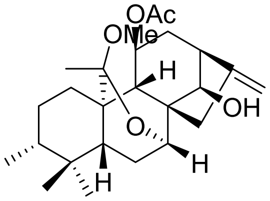

345

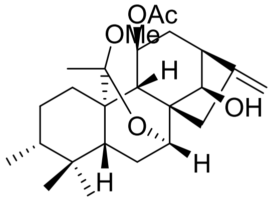

336

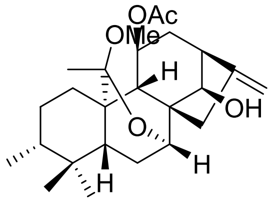

337

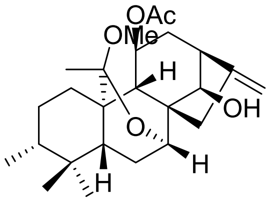

338

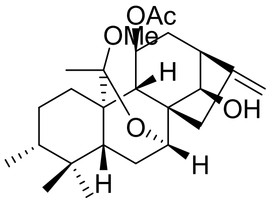

343

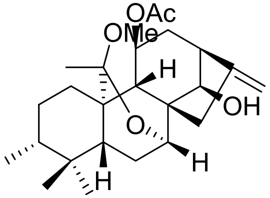

344

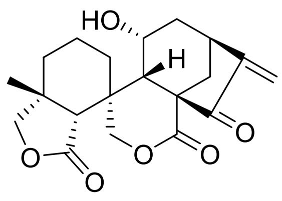

352

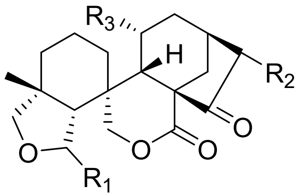

	R1	R2	R3
353.	OMe	α-Me	α-H
354.	α-OH	β-Me	α-H
355.	α-OH	β-Me	α-H

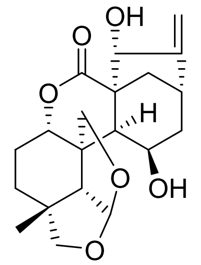

356

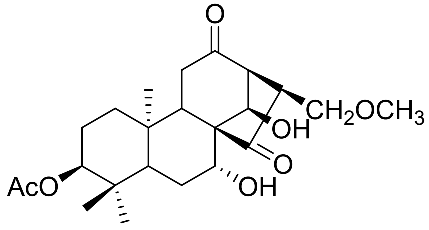

365

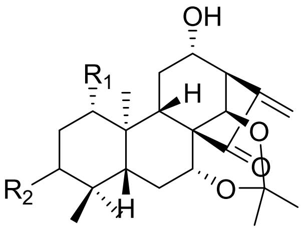

	R_1	R_2
366.	H	α-OH
367.	H	β-OH
368.	OH	β-OAc
369.	OH	α-OH

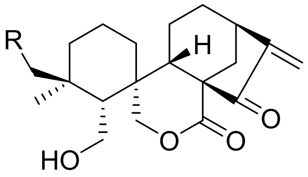

	R
382.	H
399.	OH

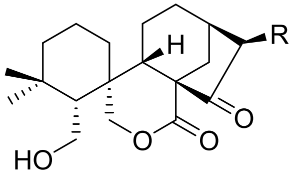

	R
383.	ß-$MeOCH_2$
384.	a-$MeOCH_2$

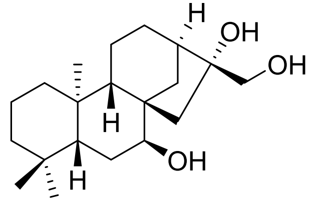

389

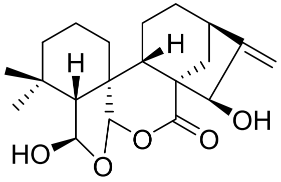

390

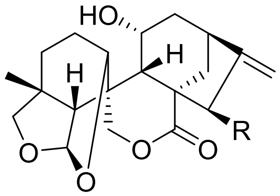

	R
385.	ß-OH
388.	=O

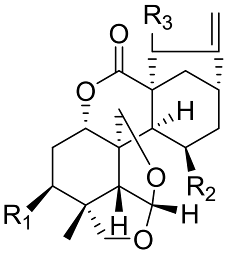

	R_1	R_2	R_3
387.	OH	OH	=O
396.	H	H	=O
397.	H	OH	=O
398.	H	OH	α-OH

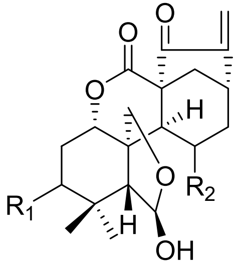

	R_1	R_2
386.	α-OH	β-OH
391.	H	α-OH
392.	β-OH	H
393.	H	H
394.	H	β-OH
395.	β-OH	β-OH

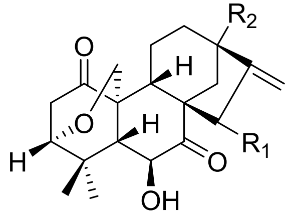

	R_1	R_2
400.	=O	H
402.	OH	a-H
403.	OAc	a-OH
404.	OAc	a-H
421.	b-OH	a-OH
425.	b-OAc	a-OH
427.	b-OAc	H
428.	b-OH	H

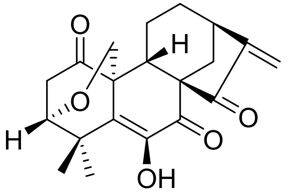

405

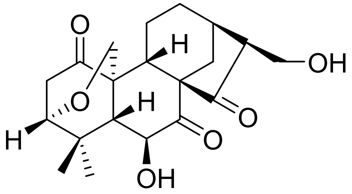

401

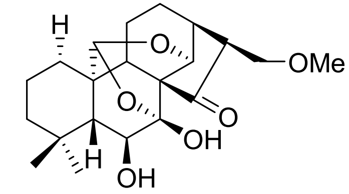

413

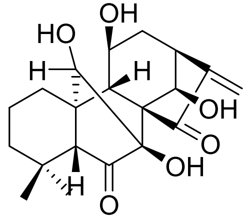

419

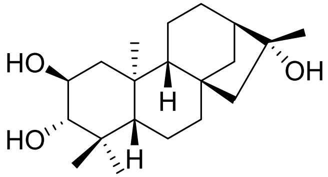

420

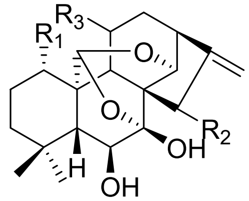

	R_1	R_2	R_3
414.	H	a-OH	α-OH
415.	H	=O	H
416.	OH	=O	H
417.	H	=O	β-OH
441.	OH	β-OH	β-H
442.	OH	β-OH	H
443.	OH	α-OH	H
444.	OH	=O	H
445.	OH	=O	β-H
446.	H	=O	β-OH
447.	H	β-OH	β-H

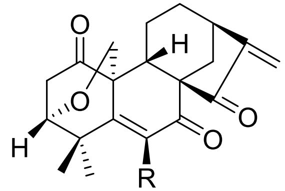

	R
422.	OH
429.	OAc

422

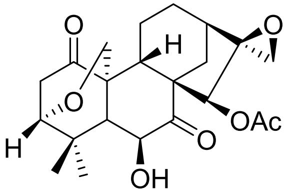

426

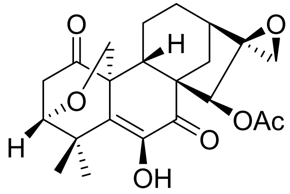

423

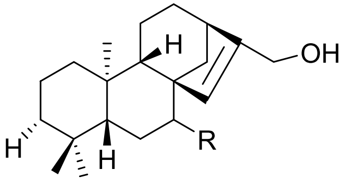

	R
424.	=O
430.	H

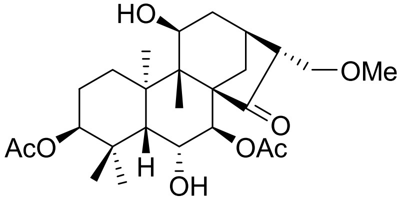

435

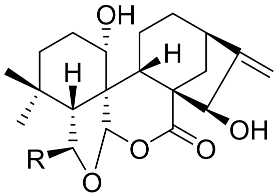

	R
448.	OMe
449.	OH

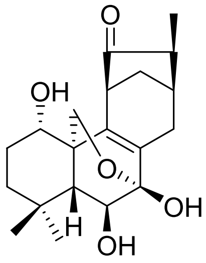

450

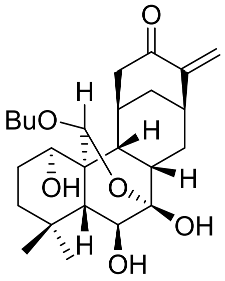

451

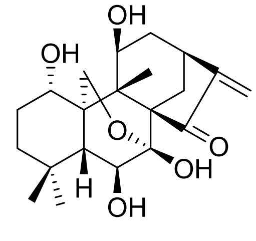

452

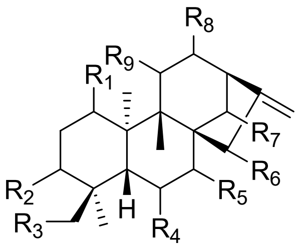

	R1	R_2	R_3	R_4	R_5	R_6	R_7	R_8	R_9
431.	H	β-OH	H	α-OAc	OAc	=O	H	H	β-OH
432.	H	β-OAc	H	α-OAc	OAc	β-OH	H	H	β-OH
433.	H	β-OAc	H	α-OH	OAc	β-OH	H	H	β-OH
434.	H	β-OAc	H	α-OH	OH	=O	H	H	β-OH
436.	H	β-OAc	H	=O	OAc	=O	H	H	β-OH
437.	H	β-OH	H	α-OAc	OH	=O	H	H	β-OH
438.	H	β-OAc	H	α-OAc	OH	=O	H	H	β-OH
439.	H	β-OAc	H	α-OAc	OAc	=O	H	H	β-OH
440.	H	β-OAc	H	α-OH	OAc	=O	H	H	β-OH
453.	H	b-OAc	H	H	α-OH	β-OH	β-OH	H	H
454.	H	b-OH	H	H	α-OH	β-OH	β-OH	H	H
455.	H	α-OH	H	H	α-OH	β-OH	β-OH	H	H
457.	α-H	β-OAc	H	H	α-OH	=O	β-OH	a-OH	H
458.	α-H	β-OH	H	H	α-OH	=O	β-OH	α-H	H
459.	α-OH	β-OH	H	H	α-OH	=O	β-OH	α-H	H
460.	α-H	=O	H	H	α-OH	=O	β-OH	α-H	H
461.	α-H	=O	OH	H	α-OH	=O	β-OH	α-H	H
462.	α-H	β-OAc	H	H	α-OH	=O	β-OH	α-H	H
463.	α-H	β-OH	H	H	α-OH	=O	β-OH	α-H	H
464.	α-H	α-OH	H	H	α-OH	=O	β-OH	α-H	H
465.	α-H	β-OAc	H	H	α-OAc	=O	β-OH	α-H	H
486.	H	H	OH	H	H	β-OH	β-OH	H	β-OH
497.	H	H	H	H	α-OAc	=O	β-OH	α-OH	β-OH

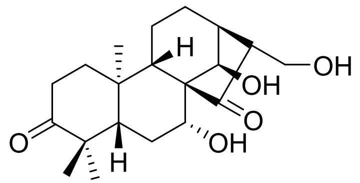

456

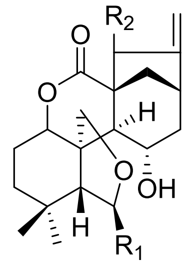

	R_1	R_2
466.	OMe	a-OH
470.	OMe	=O
471.	OH	=O

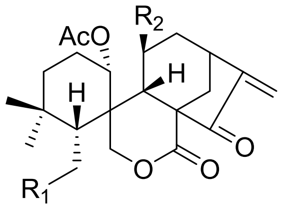

	R_1	R_2
472.	OH	OH
473.	OH	H
474.	OAc	OH
475.	OAc	OAc

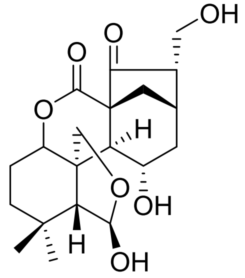

469

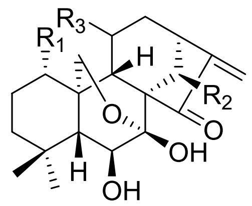

	R_1	R_2	R_3
476.	OAc	OH	H
477.	OH	H	b-OH
478.	OH	OH	H
479.	OH	H	H
480.	OAc	H	a-OAc
481.	H	OH	b-OH

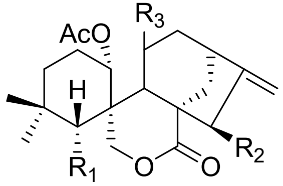

	R_1	R_2	R_3
467.	MeOH	OH	b-OH
468.	MeOAc	OAc	b-OH
482.	CHO	=O	a-OH

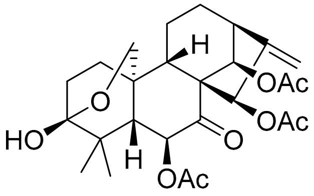

487

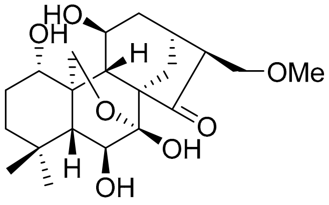

485

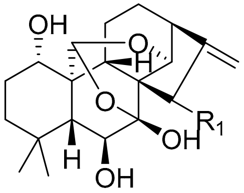

	R
488.	b-OAc
489.	=O

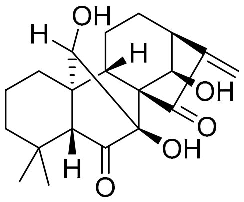

490

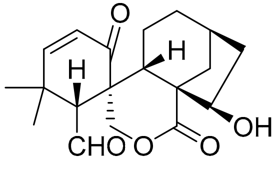

491

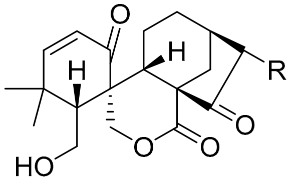

	R
492.	α-Me
493.	β-Me

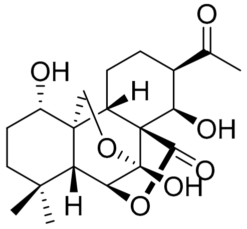

494

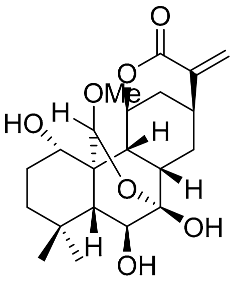

495

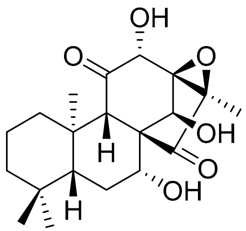

496

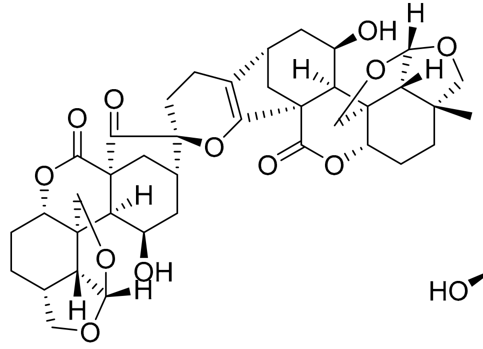

500

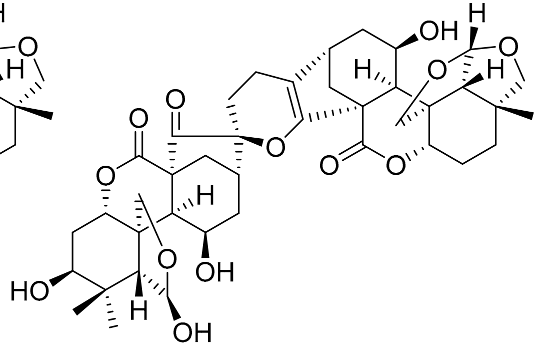

501

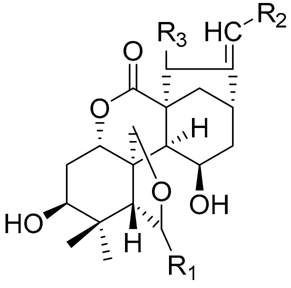

	R_1	R_2	R_3
503.	b-OH	H	a-OH
504.	H	H	=O
505.	H	OH	=O

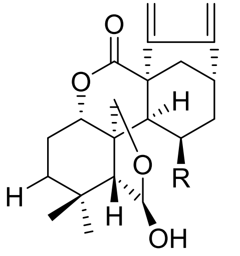

	R
506.	OH
507.	H

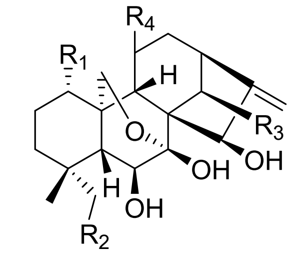

	R_1	R_2	R_3	R_4
508.	H	OH	H	β-OH
509.	OH	OH	H	α-OH
510.	OH	H	OH	H

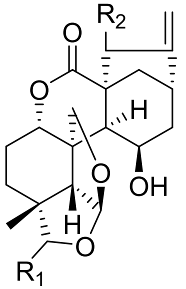

	R_1	R_2
511.	b-OH	=O
512.	a-OH	=O
513.	b-OH	a-OH
514.	a-OH	a-OH
515.	H	=O
516.	H	a-OH

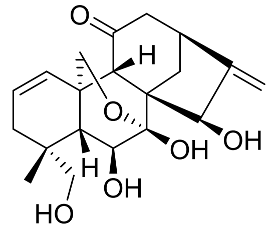

517

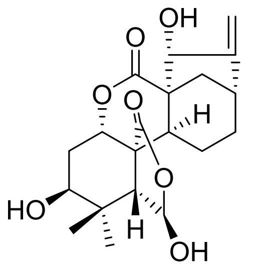

518

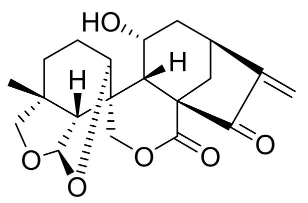

519

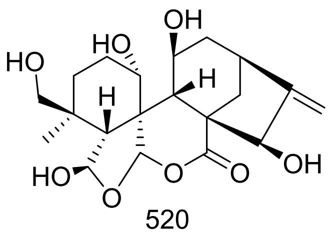

520

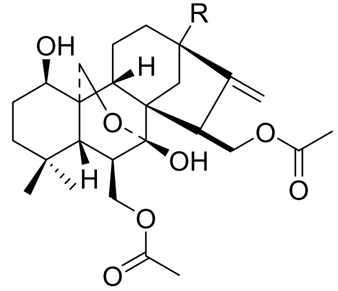

	R
521.	a-OH
522.	H

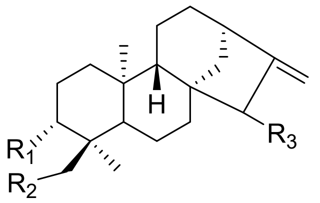

	R_1	R_2	R_3
523.	H	OGlc	
524.	OGlc	H	
525.	H	OGlc	

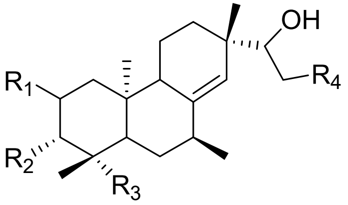

	R_1	R_2	R_3	R_4
526.	H	OH	Me	OH
528.	b-OH	H	COOH	OAc
529.	=O	H	MeOH	OH

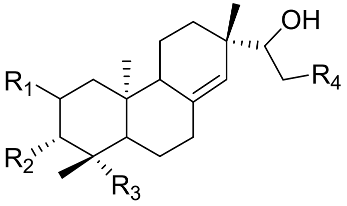

	R_1	R_2	R_3	R_4
530.	OH	H	MeOH	OH
531.	H	OH	Me	OH
532.	H	OH	MeOH	OH
533.	H	OGlc	Me	OH
534.	H	OGlc	Me	OAc

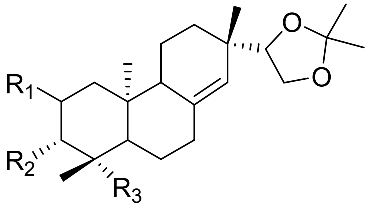

	R_1	R_2	R_3
535.	OH	H	MeOH
536.	H	OH	Me
537.	H	OGlc	Me

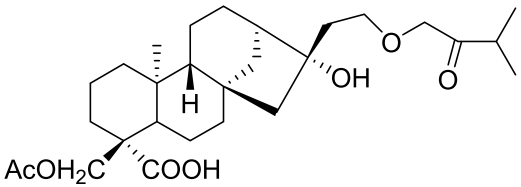

541

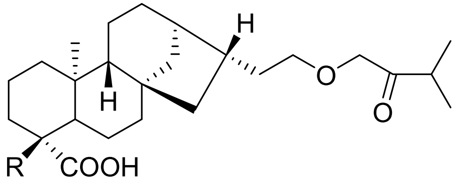

R
540. MeOAc
544. MeOH

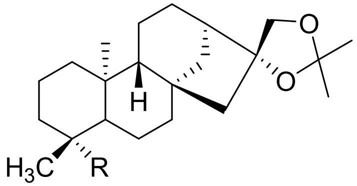

R
549. COOH
550. $COOCH_3$

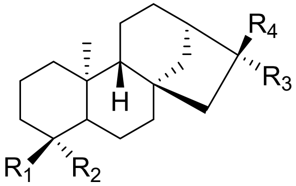

	R_1	R_2	R_3	R_4
538.	MeOAc	COOH	MeOH	H
539.	MeOAc	COOH	OH	MeOH
542	MeOH	COOH	OH	MeOH
543.	MeOH	COOH	MeOH	H
545.	Me	COOH	OH	MeOH
546.	Me	COOH	H	MeOH
547.	Me	$COOCH_3$	H	MeOH
548.	Me	COOH	COOH	H
557.	H	H	OH	Me

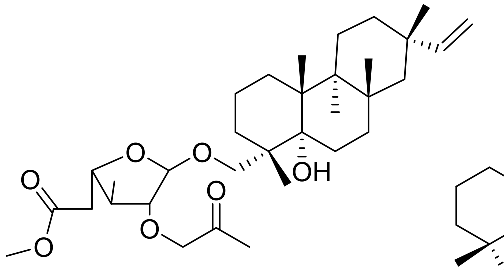

551

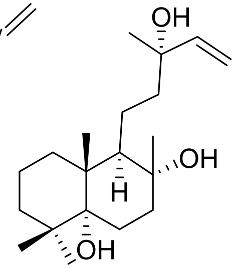

552

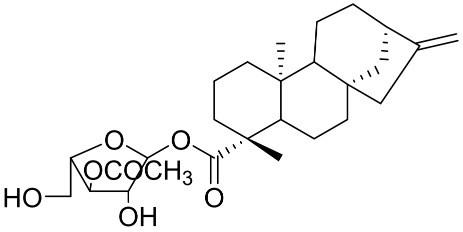

553

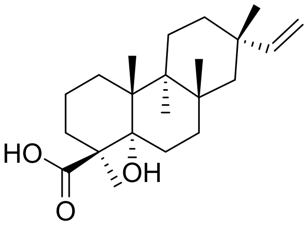

554

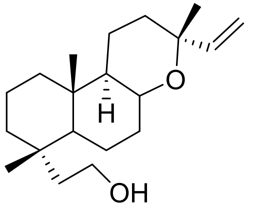

555

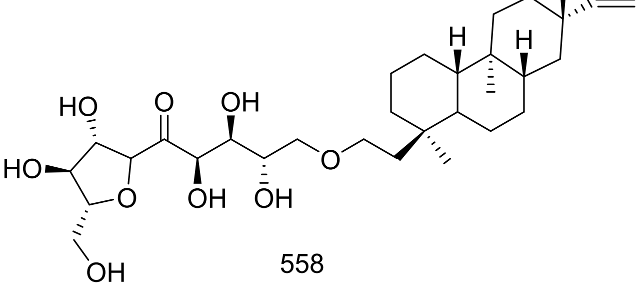

558

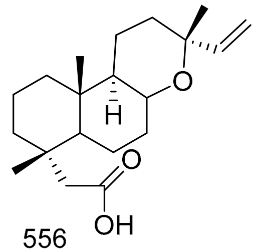

556

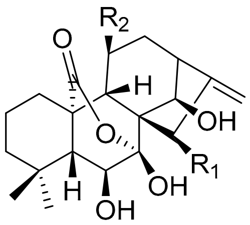

	R_1	R_2
561.	OH	OH
562.	OH	OAc
563.	OH	H
564.	=O	H

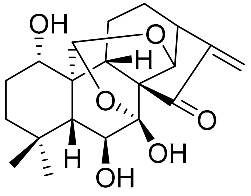

565

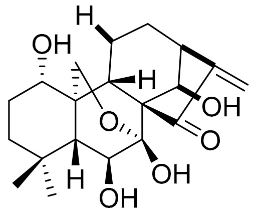

559

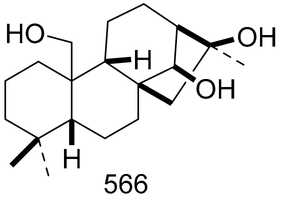

566

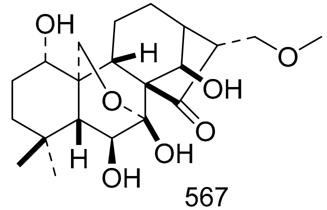

567

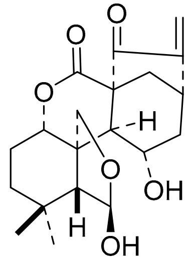

568

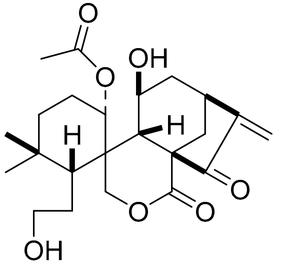

569

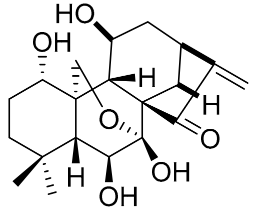

570

SUPPLEMENTARY INFORMATION S2: Source and bioactivities of *ent*-kaurane diterpenoids

Table S1. A table showing the source and reported bioactivities of various *ent*-kaurane diterpenoids.

Structure	Source	Bioactivity
1	*Jungermania tetragona*	Anticancer
2	*Jungermania tetragona*	Anticancer
3	*Jungermania tetragona*	Anticancer
4	*Jungermania tetragona*	NA
5	*Jungermania tetragona*	Anticancer
6	*Jungermania tetragona*	NA
7	*Jungermania tetragona*	NA
8	*Jungermania tetragona*	NA
9	*Jungermania tetragona*	NA
10	*Jungermania tetragona*	Anticancer
11	*Jungermania tetragona*	NA
12	*Jungermania tetragona*	Anticancer
13	*Jungermania tetragona*	NA
14	*Jungermania tetragona*	NA
15	*Jungermania tetragona*	NA
16	*Jungermania tetragona*	NA
17	*Jungermania tetragona*	NA
18	*Jungermania tetragona*	NA
19	*Jungermania tetragona*	NA
20	*Jungermania tetragona*	NA
21	*Jungermania tetragona*	Anticancer
22	*Jungermania tetragona*	Anticancer
23	*Jungermania tetragona*	Anticancer
24	*Jungermania tetragona*	NA
25	*Jungermania tetragona*	NA
26	*Jungermania tetragona*	NA
27	*Jungermania tetragona*	NA
28	*Jungermania tetragona*	NA
29	*Jungermania tetragona*	NA
30	*Jungermania tetragona*	NA
31	*Jungermani. Sp*	Anticancer
32	*Jungermani. Sp*	Anticancer
33	*Jungermani. Sp*	Anticancer
34	*Jungermani. Sp*	Anticancer
35	*Jungermani. Sp*	Anticancer
36	*Jungermani. Sp*	Anticancer
37	*Jungermani. Sp*	Anticancer
38	*Jungermani. Sp*	Anticancer
39	*Jungermani. Sp*	NA

40	*Jungermani. Sp*	NA
41	*Jungermani. Sp*	NA
42	*Jungermani. Sp*	NA
43	*Jungermani. Sp*	NA
44	*Jungermani. Sp*	NA
45	*Jungermani. Sp*	NA
46	*Jungermani. Sp*	NA
47	*Jungermani. Sp*	NA
48	*Jungermani. Sp*	NA
49	*Jungermani. Sp*	NA
50	*Jungermani. Sp*	NA
51	*Jungermani. Sp*	NA
52	*Isodon wikstroemioides*	Anticancer, NO inhibitor
53	*Isodon wikstroemioides*	NA
54	*Isodon wikstroemioides*	Anticancer, NO inhibitor
55	*Isodon wikstroemioides*	Anticancer, NO inhibitor
56	*Isodon wikstroemioides*	Anticancer, NO inhibitor
57	*Isodon wikstroemioides*	NA
58	*Isodon wikstroemioides*	Anticancer, NO inhibitor
59	*Isodon rubescens*	NA
60	*Isodon rubescens*	Anticancer
61	*Isodon rubescens*	NA
62	*Isodon rubescens*	NT
63	*Isodon rubescens*	NA
64	*Isodon rubescens*	NA
65	*Isodon rubescens*	NA
66	*Isodon rubescens*	NA
67	*Isodon rubescens*	Anticancer
68	*Isodon rubescens*	NA
69	*Isodon rubescen var. Taihangensis*	Anticancer
70	*Isodon rubescens*	NA
71	*Isodon eryocalyx*	NT
72	*Isodon eryocalyx*	NT
73	*Isodon eryocalyx*	NT
74	*Isodon eryocalyx*	NT
75	*Isodon eryocalyx*	NT
76	*Jungermania. Sp*	NA
77	*Jungermania. Sp*	NA
78	*Jungermania. Sp*	NA
79	*Jungermania. Sp*	NA
80	*Jungermania. Sp*	NA
81	*Jungermania. Sp*	NA

82	*Jungermania. Sp*	NA
83	*Jungermania. Sp*	NA
84	*Jungermania. Sp*	NA
85	*Jungermania. Sp*	NA
86	*Jungermania. Sp*	NA
87	*Jungermania. Sp*	NA
88	*Jungermania. Sp*	NA
89	*Jungermania. Sp*	NA
90	*Jungermania. Sp*	Anticancer
91	*Jungermania. Sp*	NA
92	*Jungermania. Sp*	NA
93	*Jungermania. Sp*	NA
94	*Jungermania. Sp*	NA
95	*Jungermania. Sp*	NA
96	*Jungermania. Sp*	NA
97	*Pteris multifida*	Anticancer, NO inhibitor
98	*Pteris multifida*	Anticancer
99	*Pteris multifida*	NA
100	*Pteris multifida*	NA
101	*Pteris multifida*	NA
102	*Pteris multifida*	NA
103	*Pteris multifida*	Anticancer, NO inhibitor
104	*Pteris multifida*	NA
105	*Pteris multifida*	NA
106	*Pteris multifida*	NA
107	*Pteris multifida*	NA
108	*Pteris multifida*	NA
109	*Isodon nervosus*	NA
110	*Isodon nervosus*	Anticancer
111	*Isodon nervosus*	Anticancer
112	*Isodon nervosus*	NA
113	*Isodon nervosus*	Anticancer
114	*Isodon nervosus*	NT
115	*Isodon nervosus*	NT
116	*Pteris multifida*	Anticancer
117	*Pteris multifida*	Anticancer
118	*Isodon angustifolius var. Glabrescens*	NT
119	*Isodon angustifolius var. Glabrescens*	NT
120	*Isodon angustifolius var. Glabrescens*	NT
121	*Isodon parvifolius*	NT
122	*Isodon parvifolius*	NT
123	*Isodon parvifolius*	NT

124	*Isodon parvifolius*	NT
125	*Isodon wikstroemioides*	Anticancer, NO inhibitor
126	*Isodon wikstroemioides*	Anticancer, NO inhibitor
127	*Isodon wikstroemioides*	Anticancer, NO inhibitor
128	*Isodon wikstroemioides*	Anticancer, NO inhibitor
129	*Isodon wikstroemioides*	Anticancer
130	*Isodon wikstroemioides*	Anticancer
131	*Isodon wikstroemioides*	Anticancer
132	*Isodon wikstroemioides*	Anticancer
133	*Isodon wikstroemioides*	Anticancer, NO inhibitor
134	*Isodon wikstroemioides*	NT
135	*Isodon wikstroemioides*	Anticancer, NO inhibitor
136	*Isodon wikstroemioides*	Anticancer, NO inhibitor
137	*Isodon wikstroemioides*	Anticancer, NO inhibitor
138	*Isodon wikstroemioides*	Anticancer
139	*Isodon wikstroemioides*	NT
140	*Isodon wikstroemioides*	NA
141	*Isodon wikstroemioides*	Anticancer, NO inhibitor
142	*Isodon wikstroemioides*	Anticancer, NO inhibitor
143	*Isodon wikstroemioides*	Anticancer
144	*Isodon wikstroemioides*	Anticancer
145	*Isodon wikstroemioides*	Anticancer
146	*Isodon wikstroemioides*	Anticancer
147	*Isodon wikstroemioides*	Anticancer, NO inhibitor
148	*Isodon wikstroemioides*	Anticancer, NO inhibitor
149	*Isodon wikstroemioides*	Anticancer, NO inhibitor
150	*Isodon wikstroemioides*	Anticancer, NO inhibitor
151	*Isodon wikstroemioides*	Anticancer, NO inhibitor
152	*Isodon wikstroemioides*	Anticancer, NO inhibitor
153	*Isodon wikstroemioides*	Anticancer, NO inhibitor
154	*Isodon wikstroemioides*	Anticancer, NO inhibitor
155	*Isodon wikstroemioides*	Anticancer
156	*Isodon wikstroemioides*	Anticancer, NO inhibitor
157	*Isodon wikstroemioides*	Anticancer, NO inhibitor
158	*Isodon wikstroemioides*	Anticancer
159	*Isodon wikstroemioides*	Anticancer
160	*Isodon excisoides*	Anticancer
161	*Isodon excisoides*	Anticancer
162	*Isodon excisoides*	Anticancer
163	*Isodon excisoides*	Anticancer
164	*Isodon excisoides*	Anticancer
165	*Isodon excisoides*	Anticancer

166	*Isodon excisoides*	Anticancer
167	*Isodon excisoides*	Anticancer
168	*Isodon excisoides*	Anticancer
169	*Isodon henryi*	Anticancer
170	*Isodon henryi*	Anticancer
171	*Isodon henryi*	Anticancer
172	*Isodon henryi*	Anticancer
173	*Isodon henryi*	Anticancer
174	*Isodon henryi*	Anticancer
175	*Isodon henryi*	Anticancer
176	*Isodon rosthornii*	NA
177	*Isodon rosthornii*	NA
178	*Isodon rosthornii*	NA
179	*Isodon rosthornii*	Anticancer
180	*Isodon rosthornii*	NT
181	*Isodon rosthornii*	Anticancer
182	*Isodon rosthornii*	Anticancer
183	*Isodon rosthornii*	Anticancer
184	*Isodon rosthornii*	Anticancer
185	*Isodon rosthornii*	Anticancer
186	*Isodon rosthornii*	Anticancer
187	*Isodon rosthornii*	Anticancer, NO inhibitor
188	*Isodon rosthornii*	Anticancer
189	*Isodon rosthornii*	NT
190	*Isodon rosthornii*	Anticancer, NO inhibitor
191	*Isodon rosthornii*	NT
192	*Isodon rosthornii*	Anticancer, NO inhibitor
193	*Isodon rosthornii*	Anticancer, NO inhibitor
194	*Isodon rosthornii*	Anticancer, NO inhibitor
195	*Isodon rosthornii*	Anticancer, NO inhibitor
196	*Isodon rosthornii*	Anticancer, NO inhibitor
197	*Isodon rosthornii*	Anticancer, NO inhibitor
198	*Isodon rosthornii*	Anticancer
199	*Isodon rosthornii*	Anticancer, NO inhibitor
200	*Isodon rosthornii*	Anticancer
201	*Isodon rosthornii*	Anticancer, NO inhibitor
202	*Isodon tenuifolius*	Anticancer
203	*Isodon tenuifolius*	Anticancer, NO inhibitor
204	*Isodon tenuifolius*	Anticancer
205	*Isodon tenuifolius*	Anticancer
206	*Isodon tenuifolius*	Anticancer
207	*Isodon tenuifolius*	Anticancer

208	*Isodon tenuifolius*	Anticancer
209	*Isodon tenuifolius*	Anticancer
210	*Isodon tenuifolius*	Anticancer
211	*Isodon tenuifolius*	Anticancer, NO inhibitor
212	*Isodon tenuifolius*	Anticancer, NO inhibitor
213	*Isodon tenuifolius*	NA
214	*Isodon tenuifolius*	Anticancer
215	*Isodon tenuifolius*	Anticancer, NO inhibitor
216	*Isodon tenuifolius*	NA
217	*Isodon tenuifolius*	NA
218	*Isodon tenuifolius*	Anticancer, NO inhibitor
219	*Sideritis congesta*	NO, BchE, AchE inhibitors
220	*Sideritis congesta*	NO, BchE, AchE inhibitors
221	*Sideritis congesta*	NO, BchE, AchE inhibitors
222	*Sideritis congesta*	NO, BchE, AchE inhibitors
223	*Sideritis congesta*	NO, BchE, AchE inhibitors
224	*Sideritis congesta*	NO, BchE, AchE inhibitors
225	*Sideritis congesta*	NO, BchE, AchE inhibitors
226	*Sideritis congesta*	NO, BchE, AchE inhibitors
227	*Sideritis congesta*	NO, BchE, AchE inhibitors
228	*Isodon sinuolata*	Anticancer
229	*Isodon sinuolata*	Anticancer
230	*Isodon sinuolata*	NA
231	*Isodon sinuolata*	NA
232	*Isodon sinuolata*	Anticancer
233	*Isodon sinuolata*	Anticancer
234	*Isodon sinuolata*	NA
235	*Isodon sinuolata*	NA
236	*Isodon sinuolata*	NA
237	*Isodon sinuolata*	Anticancer
238	*Isodon sinuolata*	NA
239	*Isodon sinuolata*	NA
240	*Isodon sinuolata*	NA
241	*Isodon sinuolata*	Anticancer
242	*Isodon sinuolata*	Anticancer
243	*Isodon sinuolata*	Anticancer
244	*Isodon sinuolata*	Anticancer
245	*Isodon sinuolata*	Anticancer
246	*Isodon sinuolata*	Anticancer
247	*Isodon sinuolata*	Anticancer
248	*Isodon sinuolata*	Anticancer
249	*Isodon sinuolata*	Anticancer

250	*Isodon sinuolata*	NT
251	*Isodon sinuolata*	NT
252	*Geopyxis. Sp*	NT
253	*Geopyxis. Sp*	Anticancer
254	*Geopyxis. Sp*	NA
255	*Geopyxis. Sp*	NA
256	*Geopyxis. Sp*	NA
257	*Geopyxis. Sp*	NA
258	*Geopyxis. Sp*	Anticancer
259	*Geopyxis. Sp*	Anticancer
260	*Geopyxis. Sp*	Anticancer
261	*Geopyxis. Sp*	Anticancer
262	*Geopyxis. Sp*	NA
263	*Geopyxis. Sp*	Anticancer
264	*Geopyxis. Sp*	NA
265	*Geopyxis. Sp*	Anticancer
266	*Geopyxis. Sp*	Anticancer
267	*Isodon japonicus*	NO inhibitors
268	*Isodon japonicus*	NO inhibitors
269	*Isodon japonicus*	NO inhibitors
270	*Isodon japonicus*	NO inhibitors
271	*Isodon japonicus*	NO inhibitors
272	*Isodon japonicus*	NO inhibitors
273	*Isodon japonicus*	NO inhibitors
274	*Isodon japonicus*	NO inhibitors
275	*Isodon japonicus*	NO inhibitors
276	*Isodon japonicus*	NO inhibitors
277	*Isodon japonicus*	NO inhibitors
278	*Isodon japonicus*	NO inhibitors
279	*Isodon japonicus*	NO inhibitors
280	*Isodon japonicus*	NO inhibitors
281	*Isodon japonicus*	NO inhibitors
282	*Sideritis arguta*	NA
283	*Sideritis arguta*	NT
284	*Sideritis arguta*	NA
285	*Sideritis arguta*	NA
286	*Sideritis arguta*	Anticancer
287	*Sideritis arguta*	NA
288	*Jungermania atrobrunnea*	NT
289	*Jungermania atrobrunnea*	NT
290	*Jungermania atrobrunnea*	NT
291	*Jungermania atrobrunnea*	NT

292	*Jungermania atrobrunnea*	NT
293	*Jungermania atrobrunnea*	NT
294	*Jungermania atrobrunnea*	NT
295	*Jungermania atrobrunnea*	NT
296	*Jungermania atrobrunnea*	NT
297	*Jungermania atrobrunnea*	NT
298	*Isodon nervosus*	NT
299	*Isodon nervosus*	Anticancer
300	*Isodon nervosus*	NT
301	*Isodon nervosus*	NT
302	*Isodon nervosus*	NT
303	*Isodon nervosus*	NT
304	*Isodon nervosus*	NT
305	*Isodon nervosus*	NT
306	*Isodon nervosus*	NT
307	*Isodon nervosus*	NT
308	*Isodon nervosus*	Anticancer
309	*Isodon nervosus*	NT
310	*Isodon nervosus*	NT
311	*Isodon nervosus*	NT
312	*Isodon nervosus*	NT
313	*Isodon nervosus*	Anticancer
314	*Isodon nervosus*	Anticancer
315	*Isodon nervosus*	NT
316	*Isodon nervosus*	NT
317	*Isodon nervosus*	Anticancer
318	*Isodon albopilosus*	NT
319	*Isodon albopilosus*	NT
320	*Isodon albopilosus*	NT
321	*Isodon albopilosus*	NT
322	*Isodon albopilosus*	NT
323	*Isodon albopilosus*	NT
324	*Isodon albopilosus*	NT
325	*Isodon albopilosus*	NT
326	*Isodon albopilosus*	NT
327	*Isodon albopilosus*	NT
328	*Isodon albopilosus*	NT
329	*Isodon albopilosus*	NT
330	*Isodon albopilosus*	NT
331	*Isodon albopilosus*	NT
332	*Isodon albopilosus*	NT
333	*Isodon rubescens var. lushiensis*	NA

334	*Isodon rubescens var. lushiensis*	NA
335	*Isodon rubescens var. lushiensis*	NA
336	*Isodon rubescens var. lushiensis*	Anticancer
337	*Isodon rubescens var. lushiensis*	NA
338	*Isodon rubescens var. lushiensis*	NA
339	*Isodon rubescens var. lushiensis*	NA
340	*Isodon rubescens var. lushiensis*	Anticancer
341	*Isodon rubescens var. lushiensis*	NA
342	*Isodon rubescens var. lushiensis*	NA
343	*Isodon rubescens var. lushiensis*	Anticancer
344	*Isodon rubescens var. lushiensis*	Anticancer
345	*Isodon rubescens var. lushiensis*	Anticancer
346	*Isodon rubescens var. lushiensis*	Anticancer
347	*Isodon rubescens var. lushiensis*	Anticancer
348	*Isodon rubescens var. lushiensis*	Anticancer
349	*Isodon rubescens var. lushiensis*	Anticancer
350	*Isodon rubescens var. lushiensis*	Anticancer
351	*Isodon rubescens var. lushiensis*	Anticancer
352	*Isodon rubescens var. lushiensis*	Anticancer
353	*Isodon rubescens var. lushiensis*	NA
354	*Isodon rubescens var. lushiensis*	NA
355	*Isodon rubescens var. lushiensis*	NA
356	*Isodon rubescens var. lushiensis*	NA
357	*Isodon pharicus*	NT
358	*Isodon pharicus*	Anticancer
359	*Isodon pharicus*	NA
360	*Isodon pharicus*	NT
361	*Isodon pharicus*	NA
362	*Isodon pharicus*	Anticancer
363	*Isodon pharicus*	Anticancer
364	*Isodon pharicus*	Anticancer
365	*Isodon pharicus*	Anticancer
366	*Isodon pharicus*	Anticancer
367	*Isodon pharicus*	NT
368	*Isodon pharicus*	NT
369	*Isodon pharicus*	NT
370	*Isodon pharicus*	Anticancer
371	*Isodon pharicus*	NA
372	*Isodon pharicus*	Anticancer
373	*Isodon pharicus*	NA
374	*Isodon pharicus*	Anticancer
375	*Isodon pharicus*	Anticancer

376	*Isodon pharicus*	NA
377	*Isodon pharicus*	NA
378	*Isodon pharicus*	Anticancer
379	*Isodon pharicus*	Anticancer
380	*Isodon pharicus*	Anticancer
381	*Isodon pharicus*	NA
382	*Isodon sculponeatus*	Anticancer
383	*Isodon sculponeatus*	Anticancer
384	*Isodon sculponeatus*	NA
385	*Isodon sculponeatus*	NA
386	*Isodon sculponeatus*	NA
387	*Isodon sculponeatus*	NA
388	*Isodon sculponeatus*	Anticancer
389	*Isodon sculponeatus*	NA
390	*Isodon sculponeatus*	NA
391	*Isodon sculponeatus*	Anticancer
392	*Isodon sculponeatus*	Anticancer
393	*Isodon sculponeatus*	Anticancer
394	*Isodon sculponeatus*	Anticancer
395	*Isodon sculponeatus*	Anticancer
396	*Isodon sculponeatus*	Anticancer
397	*Isodon sculponeatus*	Anticancer
398	*Isodon sculponeatus*	NA
399	*Isodon sculponeatus*	NT
400	*Isodon eriocalyx var. laxiflora*	Anticancer
401	*Isodon eriocalyx var. laxiflora*	Anticancer
402	*Isodon eriocalyx var. laxiflora*	Anticancer
403	*Isodon eriocalyx var. laxiflora*	NA
404	*Isodon eriocalyx var. laxiflora*	NT
405	*Isodon eriocalyx var. laxiflora*	Anticancer
406	*Isodon phyllostachys*	NA
407	*Isodon phyllostachys*	NA
408	*Isodon phyllostachys*	NA
409	*Isodon phyllostachys*	NA
410	*Isodon phyllostachys*	NA
411	*Isodon phyllostachys*	NA
412	*Isodon phyllostachys*	NA
413	*Isodon phyllostachys*	NA
414	*Isodon phyllostachys*	NA
415	*Isodon phyllostachys*	NA
416	*Isodon phyllostachys*	NA
417	*Isodon phyllostachys*	NA

418	*Isodon phyllostachys*	NA
419	*Isodon phyllostachys*	NA
420	*Isodon phyllostachys*	NA
421	*Isodon eriocalyx var. laxiflora*	NA
422	*Isodon eriocalyx var. laxiflora*	NA
423	*Isodon eriocalyx var. laxiflora*	NA
424	*Isodon eriocalyx var. laxiflora*	NA
425	*Isodon eriocalyx var. laxiflora*	NA
426	*Isodon eriocalyx var. laxiflora*	NA
427	*Isodon eriocalyx var. laxiflora*	NA
428	*Isodon eriocalyx var. laxiflora*	Anticancer
429	*Isodon eriocalyx var. laxiflora*	NA
577	*Isodon nervosus*	Anticancer
431	*Isodon rubescens*	Anticancer
432	*Isodon rubescens*	NA
433	*Isodon rubescens*	NA
434	*Isodon rubescens*	Anticancer
435	*Isodon rubescens*	NA
436	*Isodon rubescens*	Anticancer
437	*Isodon rubescens*	Anticancer
438	*Isodon rubescens*	Anticancer
439	*Isodon rubescens*	Anticancer
440	*Isodon rubescens*	Anticancer
441	*Isodon rosthorni*	NA
442	*Isodon rosthorni*	NA
443	*Isodon rosthorni*	NA
444	*Isodon rosthorni*	NA
445	*Isodon rosthorni*	Anticancer
446	*Isodon rosthorni*	Anticancer
447	*Isodon rosthorni*	Anticancer
448	*Isodon rosthorni*	NA
449	*Isodon rosthorni*	NA
450	*Isodon parvifolius*	NA
451	*Isodon parvifolius*	Anticancer
452	*Isodon parvifolius*	NT
453	*Isodon henryi*	NT
454	*Isodon henryi*	NT
455	*Isodon henryi*	NT
456	*Isodon henryi*	NT
457	*Isodon henryi*	NT
458	*Isodon henryi*	NT
459	*Isodon henryi*	NT

460	*Isodon henryi*	NT
461	*Isodon henryi*	NT
462	*Isodon henryi*	NT
463	*Isodon henryi*	NT
464	*Isodon henryi*	NT
465	*Isodon henryi*	NT
466	*Isodon japonicus*	NT
467	*Isodon japonicus*	NT
468	*Isodon japonicus*	NT
469	*Isodon japonicus*	NT
470	*Isodon japonicus*	NT
471	*Isodon japonicus*	NT
472	*Isodon japonicus*	NT
473	*Isodon japonicus*	NT
474	*Isodon japonicus*	NT
475	*Isodon japonicus*	NT
476	*Isodon japonicus*	NT
477	*Isodon japonicus*	NT
478	*Isodon japonicus*	NT
479	*Isodon japonicus*	NT
480	*Isodon japonicus*	NT
481	*Isodon japonicus*	NT
482	*Isodon japonicus*	NT
483	*Isodon japonicus*	NT
484	*Isodon japonicus*	NT
485	*Isodon japonicus*	NT
486	*Isodon rubescens*	NT
487	*Isodon rubescens*	NT
488	*Isodon rubescens*	NT
489	*Isodon rubescens*	Anticancer
490	*Isodon rubescens*	Anticancer
491	*Isodon rubescens*	NT
492	*Isodon rubescens*	NT
493	*Isodon rubescens*	NT
494	*Rabdosia rubescens*	NT
495	*Rabdosia rubescens*	NT
496	*Isodon scoparius*	NA
497	*Isodon scoparius*	NA
498	*Isodon scoparius*	Anticancer
499	*Isodon scoparius*	NT
500	*Isodon sculponeatus*	Anticancer
501	*Isodon sculponeatus*	Anticancer

502	*Isodon sculponeatus*	NT
503	*Isodon sculponeatus*	Anticancer
504	*Isodon sculponeatus*	NT
505	*Isodon sculponeatus*	Anticancer
506	*Isodon sculponeatus*	Anticancer
507	*Isodon sculponeatus*	NT
508	*Isodon sculponeatus*	NT
509	*Isodon sculponeatus*	NT
510	*Isodon sculponeatus*	NT
511	*Isodon sculponeatus*	NT
512	*Isodon sculponeatus*	NT
513	*Isodon sculponeatus*	NT
514	*Isodon sculponeatus*	Anticancer
515	*Isodon sculponeatus*	NT
516	*Isodon sculponeatus*	NT
517	*Isodon sculponeatus*	NT
518	*Isodon sculponeatus*	Anticancer
519	*Isodon sculponeatus*	NT
520	*Isodon japonica*	NT
521	*Isodon japonica*	NT
522	*Casearia sylvestris*	NT
523	*Casearia sylvestris*	NT
524	*Casearia sylvestris*	NT
525	*Siegesbeckia pubescens*	NT
526	*Siegesbeckia pubescens*	NT
527	*Siegesbeckia pubescens*	NT
528	*Siegesbeckia pubescens*	NT
529	*Siegesbeckia pubescens*	NT
530	*Siegesbeckia pubescens*	NT
531	*Siegesbeckia pubescens*	NT
532	*Siegesbeckia pubescens*	NT
533	*Siegesbeckia pubescens*	NT
534	*Siegesbeckia pubescens*	NT
535	*Siegesbeckia pubescens*	NT
536	*Siegesbeckia pubescens*	NT
537	*Siegesbeckia pubescens*	NT
538	*Siegesbeckia pubescens*	NT
539	*Siegesbeckia pubescens*	NT
540	*Siegesbeckia pubescens*	NT
541	*Siegesbeckia pubescens*	NT
542	*Siegesbeckia pubescens*	NT
543	*Siegesbeckia pubescens*	NT

544	*Siegesbeckia pubescens*	NT
545	*Siegesbeckia pubescens*	NT
546	*Siegesbeckia pubescens*	NT
547	*Siegesbeckia pubescens*	NT
548	*Siegesbeckia pubescens*	NT
549	*Siegesbeckia pubescens*	NT
550	*Sagittaria trifolia var. sinensis*	NT
551	*Sagittaria trifolia var. sinensis*	NT
552	*Sagittaria trifolia var. sinensis*	NT
553	*Sagittaria trifolia var. sinensis*	NT
554	*Sagittaria trifolia var. sinensis*	NT
555	*Sagittaria trifolia var. sinensis*	NT
556	*Sagittaria trifolia var. sinensis*	NT
557	*Sagittaria trifolia var. sinensis*	NT
558	*Isodon macrophyllus*	NT
559	*Isodon macrophyllus*	NT
560	*Isodon xerophilus*	NT
561	*Isodon xerophilus*	NT
562	*Isodon xerophilus*	NT
563	*Isodon xerophilus*	NT
564	*Isodon xerophilus*	Anticancer
565	*Isodon macrophyllus*	NT
566	*Isodon macrophyllus*	NT
567	*Isodon macrophyllus*	NT
568	*Isodon macrophyllus*	NT
569	*Isodon macrophyllus*	NT
570	*Isodon nervosus*	NT

NA (Not Active) are not active against tested cancer cell lines ($IC_{50} > 50$) and NT (Not Tested) have no reported bioactivities.

SUPPLEMENTARY INFORMATION S3: Bioactivities and molecular descriptors of *ent*-kaurane diterpenoids

Structure	IUPAC/Systematic name	HL-60	SMMC-7	A-549	MCF-7	SW-480	NO produc (BV-2 cell	NO produc (RAW264.	HepG2	A2780	7860	A549	HBE	PC3-3M	DU145	LNCaP	RWPEI	MDA-MB	NCI-H12	SKOV3	LOVO	T24	K562	SH-SY5Y	A172	Saos-2
1	11β -hydroxy-19,20-diacetoxy-*ent* -16-kaurene-15-one	NT	NT	NT	NT	NT	NT	NT	5.5 ± 0.2	18.8 ± 0.1	>20	>20	2.4 ± 0.1	NT	NT	NT	NT	NT	NT	NT	NT	NT	NT	NT	NT	NT
2	11β ,20-dihydroxy-19-acetoxy-*ent* -16-kauren-15-one	NT	NT	NT	NT	NT	NT	NT	6.0 ± 0.3	19.5 ± 2.5	>20	>20	15.0 ± 0.5	NT	NT	NT	NT	NT	NT	NT	NT	NT	NT	NT	NT	NT
3	11β ,20-dihydroxy-*ent* -16-kauren-15-one	NT	NT	NT	NT	NT	NT	NT	4.2 ± 0.1	>20	>20	13.0 ± 1.8	>20	NT	NT	NT	NT	NT	NT	NT	NT	NT	NT	NT	NT	NT
4	16*S* -11β -hydroxy-19-acetoxy-*ent* -kauran-15-one	NT	NT	NT	NT	NT	NT	NT	>20	>20	>20	>20	20.0 ± 0.8	NT	NT	NT	NT	NT	NT	NT	NT	NT	NT	NT	NT	NT
5	16*S* -11β,20-dihydroxy-19-acetoxy-*ent* -kauran-15-one	NT	NT	NT	NT	NT	NT	NT	>20	>20	3.8 ± 0.5	9.8 ± 0.4c	>20c	NT	NT	NT	NT	NT	NT	NT	NT	NT	NT	NT	NT	NT
6	16*R* -11β ,20-dihydroxy-19-acetoxy-*ent* -kauran-15-one	NT	NT	NT	NT	NT	NT	NT	>20	>20	>20	>20	>20	NT	NT	NT	NT	NT	NT	NT	NT	NT	NT	NT	NT	NT
7	16*S* -11β -hydroxy-20-acetoxy-*ent* -kauran-15-one	NT	NT	NT	NT	NT	NT	NT	>20	>20	>20	>20	>20	NT	NT	NT	NT	NT	NT	NT	NT	NT	NT	NT	NT	NT
8	16*R* -11β -hydroxy-20-acetoxy-*ent* -kauran-15-one	NT	NT	NT	NT	NT	NT	NT	>20	>20	>20	>20	>20	NT	NT	NT	NT	NT	NT	NT	NT	NT	NT	NT	NT	NT
9	16*S* -11β -hydroxy-19,20-diacetoxy-*ent* -kauran-15-one	NT	NT	NT	NT	NT	NT	NT	>20	>20	>20	>20	>20	NT	NT	NT	NT	NT	NT	NT	NT	NT	NT	NT	NT	NT
10	16*R* -11β -hydroxy-19,20-diacetoxy-*ent* -kauran-15-one	NT	NT	NT	NT	NT	NT	NT	>20	>20	4.3 ± 0.2	>20	>20	NT	NT	NT	NT	NT	NT	NT	NT	NT	NT	NT	NT	NT
11	16*S* -11β ,20-dihydroxy-*ent* -kauran-15-one	NT	NT	NT	NT	NT	NT	NT	>20	>20	>20	>20	>20	NT	NT	NT	NT	NT	NT	NT	NT	NT	NT	NT	NT	NT
12	16*S* -11β ,19-dihydroxy-20-diacetoxy-*ent* -kauran-15-one	NT	NT	NT	NT	NT	NT	NT	>20	>20	1.5 ± 0.1	>20	>20	NT	NT	NT	NT	NT	NT	NT	NT	NT	NT	NT	NT	NT
13	16*S* -19-acetoxy-11α ,20α -epoxy-*ent* -kauran-15-one	NT	NT	NT	NT	NT	NT	NT	>20	>20	>20	>20	>20	NT	NT	NT	NT	NT	NT	NT	NT	NT	NT	NT	NT	NT
14	16*S* -15β, 20-dihydroxy- 11β ,16-epoxy-*en* t-kauran	NT	NT	NT	NT	NT	NT	NT	>20	>20	>20	>20	>20	NT	NT	NT	NT	NT	NT	NT	NT	NT	NT	NT	NT	NT
15	11β ,15β -dihydroxy-*ent* -16-kaurene	NT	NT	NT	NT	NT	NT	NT	>20	>20	>20	>20	>20	NT	NT	NT	NT	NT	NT	NT	NT	NT	NT	NT	NT	NT
16	11β ,15β -dihydroxy-20-acetoxy-*ent* -16-kaurene	NT	NT	NT	NT	NT	NT	NT	>20	>20	>20	>20	>20	NT	NT	NT	NT	NT	NT	NT	NT	NT	NT	NT	NT	NT
17	11β ,15β -dihydroxy-19,20-diacetoxy-*ent* -16-kaurene	NT	NT	NT	NT	NT	NT	NT	>20	>20	>20	>20	>20	NT	NT	NT	NT	NT	NT	NT	NT	NT	NT	NT	NT	NT
18	6α ,11β ,15β -trihydroxy-*ent* -16-kaurene	NT	NT	NT	NT	NT	NT	NT	>20	>20	>20	>20	>20	NT	NT	NT	NT	NT	NT	NT	NT	NT	NT	NT	NT	NT
19	15β -hydroxy-11α ,20α -epoxy-*ent* -16-kaurene	NT	NT	NT	NT	NT	NT	NT	>20	>20	>20	>20	>20	NT	NT	NT	NT	NT	NT	NT	NT	NT	NT	NT	NT	NT
20	15β -hydroxy-19-acetoxy-11α ,20α -epoxy-*ent* -16-kaurene	NT	NT	NT	NT	NT	NT	NT	>20	>20	>20	>20	>20	NT	NT	NT	NT	NT	NT	NT	NT	NT	NT	NT	NT	NT
21	11β -hydroxy-19-acetoxy-*ent* -16-kauren-15-one	NT	NT	NT	NT	NT	NT	NT	15.5 ± 1.5	2.4 ± 0.5	8.0 ± 0.3	19.0 ± 1.1	8.1 ± 0.1	NT	NT	NT	NT	NT	NT	NT	NT	NT	NT	NT	NT	NT
22	11β -hydroxy-20-acetoxy-*ent* -16-kauren-15-one	NT	NT	NT	NT	NT	NT	NT	8.0 ± 0.3	2.6 ± 0.1	8.8 ± 0.9	4.0 ± 0.1	9.6 ± 0.3	NT	NT	NT	NT	NT	NT	NT	NT	NT	NT	NT	NT	NT
23	11β -hydroxy-*ent* -16-kauren-15-one	NT	NT	NT	NT	NT	NT	NT	4.2 ± 0.1	0.9 ± 0.1	4.9 ± 0.4	3.8 ± 0.2	5.1 ± 0.8	NT	NT	NT	NT	NT	NT	NT	NT	NT	NT	NT	NT	NT
24	16*S* -11β -hydroxy-*ent* -kauran-15-one	NT	NT	NT	NT	NT	NT	NT	>20	>20	>20	>20	>20	NT	NT	NT	NT	NT	NT	NT	NT	NT	NT	NT	NT	NT
25	16*R* -11β -hydroxy-19-acetoxy-*ent* -kauran-15-one	NT	NT	NT	NT	NT	NT	NT	>20	>20	>20	>20	>20	NT	NT	NT	NT	NT	NT	NT	NT	NT	NT	NT	NT	NT
26	16*R* -11β -hydroxy-*ent* -kauran-15-one	NT	NT	NT	NT	NT	NT	NT	>20	>20	>20	>20	>20	NT	NT	NT	NT	NT	NT	NT	NT	NT	NT	NT	NT	NT
27	16*R* -3β ,11β -dihydroxy-*ent* -kauran-15-one	NT	NT	NT	NT	NT	NT	NT	>20	>20	>20	>20	>20	NT	NT	NT	NT	NT	NT	NT	NT	NT	NT	NT	NT	NT
28	6α ,15β -dihydroxy-*ent* -16-kaurene	NT	NT	NT	NT	NT	NT	NT	>20	>20	>20	>20	>20	NT	NT	NT	NT	NT	NT	NT	NT	NT	NT	NT	NT	NT
29	1α ,15β-dihydroxy-*ent* -16-kaurene	NT	NT	NT	NT	NT	NT	NT	>20	>20	>20	>20	>20	NT	NT	NT	NT	NT	NT	NT	NT	NT	NT	NT	NT	NT
30	14β ,15β -dihydroxy-*ent* -16-kaurene	NT	NT	NT	NT	NT	NT	NT	>20	>20	>20	>20	>20	NT	NT	NT	NT	NT	NT	NT	NT	NT	NT	NT	NT	NT
31	13,18-dihydroxy-*ent* -9(11),16-kauradien-15-one	3.7±0.2	NT	NT	NT	NT	NT	NT	NT	NT	NT	NT	NT	5.5±0.1	6.3±0.5	11.6±1.0	11.7±1.3	12.3±1.1	10.7±0.8	8.6±0.4	9.0±0.6	10.4±1.0	10.2±0.1	4.4±0.1	4.4±0.3	7.8±0.2
32	13-hydroxy-*ent* -9(11),16-kauradien-18-al-15-one	4.4±0.7	NT	NT	NT	NT	NT	NT	NT	NT	NT	NT	NT	11.4±1.0	11.6±0.7	9.5±0.4	12.7±0.5	21.6±0.7	12.2±0.7	7.4±0.7	9.3±0.9	5.9±0.7	6.3±0.7	4.2±0.5	4.4±0.5	7.3±0.6
33	13,19-dihydroxy-*ent* -9(11),16-kauradien-15-one	6.7±0.5	NT	NT	NT	NT	NT	NT	NT	NT	NT	NT	NT	6.6±0.7	8.3±0.3	12.8±0.2	13.2±0.3	15.5±0.9	19.0±1.1	11.3±0.5	18.4±1.1	13.2±0.2	10.4±1.1	4.9±0.2	13.0±0.9	15.8±0.9
34	18-hydroxy-*ent* -9(11),16-kauradien-15-one	4.7±0.2	NT	NT	NT	NT	NT	NT	NT	NT	NT	NT	NT	21.5±1.2	31±2.2	16.3±1.0	39.3±2.0	32.0±1.8	14.5±0.5	22.3±1.9	11.2±0.9	17.7±1.4	13.8±0.5	9.8±0.4	20.3±1.0	14.0±1.2
35	13-hydroxy-*ent* -9(11),16-kauradien-15-one	1.0±0.1	NT	NT	NT	NT	NT	NT	NT	NT	NT	NT	NT	3.2±0.2	5.5±0.3	3.2±0.2	6.2±0.1	4.2±0.5	2.4±0.1	1.8±0.2	1.8±0.1	1.7±0.2	1.9±0.1	1.2±0.1	3.8±0.3	1.3±0.1
36	13-hydroxyjungermannenone B	1.9±0.1	NT	NT	NT	NT	NT	NT	NT	NT	NT	NT	NT	2.2±0.1	3.2±0.2	2.8±0.1	6.3±0.1	3.4±0.3	2.6±0.1	2.9±0.1	2.3±0.1	2.3±0.1	2.2±0.1	1.6±0.1	2.7±0.1	2.4±0.1
37	13,18-dihydroxyjungermannenone B	NT	NT	NT	NT	NT	NT	NT	NT	NT	NT	NT	NT	8.2±0.3	9.4±1.0	NT	18.6±0.1	10.4±0.4	NT	NT	NT	NT	NT	NT	NT	NT
38	18-hydroxyjungermannenone B	23.6±2.4	NT	NT	NT	NT	NT	NT	NT	NT	NT	NT	NT	> 50	> 50	> 50	> 50	> 50	> 50	2.6±0.2	35.2±2.7	49.1±3.5	41.7±2.8	33.8±2.9	NA	35.5±2.0
39	16α ,17-dihydro-13,18-dihydroxyjungermannenone B	> 50	NT	NT	NT	NT	NT	NT	NT	NT	NT	NT	NT	> 50	> 50	> 50	> 50	> 50	> 50	> 50	> 50	> 50	> 50	> 50	> 50	> 50
40	16α ,17-dihydro-13- hydroxyjungermannenone B	> 50	NT	NT	NT	NT	NT	NT	NT	NT	NT	NT	NT	> 50	> 50	> 50	> 50	> 50	> 50	> 50	> 50	> 50	> 50	> 50	> 50	> 50
41	16α ,17-dihydro-18-aldehy-13-hydroxyjungermannenone B	> 50	NT	NT	NT	NT	NT	NT	NT	NT	NT	NT	NT	> 50	> 50	> 50	> 50	> 50	> 50	> 50	> 50	> 50	> 50	> 50	> 50	> 50
42	16α ,17-dihydro-13,19-dihydroxyjungermannenone B	> 50	NT	NT	NT	NT	NT	NT	NT	NT	NT	NT	NT	> 50	> 50	> 50	> 50	> 50	> 50	> 50	> 50	> 50	> 50	> 50	> 50	> 50
43	6α ,15β -dihydroxy-ent-16-kauren-11-one	> 50	NT	NT	NT	NT	NT	NT	NT	NT	NT	NT	NT	> 50	> 50	> 50	> 50	> 50	> 50	> 50	> 50	> 50	> 50	> 50	> 50	> 50
44	6α ,7α ,15β -trihydroxy-*ent* -16-kauren-11-one	> 50	NT	NT	NT	NT	NT	NT	NT	NT	NT	NT	NT	> 50	> 50	> 50	> 50	> 50	> 50	> 50	> 50	> 50	> 50	> 50	> 50	> 50
45	7α ,15β -dihydroxy-*ent* -16-kauren-11-one	> 50	NT	NT	NT	NT	NT	NT	NT	NT	NT	NT	NT	> 50	> 50	> 50	> 50	> 50	> 50	> 50	> 50	> 50	> 50	> 50	> 50	> 50
46	15β -hydroxy-*ent* -16-kauren-11-one	> 50	NT	NT	NT	NT	NT	NT	NT	NT	NT	NT	NT	> 50	> 50	> 50	> 50	> 50	> 50	> 50	> 50	> 50	> 50	> 50	> 50	> 50
47	16*R* -13-hydroxy-*ent* -9(11)-kauren-15-one	> 50	NT	NT	NT	NT	NT	NT	NT	NT	NT	NT	NT	> 50	> 50	> 50	> 50	> 50	> 50	> 50	> 50	> 50	> 50	> 50	> 50	> 50
48	16*R* -13,18-dihydroxy-*ent* -9(11)-kauren-15-one	> 50	NT	NT	NT	NT	NT	NT	NT	NT	NT	NT	NT	> 50	> 50	> 50	> 50	> 50	> 50	> 50	> 50	> 50	> 50	> 50	> 50	> 50
49	16*R* -13-hydroxy-*ent* -9(11)-kauren-18-al-15-one	> 50	NT	NT	NT	NT	NT	NT	NT	NT	NT	NT	NT	> 50	> 50	> 50	> 50	> 50	> 50	> 50	> 50	> 50	> 50	> 50	> 50	> 50
50	16*R* -13,19-dihydroxy-*ent* -9(11)-kauren-15-one	> 50	NT	NT	NT	NT	NT	NT	NT	NT	NT	NT	NT	> 50	> 50	> 50	> 50	> 50	> 50	> 50	> 50	> 50	> 50	> 50	> 50	> 50
51	16*R* -6α ,13,18-trihydroxy-*ent* -9(11)-kauren-15-one	> 50	NT	NT	NT	NT	NT	NT	NT	NT	NT	NT	NT	> 50	> 50	> 50	> 50	> 50	> 50	> 50	> 50	> 50	> 50	> 50	> 50	> 50
52	7α ,14β -dihydroxy-11β -methoxy-3β -acetoxy-11,20-epoxy-entkaur-16-en-15-one	1.48 ± 0.3	2.93 ± 0.0	2.16 ± 0.1	2.22 ± 0.1	1.14 ± 0.0	NT	2.05 ± 0.21	NT	NT	NT	NT	NT	NT	NT	NT	NT	NT	NT	NT	NT	NT	NT	NT	NT	NT
53	Isowikstroemin I	> 40	> 40	> 40	> 40	> 40	NT	NT	NT	NT	NT	NT	NT	NT	NT	NT	NT	NT	NT	NT	NT	NT	NT	NT	NT	NT
54	Isowikstroemin J	0.84 ± 0.0	2.21 ± 0.0	1.27 ± 0.0	1.25 ± 0.0	1.21 ± 0.0	NT	1.09 ± 0.06	NT	NT	NT	NT	NT	NT	NT	NT	NT	NT	NT	NT	NT	NT	NT	NT	NT	NT
55	Isowikstroemin K	12.93 ± 0.	9.88 ± 0.5	10.94 ± 0.	8.78 ± 0.5	2.87 ± 0.0	NT	8.32 ± 0.19	NT	NT	NT	NT	NT	NT	NT	NT	NT	NT	NT	NT	NT	NT	NT	NT	NT	NT
56	isowikstroemin L	12.69 ± 0.	15.81 ± 0.	28.13 ± 3.	13.50 ± 0.	4.23 ± 0.0	NT	20.91 ± 2.98	NT	NT	NT	NT	NT	NT	NT	NT	NT	NT	NT	NT	NT	NT	NT	NT	NT	NT
57	Isowikstroemin M	> 40	> 40	> 40	> 40	> 40	NT	NT	NT	NT	NT	NT	NT	NT	NT	NT	NT	NT	NT	NT	NT	NT	NT	NT	NT	NT
58	Macrocalyxin B	1.13 ± 0.3	3.23 ± 0.1	4.09 ± 0.3	2.51 ± 0.0	1.72 ± 0.0	NT	1.85 ± 0.2	NT	NT	NT	NT	NT	NT	NT	NT	NT	NT	NT	NT	NT	NT	NT	NT	NT	NT
59	Pseudoirroratin A	NT	NT	NT	NT	NT	NT	NT	NT	NT	NT	NT	NT	NT	NT	NT	NT	NT	NT	NT	NT	NT	NT	NT	NT	NT
60	Jianshirubesin D	> 40	> 40	30.11	16.22	22.62	NT	NT	NT	NT	NT	NT	NT	NT	NT	NT	NT	NT	NT	NT	NT	NT	NT	NT	NT	NT
61	Jianshirubesin E	> 40	> 40	> 40	> 40	> 40	NT	NT	NT	NT	NT	NT	NT	NT	NT	NT	NT	NT	NT	NT	NT	NT	NT	NT	NT	NT
62	Jianshirubesin F	NT	NT	NT	NT	NT	NT	NT	NT	NT	NT	NT	NT	NT	NT	NT	NT	NT	NT	NT	NT	NT	NT	NT	NT	NT
63	Rubescensin W	> 40	> 40	> 40	> 40	> 40	NT	NT	NT	NT	NT	NT	NT	NT	NT	NT	NT	NT	NT	NT	NT	NT	NT	NT	NT	NT
64	Kaurine A	>40	>40	>40	>40	>40	NT	NT	NT	NT	NT	NT	NT	NT	NT	NT	NT	NT	NT	NT	NT	NT	NT	NT	NT	NT
65	Kaurine B	>40	>40	>40	>40	>40	NT	NT	NT	NT	NT	NT	NT	NT	NT	NT	NT	NT	NT	NT	NT	NT	NT	NT	NT	NT
66	Kaurine C	>40	>40	>40	>40	>40	NT	NT	NT	NT	NT	NT	NT	NT	NT	NT	NT	NT	NT	NT	NT	NT	NT	NT	NT	NT
67	Oridonin	7.2 - 18.9	7.2 - 18.9	7.2 - 18.9	7.2 - 18.9	7.2 - 18.9	NT	NT	NT	NT	NT	NT	NT	NT	NT	NT	NT	NT	NT	NT	NT	NT	NT	NT	NT	NT
68	Hebeirubescensin	>40	>40	>40	>40	>40	NT	NT	NT	NT	NT	NT	NT	NT	NT	NT	NT	NT	NT	NT	NT	NT	NT	NT	NT	NT
69	Rubescensin S	NT	NT	NT	NT	NT	NT	NT	NT	NT	NT	NT	NT	NT	NT	NT	NT	NT	NT	NT	NT	NT	7.03	NT	NT	NT
70	Rubescensin T	>40	>40	>40	>40	>40	NT	NT	NT	NT	NT	NT	NT	NT	NT	NT	NT	NT	NT	NT	NT	NT	NT	NT	NT	NT
71	6β ,7β -dihydroxy-17- (butyl 1',3'-dione)- 7α ,20-epoxy-*ent* -kaur-2-en-1,15-dione	NT	NT	NT	NT	NT	NT	NT	NT	NT	NT	NT	NT	NT	NT	NT	NT	NT	NT	NT	NT	NT	NT	NT	NT	NT
72	7β ,13α -dihydroxy-6β ,15β diacetoxy-7,20-epoxy-*ent* -kaur-16-en-1-one	NT	NT	NT	NT	NT	NT	NT	NT	NT	NT	NT	NT	NT	NT	NT	NT	NT	NT	NT	NT	NT	NT	NT	NT	NT
73	7,20-epoxy-7β ,13β dihydroxy-1-oxo-15,16-seco-16,17-dinor-*ent* -kaur-2-en-6β ,15-	NT	NT	NT	NT	NT	NT	NT	NT	NT	NT	NT	NT	NT	NT	NT	NT	NT	NT	NT	NT	NT	NT	NT	NT	NT
74	3,16,18-trihydroxy-3,4-seco-ent-abieta-4(19),7(8),15- triene	NT	NT	NT	NT	NT	NT	NT	NT	NT	NT	NT	NT	NT	NT	NT	NT	NT	NT	NT	NT	NT	NT	NT	NT	NT
75	laxiflorin O	NT	NT	NT	NT	NT	NT	NT	NT	NT	NT	NT	NT	NT	NT	NT	NT	NT	NT	NT	NT	NT	NT	NT	NT	NT
76	7α ,15α -dihydroxy-*ent* -16-kaurene	>50	NT	NT	NT	NT	NT	NT	NT	NT	NT	NT	NT	> 50	> 50	> 50	> 50	> 50	> 50	> 50	> 50	> 50	> 50	> 50	> 50	> 50
77	6α,15β-dihydroxy-ent-16-kaurene	>50	NT	NT	NT	NT	NT	NT	NT	NT	NT	NT	NT	> 50	> 50	> 50	> 50	> 50	> 50	> 50	> 50	> 50	> 50	> 50	> 50	> 50
78	15β-hydroxy-ent-16-kaurene	>50	NT	NT	NT	NT	NT	NT	NT	NT	NT	NT	NT	> 50	> 50	> 50	> 50	> 50	> 50	> 50	> 50	> 50	> 50	> 50	> 50	> 50
79	16S-13,18-hydroxy-ent-9(11)-kauren-15-one	>50	NT	NT	NT	NT	NT	NT	NT	NT	NT	NT	NT	> 50	> 50	> 50	> 50	> 50	> 50	> 50	> 50	> 50	> 50	> 50	> 50	> 50
80	1α,15β-dihydroxy-ent-16-kaurene	>50	NT	NT	NT	NT	NT	NT	NT	NT	NT	NT	NT	> 50	> 50	> 50	> 50	> 50	> 50	> 50	> 50	> 50	> 50	> 50	> 50	> 50
81	1α,15α-dihydroxy-ent-16-kaurene	>50	NT	NT	NT	NT	NT	NT	NT	NT	NT	NT	NT	> 50	> 50	> 50	> 50	> 50	> 50	> 50	> 50	> 50	> 50	> 50	> 50	> 50
82	1α,7α-dihydroxy-ent-16-kaurene	>50	NT	NT	NT	NT	NT	NT	NT	NT	NT	NT	NT	> 50	> 50	> 50	> 50	> 50	> 50	> 50	> 50	> 50	> 50	> 50	> 50	> 50
83	7α-acetoxy-1α-hydroxy-ent-16-kaurene	>50	NT	NT	NT	NT	NT	NT	NT	NT	NT	NT	NT	> 50	> 50	> 50	> 50	> 50	> 50	> 50	> 50	> 50	> 50	> 50	> 50	> 50
84	9β,15β-dihydroxy-ent-16-kaurene	>50	NT	NT	NT	NT	NT	NT	NT	NT	NT	NT	NT	> 50	> 50	> 50	> 50	> 50	> 50	> 50	> 50	> 50	> 50	> 50	> 50	> 50
85	11α,15β-dihydroxy-ent-16-kaurene	>50	NT	NT	NT	NT	NT	NT	NT	NT	NT	NT	NT	> 50	> 50	> 50	> 50	> 50	> 50	> 50	> 50	> 50	> 50	> 50	> 50	> 50
86	15β,18-dihydroxy-ent-16-kaurene	>50	NT	NT	NT	NT	NT	NT	NT	NT	NT	NT	NT	> 50	> 50	> 50	> 50	> 50	> 50	> 50	> 50	> 50	> 50	> 50	> 50	> 50
87	16R-3α,11β-dihydroxy-ent-9(11)-kauren-15-one	>50	NT	NT	NT	NT	NT	NT	NT	NT	NT	NT	NT	> 50	> 50	> 50	> 50	> 50	> 50	> 50	> 50	> 50	> 50	> 50	> 50	> 50
88	16S-3α,11β-dihydroxy-ent-9(11)-kauren-15-one	>50	NT	NT	NT	NT	NT	NT	NT	NT	NT	NT	NT	> 50	> 50	> 50	> 50	> 50	> 50	> 50	> 50	> 50	> 50	> 50	> 50	> 50
89	16S-11β-hydroxy-ent-kauran-15-one	>50	NT	NT	NT	NT	NT	NT	NT	NT	NT	NT	NT	> 50	> 50	> 50	> 50	> 50	> 50	> 50	> 50	> 50	> 50	> 50	> 50	> 50
90	11β-hydroxy-ent-16-kauren-15-one	0.9±0.1	NT	NT	NT	NT	NT	NT	NT	NT	NT	NT	NT	1.9±0.2	4.5±0.1	2.5±0.2	5.2±0.1	4.8±0.4	2.6±0.2	32.8±2.9	1.4±0.1	2.1±0.1	1.5±0.1	0.7±0.1	3.2±0.2	1.4±0.1
91	11β,15β-dihydroxy-ent-16-kaurene	> 50	NT	NT	NT	NT	NT	NT	NT	NT	NT	NT	NT	> 50	> 50	> 50	> 50	> 50	> 50	> 50	> 50	> 50	> 50	> 50	> 50	> 50
92	15β-acetoxy-ent-kaur-16-en-18-oic acid	> 50	NT	NT	NT	NT	NT	NT	NT	NT	NT	NT	NT	> 50	> 50	> 50	> 50	> 50	> 50	> 50	> 50	> 50	> 50	> 50	> 50	> 50
93	ent-kaur-16-en-18-oic acid	> 50	NT	NT	NT	NT	NT	NT	NT	NT	NT	NT	NT	> 50	> 50	> 50	> 50	> 50	> 50	> 50	> 50	> 50	> 50	> 50	> 50	> 50
94	1α,7α-dihydroxy-ent-15-kaurene	> 50	NT	NT	NT	NT	NT	NT	NT	NT	NT	NT	NT	> 50	> 50	> 50	> 50	> 50	> 50	> 50	> 50	> 50	> 50	> 50	> 50	> 50
95	7α,15α-dihydroxy-ent-16-kaurene	> 50	NT	NT	NT	NT	NT	NT	NT	NT	NT	NT	NT	> 50	> 50	> 50	> 50	> 50	> 50	> 50	> 50	> 50	> 50	> 50	> 50	> 50
96	7α-hydroxy-ent-16-kaurene	> 50	NT	NT	NT	NT	NT	NT	NT	NT	NT	NT	NT	> 50	> 50	> 50	> 50	> 50	> 50	> 50	> 50	> 50	> 50	> 50	> 50	> 50
97	2β,15α-dihydroxy-ent-kaur-16-ene	NT	NT	NT	NT	NT	13.9	NT	NT	NT	NT	NT	NT	NT	NT	NT	NT	NT	NT	NT	NT	NT	NT	NT	NT	NT
98	Creticoside A	NT	NT	NT	NT	NT	92	NT	NT	NT	NT	NT	NT	NT	NT	NT	NT	NT	NT	NT	NT	NT	NT	NT	NT	NT
99	2, 15R,18-trihydroxy-ent-kaur-16-ene	NT	NT	NT	NT	NT	840	NT	NT	NT	NT	NT	NT	NT	NT	NT	NT	NT	NT	NT	NT	NT	NT	NT	NT	NT
100	Pterokaurane M1 2-O-β-d-glucopyranoside	NT	NT	NT	NT	NT	148.1	NT	NT	NT	NT	NT	NT	NT	NT	NT	NT	NT	NT	NT	NT	NT	NT	NT	NT	NT

Structure	LPS activ	CNE2	PC-9/ZD	HeLa	HCT116	NCI 1650	BGC 823	NCI-H46	SF-268	MDA-MB	NB4	PANC-1	SK-BR-3	SHSY5Y	B-carotene-linoleic acid assay	O2- assay	DPPH assa	AChE assa	BChE assay
1	NT	NT	NT	NT	NT	NT	NT	NT	NT	NT	NT	NT	NT	NT	NT	NT	NT	NT	NT
2	NT	NT	NT	NT	NT	NT	NT	NT	NT	NT	NT	NT	NT	NT	NT	NT	NT	NT	NT
3	NT	NT	NT	NT	NT	NT	NT	NT	NT	NT	NT	NT	NT	NT	NT	NT	NT	NT	NT
4	NT	NT	NT	NT	NT	NT	NT	NT	NT	NT	NT	NT	NT	NT	NT	NT	NT	NT	NT
5	NT	NT	NT	NT	NT	NT	NT	NT	NT	NT	NT	NT	NT	NT	NT	NT	NT	NT	NT
6	NT	NT	NT	NT	NT	NT	NT	NT	NT	NT	NT	NT	NT	NT	NT	NT	NT	NT	NT
7	NT	NT	NT	NT	NT	NT	NT	NT	NT	NT	NT	NT	NT	NT	NT	NT	NT	NT	NT
8	NT	NT	NT	NT	NT	NT	NT	NT	NT	NT	NT	NT	NT	NT	NT	NT	NT	NT	NT
9	NT	NT	NT	NT	NT	NT	NT	NT	NT	NT	NT	NT	NT	NT	NT	NT	NT	NT	NT
10	NT	NT	NT	NT	NT	NT	NT	NT	NT	NT	NT	NT	NT	NT	NT	NT	NT	NT	NT
11	NT	NT	NT	NT	NT	NT	NT	NT	NT	NT	NT	NT	NT	NT	NT	NT	NT	NT	NT
12	NT	NT	NT	NT	NT	NT	NT	NT	NT	NT	NT	NT	NT	NT	NT	NT	NT	NT	NT
13	NT	NT	NT	NT	NT	NT	NT	NT	NT	NT	NT	NT	NT	NT	NT	NT	NT	NT	NT
14	NT	NT	NT	NT	NT	NT	NT	NT	NT	NT	NT	NT	NT	NT	NT	NT	NT	NT	NT
15	NT	NT	NT	NT	NT	NT	NT	NT	NT	NT	NT	NT	NT	NT	NT	NT	NT	NT	NT
16	NT	NT	NT	NT	NT	NT	NT	NT	NT	NT	NT	NT	NT	NT	NT	NT	NT	NT	NT
17	NT	NT	NT	NT	NT	NT	NT	NT	NT	NT	NT	NT	NT	NT	NT	NT	NT	NT	NT
18	NT	NT	NT	NT	NT	NT	NT	NT	NT	NT	NT	NT	NT	NT	NT	NT	NT	NT	NT
19	NT	NT	NT	NT	NT	NT	NT	NT	NT	NT	NT	NT	NT	NT	NT	NT	NT	NT	NT
20	NT	NT	NT	NT	NT	NT	NT	NT	NT	NT	NT	NT	NT	NT	NT	NT	NT	NT	NT
21	NT	NT	NT	NT	NT	NT	NT	NT	NT	NT	NT	NT	NT	NT	NT	NT	NT	NT	NT
22	NT	NT	NT	NT	NT	NT	NT	NT	NT	NT	NT	NT	NT	NT	NT	NT	NT	NT	NT
23	NT	NT	NT	NT	NT	NT	NT	NT	NT	NT	NT	NT	NT	NT	NT	NT	NT	NT	NT
24	NT	NT	NT	NT	NT	NT	NT	NT	NT	NT	NT	NT	NT	NT	NT	NT	NT	NT	NT
25	NT	NT	NT	NT	NT	NT	NT	NT	NT	NT	NT	NT	NT	NT	NT	NT	NT	NT	NT
26	NT	NT	NT	NT	NT	NT	NT	NT	NT	NT	NT	NT	NT	NT	NT	NT	NT	NT	NT
27	NT	NT	NT	NT	NT	NT	NT	NT	NT	NT	NT	NT	NT	NT	NT	NT	NT	NT	NT
28	NT	NT	NT	NT	NT	NT	NT	NT	NT	NT	NT	NT	NT	NT	NT	NT	NT	NT	NT
29	NT	NT	NT	NT	NT	NT	NT	NT	NT	NT	NT	NT	NT	NT	NT	NT	NT	NT	NT
30	NT	NT	NT	NT	NT	NT	NT	NT	NT	NT	NT	NT	NT	NT	NT	NT	NT	NT	NT
31	NT	NT	NT	NT	NT	NT	NT	NT	NT	NT	NT	NT	NT	NT	NT	NT	NT	NT	NT
32	NT	NT	NT	NT	NT	NT	NT	NT	NT	NT	NT	NT	NT	NT	NT	NT	NT	NT	NT
33	NT	NT	NT	NT	NT	NT	NT	NT	NT	NT	NT	NT	NT	NT	NT	NT	NT	NT	NT
34	NT	NT	NT	NT	NT	NT	NT	NT	NT	NT	NT	NT	NT	NT	NT	NT	NT	NT	NT
35	NT	NT	NT	NT	NT	NT	NT	NT	NT	NT	NT	NT	NT	NT	NT	NT	NT	NT	NT
36	NT	NT	NT	NT	NT	NT	NT	NT	NT	NT	NT	NT	NT	NT	NT	NT	NT	NT	NT
37	NT	NT	NT	NT	NT	NT	NT	NT	NT	NT	NT	NT	NT	NT	NT	NT	NT	NT	NT
38	NT	NT	NT	NT	NT	NT	NT	NT	NT	NT	NT	NT	NT	NT	NT	NT	NT	NT	NT
39	NT	NT	NT	NT	NT	NT	NT	NT	NT	NT	NT	NT	NT	NT	NT	NT	NT	NT	NT
40	NT	NT	NT	NT	NT	NT	NT	NT	NT	NT	NT	NT	NT	NT	NT	NT	NT	NT	NT
41	NT	NT	NT	NT	NT	NT	NT	NT	NT	NT	NT	NT	NT	NT	NT	NT	NT	NT	NT
42	NT	NT	NT	NT	NT	NT	NT	NT	NT	NT	NT	NT	NT	NT	NT	NT	NT	NT	NT
43	NT	NT	NT	NT	NT	NT	NT	NT	NT	NT	NT	NT	NT	NT	NT	NT	NT	NT	NT
44	NT	NT	NT	NT	NT	NT	NT	NT	NT	NT	NT	NT	NT	NT	NT	NT	NT	NT	NT
45	NT	NT	NT	NT	NT	NT	NT	NT	NT	NT	NT	NT	NT	NT	NT	NT	NT	NT	NT
46	NT	NT	NT	NT	NT	NT	NT	NT	NT	NT	NT	NT	NT	NT	NT	NT	NT	NT	NT
47	NT	NT	NT	NT	NT	NT	NT	NT	NT	NT	NT	NT	NT	NT	NT	NT	NT	NT	NT
48	NT	NT	NT	NT	NT	NT	NT	NT	NT	NT	NT	NT	NT	NT	NT	NT	NT	NT	NT
49	NT	NT	NT	NT	NT	NT	NT	NT	NT	NT	NT	NT	NT	NT	NT	NT	NT	NT	NT
50	NT	NT	NT	NT	NT	NT	NT	NT	NT	NT	NT	NT	NT	NT	NT	NT	NT	NT	NT
51	NT	NT	NT	NT	NT	NT	NT	NT	NT	NT	NT	NT	NT	NT	NT	NT	NT	NT	NT
52	NT	NT	NT	NT	NT	NT	NT	NT	NT	NT	NT	NT	NT	NT	NT	NT	NT	NT	NT
53	NT	NT	NT	NT	NT	NT	NT	NT	NT	NT	NT	NT	NT	NT	NT	NT	NT	NT	NT
54	NT	NT	NT	NT	NT	NT	NT	NT	NT	NT	NT	NT	NT	NT	NT	NT	NT	NT	NT
55	NT	NT	NT	NT	NT	NT	NT	NT	NT	NT	NT	NT	NT	NT	NT	NT	NT	NT	NT
56	NT	NT	NT	NT	NT	NT	NT	NT	NT	NT	NT	NT	NT	NT	NT	NT	NT	NT	NT
57	NT	NT	NT	NT	NT	NT	NT	NT	NT	NT	NT	NT	NT	NT	NT	NT	NT	NT	NT
58	NT	NT	NT	NT	NT	NT	NT	NT	NT	NT	NT	NT	NT	NT	NT	NT	NT	NT	NT
59	NT	NT	NT	NT	NT	NT	NT	NT	NT	NT	NT	NT	NT	NT	NT	NT	NT	NT	NT
60	NT	NT	NT	NT	NT	NT	NT	NT	NT	NT	NT	NT	NT	NT	NT	NT	NT	NT	NT
61	NT	NT	NT	NT	NT	NT	NT	NT	NT	NT	NT	NT	NT	NT	NT	NT	NT	NT	NT
62	NT	NT	NT	NT	NT	NT	NT	NT	NT	NT	NT	NT	NT	NT	NT	NT	NT	NT	NT
63	NT	NT	NT	NT	NT	NT	NT	NT	NT	NT	NT	NT	NT	NT	NT	NT	NT	NT	NT
64	NT	NT	NT	NT	NT	NT	NT	NT	NT	NT	NT	NT	NT	NT	NT	NT	NT	NT	NT
65	NT	NT	NT	NT	NT	NT	NT	NT	NT	NT	NT	NT	NT	NT	NT	NT	NT	NT	NT
66	NT	NT	NT	NT	NT	NT	NT	NT	NT	NT	NT	NT	NT	NT	NT	NT	NT	NT	NT
67	NT	NT	NT	NT	NT	NT	NT	NT	NT	NT	NT	NT	NT	NT	NT	NT	NT	NT	NT
68	NT	NT	NT	NT	NT	NT	NT	NT	NT	NT	NT	NT	NT	NT	NT	NT	NT	NT	NT
69	NT	NT	NT	NT	NT	NT	NT	NT	NT	NT	NT	NT	NT	NT	NT	NT	NT	NT	NT
70	NT	NT	NT	NT	NT	NT	NT	NT	NT	NT	NT	NT	NT	NT	NT	NT	NT	NT	NT
71	NT	NT	NT	NT	NT	NT	NT	NT	NT	NT	NT	NT	NT	NT	NT	NT	NT	NT	NT
72	NT	NT	NT	NT	NT	NT	NT	NT	NT	NT	NT	NT	NT	NT	NT	NT	NT	NT	NT
73	NT	NT	NT	NT	NT	NT	NT	NT	NT	NT	NT	NT	NT	NT	NT	NT	NT	NT	NT
74	NT	NT	NT	NT	NT	NT	NT	NT	NT	NT	NT	NT	NT	NT	NT	NT	NT	NT	NT
75	NT	NT	NT	NT	NT	NT	NT	NT	NT	NT	NT	NT	NT	NT	NT	NT	NT	NT	NT
76	NT	NT	NT	NT	NT	NT	NT	NT	NT	NT	NT	NT	NT	NT	NT	NT	NT	NT	NT
77	NT	NT	NT	NT	NT	NT	NT	NT	NT	NT	NT	NT	NT	NT	NT	NT	NT	NT	NT
78	NT	NT	NT	NT	NT	NT	NT	NT	NT	NT	NT	NT	NT	NT	NT	NT	NT	NT	NT
79	NT	NT	NT	NT	NT	NT	NT	NT	NT	NT	NT	NT	NT	NT	NT	NT	NT	NT	NT
80	NT	NT	NT	NT	NT	NT	NT	NT	NT	NT	NT	NT	NT	NT	NT	NT	NT	NT	NT
81	NT	NT	NT	NT	NT	NT	NT	NT	NT	NT	NT	NT	NT	NT	NT	NT	NT	NT	NT
82	NT	NT	NT	NT	NT	NT	NT	NT	NT	NT	NT	NT	NT	NT	NT	NT	NT	NT	NT
83	NT	NT	NT	NT	NT	NT	NT	NT	NT	NT	NT	NT	NT	NT	NT	NT	NT	NT	NT
84	NT	NT	NT	NT	NT	NT	NT	NT	NT	NT	NT	NT	NT	NT	NT	NT	NT	NT	NT
85	NT	NT	NT	NT	NT	NT	NT	NT	NT	NT	NT	NT	NT	NT	NT	NT	NT	NT	NT
86	NT	NT	NT	NT	NT	NT	NT	NT	NT	NT	NT	NT	NT	NT	NT	NT	NT	NT	NT
87	NT	NT	NT	NT	NT	NT	NT	NT	NT	NT	NT	NT	NT	NT	NT	NT	NT	NT	NT
88	NT	NT	NT	NT	NT	NT	NT	NT	NT	NT	NT	NT	NT	NT	NT	NT	NT	NT	NT
89	NT	NT	NT	NT	NT	NT	NT	NT	NT	NT	NT	NT	NT	NT	NT	NT	NT	NT	NT
90	NT	NT	NT	NT	NT	NT	NT	NT	NT	NT	NT	NT	NT	NT	NT	NT	NT	NT	NT
91	NT	NT	NT	NT	NT	NT	NT	NT	NT	NT	NT	NT	NT	NT	NT	NT	NT	NT	NT
92	NT	NT	NT	NT	NT	NT	NT	NT	NT	NT	NT	NT	NT	NT	NT	NT	NT	NT	NT
93	NT	NT	NT	NT	NT	NT	NT	NT	NT	NT	NT	NT	NT	NT	NT	NT	NT	NT	NT
94	NT	NT	NT	NT	NT	NT	NT	NT	NT	NT	NT	NT	NT	NT	NT	NT	NT	NT	NT
95	NT	NT	NT	NT	NT	NT	NT	NT	NT	NT	NT	NT	NT	NT	NT	NT	NT	NT	NT
96	NT	NT	NT	NT	NT	NT	NT	NT	NT	NT	NT	NT	NT	NT	NT	NT	NT	NT	NT
97	NT	NT	NT	NT	NT	NT	NT	NT	NT	NT	NT	NT	NT	NT	NT	NT	NT	NT	NT
98	NT	NT	NT	NT	NT	NT	NT	NT	NT	NT	NT	NT	NT	NT	NT	NT	NT	NT	NT
99	NT	NT	NT	NT	NT	NT	NT	NT	NT	NT	NT	NT	NT	NT	NT	NT	NT	NT	NT
100	NT	NT	NT	NT	NT	NT	NT	NT	NT	NT	NT	NT	NT	NT	NT	NT	NT	NT	NT

101	2β,15α,19-trihydroxy-ent-kaur-16-ene 2-O-β-d-gluco-pyranoside	NT	NT	NT	NT	NT	147.9	NT	NT	NT	NT	NT	NT	NT	NT	NT	NT	NT	NT	NT	NT	NT	NT	NT	NT	NT	NT	NT	NT	NT	NT	NT	NT	NT	NT	NT	NT	NT	NT	NT	NT	NT	NT	NT	NT
102	2β,6β,15α-trihydroxy-ent-kaur-16-ene	NT	NT	NT	NT	NT	137.1	NT	NT	NT	NT	NT	NT	NT	NT	NT	NT	NT	NT	NT	NT	NT	NT	NT	NT	NT	NT	NT	NT	NT	NT	NT	NT	NT	NT	NT	NT	NT	NT	NT	NT	NT	NT	NT	NT
103	pterokaurane P1	NT	NT	NT	NT	NT	10.8	NT	NT	NT	NT	NT	NT	NT	NT	NT	NT	NT	NT	NT	NT	NT	NT	NT	NT	NT	NT	NT	NT	NT	NT	NT	NT	NT	NT	NT	NT	NT	NT	NT	NT	NT	NT	NT	NT
104	pterokaurane P1 2-O-β-d-glucopyranoside	NT	NT	NT	NT	NT	101.4	NT	NT	NT	NT	NT	NT	NT	NT	NT	NT	NT	NT	NT	NT	NT	NT	NT	NT	NT	NT	NT	NT	NT	NT	NT	NT	NT	NT	NT	NT	NT	NT	NT	NT	NT	NT	NT	NT
105	2β,16α-dihydroxy-ent-kaurane	NT	NT	NT	NT	NT	121.7	NT	NT	NT	NT	NT	NT	NT	NT	NT	NT	NT	NT	NT	NT	NT	NT	NT	NT	NT	NT	NT	NT	NT	NT	NT	NT	NT	NT	NT	NT	NT	NT	NT	NT	NT	NT	NT	NT
106	2β,16α-dihydroxy-ent-kaurane 2,16-di-O-β-d-glucopyranoside	NT	NT	NT	NT	NT	182.2	NT	NT	NT	NT	NT	NT	NT	NT	NT	NT	NT	NT	NT	NT	NT	NT	NT	NT	NT	NT	NT	NT	NT	NT	NT	NT	NT	NT	NT	NT	NT	NT	NT	NT	NT	NT	NT	NT
107	Pterokaurane R	NT	NT	NT	NT	NT	171.3	NT	NT	NT	NT	NT	NT	NT	NT	NT	NT	NT	NT	NT	NT	NT	NT	NT	NT	NT	NT	NT	NT	NT	NT	NT	NT	NT	NT	NT	NT	NT	NT	NT	NT	NT	NT	NT	NT
108	2β,16α,17-trihydroxy-ent-kaurane 2-O-β-d-glucopyranoside	NT	NT	NT	NT	NT	113.7	NT	NT	NT	NT	NT	NT	NT	NT	NT	NT	NT	NT	NT	NT	NT	NT	NT	NT	NT	NT	NT	NT	NT	NT	NT	NT	NT	NT	NT	NT	NT	NT	NT	NT	NT	NT	NT	NT
109	Rabdonervosin G	NT	NT	NT	NT	NT	NT	NT	>20	NT	NT	NT	NT	NT	NT	NT	NT	NT	NT	NT	NT	NT	NT	NT	NT	NT	NT	>20	>20	>20	>20	NT	NT	NT	NT	NT	NT	NT	NT	NT	NT	NT	NT	NT	NT
110	Rabdonervosin H	NT	NT	NT	NT	NT	NT	NT	2.36	NT	NT	NT	NT	NT	NT	NT	NT	NT	NT	NT	NT	NT	NT	NT	NT	NT	NT	>20	6.07	>20	>20	NT	NT	NT	NT	NT	NT	NT	NT	NT	NT	NT	NT	NT	NT
111	Rabdonervosin I	NT	NT	NT	NT	NT	NT	NT	8.64	NT	NT	NT	NT	NT	NT	NT	NT	NT	NT	NT	NT	NT	NT	NT	NT	NT	NT	9.77	>20	>20	>20	NT	NT	NT	NT	NT	NT	NT	NT	NT	NT	NT	NT	NT	NT
112	Rabdonervosin J	NT	NT	NT	NT	NT	NT	NT	>20	NT	NT	NT	NT	NT	NT	NT	NT	NT	NT	NT	NT	NT	NT	NT	NT	NT	NT	>20	>20	>20	>20	NT	NT	NT	NT	NT	NT	NT	NT	NT	NT	NT	NT	NT	NT
113	Rabdonervosin D	NT	NT	NT	NT	NT	NT	NT	0.87	NT	NT	NT	NT	NT	NT	NT	NT	NT	NT	NT	NT	NT	NT	NT	NT	NT	NT	1.68	1.96	2.65	4.71	NT	NT	NT	NT	NT	NT	NT	NT	NT	NT	NT	NT	NT	NT
114	Sinuolatin E	NT	NT	NT	NT	NT	NT	NT	NT	NT	NT	NT	NT	NT	NT	NT	NT	NT	NT	NT	NT	NT	NT	NT	NT	NT	NT	NT	NT	NT	NT	NT	NT	NT	NT	NT	NT	NT	NT	NT	NT	NT	NT	NT	NT
115	Maoecrystal P	NT	NT	NT	NT	NT	NT	NT	NT	NT	NT	NT	NT	NT	NT	NT	NT	NT	NT	NT	NT	NT	NT	NT	NT	NT	NT	NT	NT	NT	NT	NT	NT	NT	NT	NT	NT	NT	NT	NT	NT	NT	NT	NT	NT
116	2,6-dihydroxy-*ent* -kaur-15-ene	NT	NT	NT	NT	NT	NT	NT	>10	>10	NT	NT	NT	NT	NT	NT	NT	NT	NT	NT	NT	NT	NT	NT	NT	NT	NT	NT	NT	NT	>10	>10	>10	NT	NT	NT	NT	NT	NT	NT	NT	NT	NT	NT	NT
117	2β ,6β -dihydroxy-*ent* -kaur-15-ene	NT	NT	NT	NT	NT	NT	NT	>10	>10	NT	NT	NT	NT	NT	NT	NT	NT	NT	NT	NT	NT	NT	NT	NT	NT	NT	NT	NT	NT	>10	>10	>10	NT	NT	NT	NT	NT	NT	NT	NT	NT	NT	NT	NT
118	*Ent* -3α,6β ,7α ,11α -tetraacetoxy-kaur-16-ene-15-one	NT	NT	NT	NT	NT	NT	NT	NT	NT	NT	NT	NT	NT	NT	NT	NT	NT	NT	NT	NT	NT	NT	NT	NT	NT	NT	NT	NT	NT	NT	NT	NT	NT	NT	NT	NT	NT	NT	NT	NT	NT	NT	NT	NT
119	*Ent-* 3α -hydroxy-7α,11α-diacetoxy-kaur-16-ene-6,15-dione	NT	NT	NT	NT	NT	NT	NT	NT	NT	NT	NT	NT	NT	NT	NT	NT	NT	NT	NT	NT	NT	NT	NT	NT	NT	NT	NT	NT	NT	NT	NT	NT	NT	NT	NT	NT	NT	NT	NT	NT	NT	NT	NT	NT
120	*Ent* -3α ,15α -dihydroxy-7a,11a-diacetoxy-kaur-16-ene-6-one	NT	NT	NT	NT	NT	NT	NT	NT	NT	NT	NT	NT	NT	NT	NT	NT	NT	NT	NT	NT	NT	NT	NT	NT	NT	NT	NT	NT	NT	NT	NT	NT	NT	NT	NT	NT	NT	NT	NT	NT	NT	NT	NT	NT
121	2β ,3β ,6β ,11β -tetraacetoxy-*ent* -kaur-16β -methy-15-one	NT	NT	NT	NT	NT	NT	NT	NT	NT	NT	NT	NT	NT	NT	NT	NT	NT	NT	NT	NT	NT	NT	NT	NT	NT	NT	NT	NT	NT	NT	NT	NT	NT	NT	NT	NT	NT	NT	NT	NT	NT	NT	NT	NT
122	7β -hydroxy-6β ,14β ,15β -triacetoxy-7α ,20-epoxy-*ent* -kaur-16-ene	NT	NT	NT	NT	NT	NT	NT	NT	NT	NT	NT	NT	NT	NT	NT	NT	NT	NT	NT	NT	NT	NT	NT	NT	NT	NT	NT	NT	NT	NT	NT	NT	NT	NT	NT	NT	NT	NT	NT	NT	NT	NT	NT	NT
123	Rabescensin C	NT	NT	NT	NT	NT	NT	NT	NT	NT	NT	NT	NT	NT	NT	NT	NT	NT	NT	NT	NT	NT	NT	NT	NT	NT	NT	NT	NT	NT	NT	NT	NT	NT	NT	NT	NT	NT	NT	NT	NT	NT	NT	NT	NT
124	Trichokaurin	NT	NT	NT	NT	NT	NT	NT	NT	NT	NT	NT	NT	NT	NT	NT	NT	NT	NT	NT	NT	NT	NT	NT	NT	NT	NT	NT	NT	NT	NT	NT	NT	NT	NT	NT	NT	NT	NT	NT	NT	NT	NT	NT	NT
125	wikstroemioidin E	>10	>10	>10	>10	6.9	NT	16.8	NT	NT	NT	NT	NT	NT	NT	NT	NT	NT	NT	NT	NT	NT	NT	NT	NT	NT	NT	NT	NT	NT	NT	NT	NT	NT	NT	NT	NT	NT	NT	NT	NT	NT	NT	NT	NT
126	wikstroemioidin F	>10	>10	>10	>10	8.6	NT	16.5	NT	NT	NT	NT	NT	NT	NT	NT	NT	NT	NT	NT	NT	NT	NT	NT	NT	NT	NT	NT	NT	NT	NT	NT	NT	NT	NT	NT	NT	NT	NT	NT	NT	NT	NT	NT	NT
127	wikstroemioidin G	2.9	3.5	2.7	1.6	0.9	NT	5	NT	NT	NT	NT	NT	NT	NT	NT	NT	NT	NT	NT	NT	NT	NT	NT	NT	NT	NT	NT	NT	NT	NT	NT	NT	NT	NT	NT	NT	NT	NT	NT	NT	NT	NT	NT	NT
128	wikstroemioidin H	3.6	4	4.4	4.9	1.4	NT	3.5	NT	NT	NT	NT	NT	NT	NT	NT	NT	NT	NT	NT	NT	NT	NT	NT	NT	NT	NT	NT	NT	NT	NT	NT	NT	NT	NT	NT	NT	NT	NT	NT	NT	NT	NT	NT	NT
129	wikstroemioidin I	>10	>10	>10	>10	>10	NT	NT	NT	NT	NT	NT	NT	NT	NT	NT	NT	NT	NT	NT	NT	NT	NT	NT	NT	NT	NT	NT	NT	NT	NT	NT	NT	NT	NT	NT	NT	NT	NT	NT	NT	NT	NT	NT	NT
130	wikstroemioidin J	>10	>10	>10	>10	>10	NT	NT	NT	NT	NT	NT	NT	NT	NT	NT	NT	NT	NT	NT	NT	NT	NT	NT	NT	NT	NT	NT	NT	NT	NT	NT	NT	NT	NT	NT	NT	NT	NT	NT	NT	NT	NT	NT	NT
131	wikstroemioidin K	>10	>10	>10	>10	>10	NT	NT	NT	NT	NT	NT	NT	NT	NT	NT	NT	NT	NT	NT	NT	NT	NT	NT	NT	NT	NT	NT	NT	NT	NT	NT	NT	NT	NT	NT	NT	NT	NT	NT	NT	NT	NT	NT	NT
132	wikstroemioidin L	>10	>10	>10	>10	>10	NT	NT	NT	NT	NT	NT	NT	NT	NT	NT	NT	NT	NT	NT	NT	NT	NT	NT	NT	NT	NT	NT	NT	NT	NT	NT	NT	NT	NT	NT	NT	NT	NT	NT	NT	NT	NT	NT	NT
133	wikstroemioidin M	4.1	4.1	3.7	4.8	2.7	NT	4.7	NT	NT	NT	NT	NT	NT	NT	NT	NT	NT	NT	NT	NT	NT	NT	NT	NT	NT	NT	NT	NT	NT	NT	NT	NT	NT	NT	NT	NT	NT	NT	NT	NT	NT	NT	NT	NT
134	wikstroemioidin N	NT	NT	NT	NT	NT	NT	NT	NT	NT	NT	NT	NT	NT	NT	NT	NT	NT	NT	NT	NT	NT	NT	NT	NT	NT	NT	NT	NT	NT	NT	NT	NT	NT	NT	NT	NT	NT	NT	NT	NT	NT	NT	NT	NT
135	wikstroemioidin O	4.8	5.1	5.1	3.7	2.3	NT	2.5	NT	NT	NT	NT	NT	NT	NT	NT	NT	NT	NT	NT	NT	NT	NT	NT	NT	NT	NT	NT	NT	NT	NT	NT	NT	NT	NT	NT	NT	NT	NT	NT	NT	NT	NT	NT	NT
136	wikstroemioidin P	3.1	0.8	1	1.6	1	NT	1.1	NT	NT	NT	NT	NT	NT	NT	NT	NT	NT	NT	NT	NT	NT	NT	NT	NT	NT	NT	NT	NT	NT	NT	NT	NT	NT	NT	NT	NT	NT	NT	NT	NT	NT	NT	NT	NT
137	wikstroemioidin Q	3.2	1.4	0.9	1.7	1	NT	1.9	NT	NT	NT	NT	NT	NT	NT	NT	NT	NT	NT	NT	NT	NT	NT	NT	NT	NT	NT	NT	NT	NT	NT	NT	NT	NT	NT	NT	NT	NT	NT	NT	NT	NT	NT	NT	NT
138	wikstroemioidin R	>10	>10	>10	>10	>10	NT	23.9	NT	NT	NT	NT	NT	NT	NT	NT	NT	NT	NT	NT	NT	NT	NT	NT	NT	NT	NT	NT	NT	NT	NT	NT	NT	NT	NT	NT	NT	NT	NT	NT	NT	NT	NT	NT	NT
139	wikstroemioidin S	NT	NT	NT	NT	NT	NT	NT	NT	NT	NT	NT	NT	NT	NT	NT	NT	NT	NT	NT	NT	NT	NT	NT	NT	NT	NT	NT	NT	NT	NT	NT	NT	NT	NT	NT	NT	NT	NT	NT	NT	NT	NT	NT	NT
140	wikstroemioidin T	>10	>10	>10	>10	>10	NT	NT	NT	NT	NT	NT	NT	NT	NT	NT	NT	NT	NT	NT	NT	NT	NT	NT	NT	NT	NT	NT	NT	NT	NT	NT	NT	NT	NT	NT	NT	NT	NT	NT	NT	NT	NT	NT	NT
141	wikstroemioidin U	>10	>10	>10	>10	>10	NT	9.9	NT	NT	NT	NT	NT	NT	NT	NT	NT	NT	NT	NT	NT	NT	NT	NT	NT	NT	NT	NT	NT	NT	NT	NT	NT	NT	NT	NT	NT	NT	NT	NT	NT	NT	NT	NT	NT
142	wikstroemioidin V	>10	7.1	>10	7.3	4	NT	2.8	NT	NT	NT	NT	NT	NT	NT	NT	NT	NT	NT	NT	NT	NT	NT	NT	NT	NT	NT	NT	NT	NT	NT	NT	NT	NT	NT	NT	NT	NT	NT	NT	NT	NT	NT	NT	NT
143	Nervonin G	>10	>10	>10	>10	>10	NT	NT	NT	NT	NT	NT	NT	NT	NT	NT	NT	NT	NT	NT	NT	NT	NT	NT	NT	NT	NT	NT	NT	NT	NT	NT	NT	NT	NT	NT	NT	NT	NT	NT	NT	NT	NT	NT	NT
144	Adenanthin J	>10	>10	>10	>10	>10	NT	NT	NT	NT	NT	NT	NT	NT	NT	NT	NT	NT	NT	NT	NT	NT	NT	NT	NT	NT	NT	NT	NT	NT	NT	NT	NT	NT	NT	NT	NT	NT	NT	NT	NT	NT	NT	NT	NT
145	Adenanthin K	>10	>10	>10	>10	>10	NT	NT	NT	NT	NT	NT	NT	NT	NT	NT	NT	NT	NT	NT	NT	NT	NT	NT	NT	NT	NT	NT	NT	NT	NT	NT	NT	NT	NT	NT	NT	NT	NT	NT	NT	NT	NT	NT	NT
146	Nervonin E	>10	>10	>10	>10	>10	NT	NT	NT	NT	NT	NT	NT	NT	NT	NT	NT	NT	NT	NT	NT	NT	NT	NT	NT	NT	NT	NT	NT	NT	NT	NT	NT	NT	NT	NT	NT	NT	NT	NT	NT	NT	NT	NT	NT
147	Adenanthin	3.6	4	3.3	2.8	2	NT	3.3	NT	NT	NT	NT	NT	NT	NT	NT	NT	NT	NT	NT	NT	NT	NT	NT	NT	NT	NT	NT	NT	NT	NT	NT	NT	NT	NT	NT	NT	NT	NT	NT	NT	NT	NT	NT	NT
148	Isoscoparins E	8.8	5.8	>10	>10	4.7	NT	7.2	NT	NT	NT	NT	NT	NT	NT	NT	NT	NT	NT	NT	NT	NT	NT	NT	NT	NT	NT	NT	NT	NT	NT	NT	NT	NT	NT	NT	NT	NT	NT	NT	NT	NT	NT	NT	NT
149	Isoscoparins L	0.5	1.2	0.8	0.4	0.6	NT	0.8	NT	NT	NT	NT	NT	NT	NT	NT	NT	NT	NT	NT	NT	NT	NT	NT	NT	NT	NT	NT	NT	NT	NT	NT	NT	NT	NT	NT	NT	NT	NT	NT	NT	NT	NT	NT	NT
150	Excisanin C	3	2.7	2.1	1.6	1.3	NT	1	NT	NT	NT	NT	NT	NT	NT	NT	NT	NT	NT	NT	NT	NT	NT	NT	NT	NT	NT	NT	NT	NT	NT	NT	NT	NT	NT	NT	NT	NT	NT	NT	NT	NT	NT	NT	NT
151	Albopilosin A	2	1.4	3.2	1.7	0.9	NT	1.9	NT	NT	NT	NT	NT	NT	NT	NT	NT	NT	NT	NT	NT	NT	NT	NT	NT	NT	NT	NT	NT	NT	NT	NT	NT	NT	NT	NT	NT	NT	NT	NT	NT	NT	NT	NT	NT
152	Phyllostachysin F	3.3	2.1	2.3	1.5	1.1	NT	1	NT	NT	NT	NT	NT	NT	NT	NT	NT	NT	NT	NT	NT	NT	NT	NT	NT	NT	NT	NT	NT	NT	NT	NT	NT	NT	NT	NT	NT	NT	NT	NT	NT	NT	NT	NT	NT
153	Rabdokunmins A	8.8	5.8	>10	>10	4.7	NT	7.2	NT	NT	NT	NT	NT	NT	NT	NT	NT	NT	NT	NT	NT	NT	NT	NT	NT	NT	NT	NT	NT	NT	NT	NT	NT	NT	NT	NT	NT	NT	NT	NT	NT	NT	NT	NT	NT
154	Rabdokunmins B	8.8	4	>10	>10	4	NT	4.8	NT	NT	NT	NT	NT	NT	NT	NT	NT	NT	NT	NT	NT	NT	NT	NT	NT	NT	NT	NT	NT	NT	NT	NT	NT	NT	NT	NT	NT	NT	NT	NT	NT	NT	NT	NT	NT
155	Rabdokunmins D	>10	>10	>10	>10	>10	NT	NT	NT	NT	NT	NT	NT	NT	NT	NT	NT	NT	NT	NT	NT	NT	NT	NT	NT	NT	NT	NT	NT	NT	NT	NT	NT	NT	NT	NT	NT	NT	NT	NT	NT	NT	NT	NT	NT
156	Macrocalyxin C	3.7	4	>10	3.7	2.1	NT	2.7	NT	NT	NT	NT	NT	NT	NT	NT	NT	NT	NT	NT	NT	NT	NT	NT	NT	NT	NT	NT	NT	NT	NT	NT	NT	NT	NT	NT	NT	NT	NT	NT	NT	NT	NT	NT	NT
157	Rbdoloxin B	2.9	3.4	4.4	3	2.4	NT	1.9	NT	NT	NT	NT	NT	NT	NT	NT	NT	NT	NT	NT	NT	NT	NT	NT	NT	NT	NT	NT	NT	NT	NT	NT	NT	NT	NT	NT	NT	NT	NT	NT	NT	NT	NT	NT	NT
158	Albopilosin B	>10	>10	>10	>10	>10	NT	NT	NT	NT	NT	NT	NT	NT	NT	NT	NT	NT	NT	NT	NT	NT	NT	NT	NT	NT	NT	NT	NT	NT	NT	NT	NT	NT	NT	NT	NT	NT	NT	NT	NT	NT	NT	NT	NT
159	3β ,7α ,14β ,18-tetrahydroxy-*ent* -kaur-16-ene12,15-dione	>10	>10	>10	>10	>10	NT	NT	NT	NT	NT	NT	NT	NT	NT	NT	NT	NT	NT	NT	NT	NT	NT	NT	NT	NT	NT	NT	NT	NT	NT	NT	NT	NT	NT	NT	NT	NT	NT	NT	NT	NT	NT	NT	NT
160	1α ,7α ,14β -trihydroxy-20-acetoxy-*ent* -kaur-15-one	NT	NT	NT	NT	NT	NT	NT	3.07 ± 0.0	5.59 ± 0.1	NT	NT	NT	NT	NT	NT	NT	NT	NT	NT	NT	NT	NT	NT	NT	NT	NT	NT	NT	NT	2.94 ± 0.0	>10	6.33 ± 0.3	NT	NT	NT	NT	NT	NT	NT	NT	NT	NT	NT	NT
161	1α ,7α ,14β ,18-tetrahydroxy-20-acetoxy-*ent* -kaur-15-one	NT	NT	NT	NT	NT	NT	NT	3.21 ± 0.0	4.17 ± 0.2	NT	NT	NT	NT	NT	NT	NT	NT	NT	NT	NT	NT	NT	NT	NT	NT	NT	NT	NT	NT	2.45 ± 0.1	>10	5.61 ± 0.1	NT	NT	NT	NT	NT	NT	NT	NT	NT	NT	NT	NT
162	1α -acetoxy-14β -hydroxy-7α ,20-epoxy-*ent* -kaur-16-en-15-one	NT	NT	NT	NT	NT	NT	NT	2.20 ± 1.1	>10	NT	NT	NT	NT	NT	NT	NT	NT	NT	NT	NT	NT	NT	NT	NT	NT	NT	NT	NT	NT	2.13 ± 0.8	5.68 ± 0.7	1.09 ± 0.1	NT	NT	NT	NT	NT	NT	NT	NT	NT	NT	NT	NT
163	Henryin	NT	NT	NT	NT	NT	NT	NT	1.54 ± 0.3	1.31 ± 0.7	NT	NT	NT	NT	NT	NT	NT	NT	NT	NT	NT	NT	NT	NT	NT	NT	NT	NT	NT	NT	1.77 ± 0.2	2.07 ± 0.3	1.42 ± 0.2	NT	NT	NT	NT	NT	NT	NT	NT	NT	NT	NT	NT
164	Kamebanin	NT	NT	NT	NT	NT	NT	NT	7.88 ± 1.0	2.99 ± 0.7	NT	NT	NT	NT	NT	NT	NT	NT	NT	NT	NT	NT	NT	NT	NT	NT	NT	NT	NT	NT	4.85 ± 0.3	>10	1.56 ± 0.3	NT	NT	NT	NT	NT	NT	NT	NT	NT	NT	NT	NT
165	Reniformin	NT	NT	NT	NT	NT	NT	NT	7.45 ± 0.2	5.17 ± 0.6	NT	NT	NT	NT	NT	NT	NT	NT	NT	NT	NT	NT	NT	NT	NT	NT	NT	NT	NT	NT	4.81 ± 0.0	>10	1.98 ± 0.1	NT	NT	NT	NT	NT	NT	NT	NT	NT	NT	NT	NT
166	Kamebacetal A	NT	NT	NT	NT	NT	NT	NT	1.09 ± 0.0	5.31 ± 0.1	NT	NT	NT	NT	NT	NT	NT	NT	NT	NT	NT	NT	NT	NT	NT	NT	NT	NT	NT	NT	4.87 ± 1.1	2.58 ± 0.2	1.44 ± 0.0	NT	NT	NT	NT	NT	NT	NT	NT	NT	NT	NT	NT
167	Kamebacetal B	NT	NT	NT	NT	NT	NT	NT	7.35 ± 0.4	4.97 ± 0.8	NT	NT	NT	NT	NT	NT	NT	NT	NT	NT	NT	NT	NT	NT	NT	NT	NT	NT	NT	NT	4.55 ± 0.0	2.38 ± 0.3	4.88 ± 0.2	NT	NT	NT	NT	NT	NT	NT	NT	NT	NT	NT	NT
168	Oridonin	NT	NT	NT	NT	NT	NT	NT	7.88 ± 1.0	2.99 ± 0.7	NT	NT	NT	NT	NT	NT	NT	NT	NT	NT	NT	NT	NT	NT	NT	NT	NT	NT	NT	NT	4.85 ± 0.3	>10	1.56 ± 0.3	NT	NT	NT	NT	NT	NT	NT	NT	NT	NT	NT	NT
169	*Ent* -7,20-epoxy- kaur-16-en-1α ,6β ,7β ,15β -tetrahydroxyl-11-O-β-d-glucopyranos	0.38 ± 0.0	0.96 ± 0.0	NT	NT	NT	NT	NT	NT	0.53 ± 0.0	NT	NT	NT	NT	NT	NT	NT	NT	NT	NT	NT	NT	NT	NT	NT	NT	NT	NT	NT	NT	NT	NT	1.15 ± 0.0	NT	NT	NT	NT	NT	NT	NT	NT	NT	NT	NT	NT
170	*Ent* -7,20-epoxy-kaur-16-en 6β ,7β ,14β ,15β -tetrahydroxyl-1-O-β-d-glucopyranos	0.35 ± 0.0	0.89 ± 0.0	NT	NT	NT	NT	NT	NT	0.53 ± 0.0	NT	NT	NT	NT	NT	NT	NT	NT	NT	NT	NT	NT	NT	NT	NT	NT	NT	NT	NT	NT	NT	NT	0.99 ± 0.0	NT	NT	NT	NT	NT	NT	NT	NT	NT	NT	NT	NT
171	*Ent* -7,20-epoxy-kaur-16-en-6β ,7β ,15β - trihydroxyl-1-O-β-d-glucopyranoside	0.26 ± 0.0	0.21 ± 0.0	NT	NT	NT	NT	NT	NT	0.27 ± 0.0	NT	NT	NT	NT	NT	NT	NT	NT	NT	NT	NT	NT	NT	NT	NT	NT	NT	NT	NT	NT	NT	NT	0.87 ± 0.0	NT	NT	NT	NT	NT	NT	NT	NT	NT	NT	NT	NT
172	*Ent* -7,20-epoxy-kaur-16-en-7β ,11β ,14α ,15β -tetrahydr- oxyl-6-O-β -d-glucopyrar	0.29 ± 0.0	0.61 ± 0.1	NT	NT	NT	NT	NT	NT	0.60 ± 0.0	NT	NT	NT	NT	NT	NT	NT	NT	NT	NT	NT	NT	NT	NT	NT	NT	NT	NT	NT	NT	NT	NT	2.44 ± 0.0	NT	NT	NT	NT	NT	NT	NT	NT	NT	NT	NT	NT
173	Enmenol-1-β -glucoside	0.28 ± 0.0	0.23 ± 0.0	NT	NT	NT	NT	NT	NT	0.24 ± 0.0	NT	NT	NT	NT	NT	NT	NT	NT	NT	NT	NT	NT	NT	NT	NT	NT	NT	NT	NT	NT	NT	NT	0.85 ± 0.0	NT	NT	NT	NT	NT	NT	NT	NT	NT	NT	NT	NT
174	Isodonterpene II	0.40 ± 0.0	0.65 ± 0.0	NT	NT	NT	NT	NT	NT	0.68 ± 0.0	NT	NT	NT	NT	NT	NT	NT	NT	NT	NT	NT	NT	NT	NT	NT	NT	NT	NT	NT	NT	NT	NT	0.86 ± 0.0	NT	NT	NT	NT	NT	NT	NT	NT	NT	NT	NT	NT
175	Enmenol	0.29 ± 0.4	0.18 ± 4.4	NT	NT	NT	NT	NT	NT	0.28 ± 0.0	NT	NT	NT	NT	NT	NT	NT	NT	NT	NT	NT	NT	NT	NT	NT	NT	NT	NT	NT	NT	NT	NT	0.38 ± 2.1	NT	NT	NT	NT	NT	NT	NT	NT	NT	NT	NT	NT
176	10α ,15α -dihydroxy-6,7-seco-1α ,7:6,11α - diolide-20-nor-*ent* -kaur-16-ene	NT	NT	NT	NT	NT	NT	NTb	NT	NT	NT	NT	NT	NT	NT	NT	NT	NT	NT	NT	NT	NT	NT	NT	NT	NT	NT	NT	NT	NT	NT	NT	NT	NT	NT	NT	NT	NT	NT	NT	NT	NT	NT	NT	NT
177	15α -acetoxy-6α - hydroxy-20-oxo-6,11-epoxy-6,7-seco-1α ,7-olide-*ent* -kaur-9- (11	>10	>10	>10	>10	>10	NT	>25b	NT	NT	NT	NT	NT	NT	NT	NT	NT	NT	NT	NT	NT	NT	NT	NT	NT	NT	NT	NT	NT	NT	NT	NT	NT	NT	NT	NT	NT	NT	NT	NT	NT	NT	NT	NT	NT
178	20(R)-15α -acetoxy-6β -hydroxy-20-methoxy- 6,20α -epoxy-6,7-seco-1α ,7-olide-*e*	>10	>10	>10	>10	>10	NT	>25b	NT	NT	NT	NT	NT	NT	NT	NT	NT	NT	NT	NT	NT	NT	NT	NT	NT	NT	NT	NT	NT	NT	NT	NT	NT	NT	NT	NT	NT	NT	NT	NT	NT	NT	NT	NT	NT
179	20(S*)-15α -acetoxy-6β ,11α -dihydroxy-20- methoxy-6,20α -epoxy-6,7-seco-1α ,7-	4.3	>10	>10	3.5	3.3	NT	1.5b	NT	NT	NT	NT	NT	NT	NT	NT	NT	NT	NT	NT	NT	NT	NT	NT	NT	NT	NT	NT	NT	NT	NT	NT	NT	NT	NT	NT	NT	NT	NT	NT	NT	NT	NT	NT	NT
180	20(R*)-6β ,11α ,15α -trihydroxy-20-methoxy-6,20α -epoxy-6,7- seco-1α ,7-olide-*en*	NT	NT	NT	NT	NT	NT	NTb	NT	NT	NT	NT	NT	NT	NT	NT	NT	NT	NT	NT	NT	NT	NT	NT	NT	NT	NT	NT	NT	NT	NT	NT	NT	NT	NT	NT	NT	NT	NT	NT	NT	NT	NT	NT	NT
181	11β ,14β - diacetoxy-6β ,7β -dihydroxy-7α ,20-epoxy-*ent* -kaur-16-en-15-one	4.6	>10	>10	>10	3.2	NT	6.3	NT	NT	NT	NT	NT	NT	NT	NT	NT	NT	NT	NT	NT	NT	NT	NT	NT	NT	NT	NT	NT	NT	NT	NT	NT	NT	NT	NT	NT	NT	NT	NT	NT	NT	NT	NT	NT
182	19-acetoxy-1β ,6β ,7β ,14β -tetrahydroxy-7α ,20- epoxy-*ent* -kaur-15-one	>10	>10	>10	>10	>10	NT	>25	NT	NT	NT	NT	NT	NT	NT	NT	NT	NT	NT	NT	NT	NT	NT	NT	NT	NT	NT	NT	NT	NT	NT	NT	NT	NT	NT	NT	NT	NT	NT	NT	NT	NT	NT	NT	NT
183	19-acetoxy-1β ,3β ,6β ,7β -tetrahy- droxy-7α ,20-epoxy-ent-kaur-15-one	>10	>10	>10	>10	>10	NT	>25	NT	NT	NT	NT	NT	NT	NT	NT	NT	NT	NT	NT	NT	NT	NT	NT	NT	NT	NT	NT	NT	NT	NT	NT	NT	NT	NT	NT	NT	NT	NT	NT	NT	NT	NT	NT	NT
184	6β ,7β ,11β ,14β ,15β - pentahydroxy-7α ,20-epoxy-*ent* -kaurane	>10	>10	>10	>10	>10	NT	>25	NT	NT	NT	NT	NT	NT	NT	NT	NT	NT	NT	NT	NT	NT	NT	NT	NT	NT	NT	NT	NT	NT	NT	NT	NT	NT	NT	NT	NT	NT	NT	NT	NT	NT	NT	NT	NT
185	6β ,7β ,14β ,15β - tetrahydroxy-7α ,20-olide-*ent* -kaurane	>10	>10	>10	>10	>10	NT	>25	NT	NT	NT	NT	NT	NT	NT	NT	NT	NT	NT	NT	NT	NT	NT	NT	NT	NT	NT	NT	NT	NT	NT	NT	NT	NT	NT	NT	NT	NT	NT	NT	NT	NT	NT	NT	NT
186	1α ,6β ,7β ,14β ,15β -pentahydroxy-7α ,20-epoxy-*ent* - kaur-9(11),16-diene	>10	>10	>10	>10	>10	NT	>25	NT	NT	NT	NT	NT	NT	NT	NT	NT	NT	NT	NT	NT	NT	NT	NT	NT	NT	NT	NT	NT	NT	NT	NT	NT	NT	NT	NT	NT	NT	NT	NT	NT	NT	NT	NT	NT
187	1α ,6β ,7β -trihydroxy-7α ,20-epoxy-*ent* -kaur-9(11),16-dien-15- one	8.8	3.5	4.6	3.3	3.4	NT	2	NT	NT	NT	NT	NT	NT	NT	NT	NT	NT	NT	NT	NT	NT	NT	NT	NT	NT	NT	NT	NT	NT	NT	NT	NT	NT	NT	NT	NT	NT	NT	NT	NT	NT	NT	NT	NT
188	20(R*)-20-ethoxy- 1α ,6β ,7β ,14β -tetrahydroxy-7α ,20-epoxy-*ent* -kaur-16-en-15-c	5.9b	>10b	>10	8.4	7.8	NT	3.9	NT	NT	NT	NT	NT	NT	NT	NT	NT	NT	NT	NT	NT	NT	NT	NT	NT	NT	NT	NT	NT	NT	NT	NT	NT	NT	NT	NT	NT	NT	NT	NT	NT	NT	NT	NT	NT
189	20(S*)-20-ethoxy-1α ,6β ,7β ,14β -tetrahydroxy-7α ,20- epoxy-*ent* -kaur-16-en-15-o	NT	NT	NT	NT	NT	NT	NT	NT	NT	NT	NT	NT	NT	NT	NT	NT	NT	NT	NT	NT	NT	NT	NT	NT	NT	NT	NT	NT	NT	NT	NT	NT	NT	NT	NT	NT	NT	NT	NT	NT	NT	NT	NT	NT
190	20(S*)-20-n-butoxy- 1α ,6β ,7β ,14β -tetrahydroxy-7α ,20-epoxy-*ent* -kaur-16-en-15	1.2	3.2	3.9	2.6	2.8	NT	1.5	NT	NT	NT	NT	NT	NT	NT	NT	NT	NT	NT	NT	NT	NT	NT	NT	NT	NT	NT	NT	NT	NT	NT	NT	NT	NT	NT	NT	NT	NT	NT	NT	NT	NT	NT	NT	NT
191	20(S*)- 1β ,7β ,14β ,20-tetrahydroxy-7,20-cyclo-kaur-16-ene-6,15-dione	NT	NT	NT	NT	NT	NT	NT	NT	NT	NT	NT	NT	NT	NT	NT	NT	NT	NT	NT	NT	NT	NT	NT	NT	NT	NT	NT	NT	NT	NT	NT	NT	NT	NT	NT	NT	NT	NT	NT	NT	NT	NT	NT	NT
192	15α -hydroxy-6,7-seco-1α ,7:11α,6-diolide-*ent* -kaur- 16-ene	6.1	>10	>10	>10	>10	NT	2	NT	NT	NT	NT	NT	NT	NT	NT	NT	NT	NT	NT	NT	NT	NT	NT	NT	NT	NT	NT	NT	NT	NT	NT	NT	NT	NT	NT	NT	NT	NT	NT	NT	NT	NT	NT	NT
193	Longikaurin G	3.5	4.5	8.6	5.1	2.5	NT	1.7	NT	NT	NT	NT	NT	NT	NT	NT	NT	NT	NT	NT	NT	NT	NT	NT	NT	NT	NT	NT	NT	NT	NT	NT	NT	NT	NT	NT	NT	NT	NT	NT	NT	NT	NT	NT	NT
194	Lasiokaurin	1.8	2.7	6.7	2.5	2.4	NT	1.9	NT	NT	NT	NT	NT	NT	NT	NT	NT	NT	NT	NT	NT	NT	NT	NT	NT	NT	NT	NT	NT	NT	NT	NT	NT	NT	NT	NT	NT	NT	NT	NT	NT	NT	NT	NT	NT
195	Isodonol	2.5	>10	>10	>10	>10	NT	5.2	NT	NT	NT	NT	NT	NT	NT	NT	NT	NT	NT	NT	NT	NT	NT	NT	NT	NT	NT	NT	NT	NT	NT	NT	NT	NT	NT	NT	NT	NT	NT	NT	NT	NT	NT	NT	NT
196	Effusanin A	7.9	5.9	5.6	4.2	3.2	NT	3.4	NT	NT	NT	NT	NT	NT	NT	NT	NT	NT	NT	NT	NT	NT	NT	NT	NT	NT	NT	NT	NT	NT	NT	NT	NT	NT	NT	NT	NT	NT	NT	NT	NT	NT	NT	NT	NT
197	Rabdoternin B	>10	>10	>10	>10	>10	NT	6.1	NT	NT	NT	NT	NT	NT	NT	NT	NT	NT	NT	NT	NT	NT	NT	NT	NT	NT	NT	NT	NT	NT	NT	NT	NT	NT	NT	NT	NT	NT	NT	NT	NT	NT	NT	NT	NT
198	Rabdoternin F	7	>10	>10	>10	9.4	NT	>25	NT	NT	NT	NT	NT	NT	NT	NT	NT	NT	NT	NT	NT	NT	NT	NT	NT	NT	NT	NT	NT	NT	NT	NT	NT	NT	NT	NT	NT	NT	NT	NT	NT	NT	NT	NT	NT
199	Rabdoternin E	2.6	>10	>10	>10	4.1	NT	2.3	NT	NT	NT	NT	NT	NT	NT	NT	NT	NT	NT	NT	NT	NT	NT	NT	NT	NT	NT	NT	NT	NT	NT	NT	NT	NT	NT	NT	NT	NT	NT	NT	NT	NT	NT	NT	NT
200	Rubescensin O	5.3	>10	>10	>10	>10	NT	>25	NT	NT	NT	NT	NT	NT	NT	NT	NT	NT	NT	NT	NT	NT	NT	NT	NT	NT	NT	NT	NT	NT	NT	NT	NT	NT	NT	NT	NT	NT	NT	NT	NT	NT	NT	NT	NT

201	*7α* ,20-epoxy-6*β* ,11*β* -dihydroxy-7*β* ,14*β* -[(1-methylethy-lidene)-*bis* -oxy]-*ent* -kau	1.5	2.6	3.4	1.8	2	NT	1.1	NT	NT	NT	NT	NT	NT	NT	NT	NT	NT	NT	NT	NT	NT	NT	NT	NT	NT	NT	NT	NT	NT	NT	NT	NT	NT	NT	NT	NT	NT	NT	NT	NT	NT	NT	NT	NT
202	Tenuifolin A	4.7	7.4	>10	>10	>10	NT	NT	NT	NT	NT	NT	NT	NT	NT	NT	NT	NT	NT	NT	NT	NT	NT	NT	NT	NT	NT	NT	NT	NT	NT	NT	NT	NT	NT	NT	NT	NT	NT	NT	NT	NT	NT	NT	NT
203	Tenuifolin B	>10	>10	>10	>10	>10	NT	6.9	NT	NT	NT	NT	NT	NT	NT	NT	NT	NT	NT	NT	NT	NT	NT	NT	NT	NT	NT	NT	NT	NT	NT	NT	NT	NT	NT	NT	NT	NT	NT	NT	NT	NT	NT	NT	NT
204	Tenuifolin C	>10	>10	>10	>10	>10	NT	>25	NT	NT	NT	NT	NT	NT	NT	NT	NT	NT	NT	NT	NT	NT	NT	NT	NT	NT	NT	NT	NT	NT	NT	NT	NT	NT	NT	NT	NT	NT	NT	NT	NT	NT	NT	NT	NT
205	Tenuifolin D	>10	>10	>10	>10	>10	NT	>25	NT	NT	NT	NT	NT	NT	NT	NT	NT	NT	NT	NT	NT	NT	NT	NT	NT	NT	NT	NT	NT	NT	NT	NT	NT	NT	NT	NT	NT	NT	NT	NT	NT	NT	NT	NT	NT
206	Tenuifolin E	>10	>10	>10	>10	>10	NT	>25	NT	NT	NT	NT	NT	NT	NT	NT	NT	NT	NT	NT	NT	NT	NT	NT	NT	NT	NT	NT	NT	NT	NT	NT	NT	NT	NT	NT	NT	NT	NT	NT	NT	NT	NT	NT	NT
207	Tenuifolin F	>10	>10	>10	>10	>10	NT	>25	NT	NT	NT	NT	NT	NT	NT	NT	NT	NT	NT	NT	NT	NT	NT	NT	NT	NT	NT	NT	NT	NT	NT	NT	NT	NT	NT	NT	NT	NT	NT	NT	NT	NT	NT	NT	NT
208	Tenuifolin G	>10	>10	>10	>10	>10	NT	>25	NT	NT	NT	NT	NT	NT	NT	NT	NT	NT	NT	NT	NT	NT	NT	NT	NT	NT	NT	NT	NT	NT	NT	NT	NT	NT	NT	NT	NT	NT	NT	NT	NT	NT	NT	NT	NT
209	Tenuifolin H	>10	>10	>10	>10	>10	NT	>25	NT	NT	NT	NT	NT	NT	NT	NT	NT	NT	NT	NT	NT	NT	NT	NT	NT	NT	NT	NT	NT	NT	NT	NT	NT	NT	NT	NT	NT	NT	NT	NT	NT	NT	NT	NT	NT
210	Tenuifolin I	0.8	1.9	4.5	3	4.3	NT	>25	NT	NT	NT	NT	NT	NT	NT	NT	NT	NT	NT	NT	NT	NT	NT	NT	NT	NT	NT	NT	NT	NT	NT	NT	NT	NT	NT	NT	NT	NT	NT	NT	NT	NT	NT	NT	NT
211	Tenuifolin J	3.8	6.3	>10	>10	>10	NT	5.8	NT	NT	NT	NT	NT	NT	NT	NT	NT	NT	NT	NT	NT	NT	NT	NT	NT	NT	NT	NT	NT	NT	NT	NT	NT	NT	NT	NT	NT	NT	NT	NT	NT	NT	NT	NT	NT
212	Tenuifolin K	>10	>10	>10	>10	>10	NT	8.8	NT	NT	NT	NT	NT	NT	NT	NT	NT	NT	NT	NT	NT	NT	NT	NT	NT	NT	NT	NT	NT	NT	NT	NT	NT	NT	NT	NT	NT	NT	NT	NT	NT	NT	NT	NT	NT
213	Tenuifolin L	>10	>10	>10	>10	>10	NT	>25	NT	NT	NT	NT	NT	NT	NT	NT	NT	NT	NT	NT	NT	NT	NT	NT	NT	NT	NT	NT	NT	NT	NT	NT	NT	NT	NT	NT	NT	NT	NT	NT	NT	NT	NT	NT	NT
214	Tenuifolin M	4.4	>10	>10	>10	>10	NT	>25	NT	NT	NT	NT	NT	NT	NT	NT	NT	NT	NT	NT	NT	NT	NT	NT	NT	NT	NT	NT	NT	NT	NT	NT	NT	NT	NT	NT	NT	NT	NT	NT	NT	NT	NT	NT	NT
215	Adenanthin C	1.8	3.2	>10	4.8	5.1	NT	3.8	NT	NT	NT	NT	NT	NT	NT	NT	NT	NT	NT	NT	NT	NT	NT	NT	NT	NT	NT	NT	NT	NT	NT	NT	NT	NT	NT	NT	NT	NT	NT	NT	NT	NT	NT	NT	NT
216	Adenanthin B	>10	>10	>10	>10	>10	NT	>25	NT	NT	NT	NT	NT	NT	NT	NT	NT	NT	NT	NT	NT	NT	NT	NT	NT	NT	NT	NT	NT	NT	NT	NT	NT	NT	NT	NT	NT	NT	NT	NT	NT	NT	NT	NT	NT
217	Adenanthin E	>10	>10	>10	>10	>10	NT	>25	NT	NT	NT	NT	NT	NT	NT	NT	NT	NT	NT	NT	NT	NT	NT	NT	NT	NT	NT	NT	NT	NT	NT	NT	NT	NT	NT	NT	NT	NT	NT	NT	NT	NT	NT	NT	NT
218	Inflexarabdonin E	1.7	2.2	6.8	4.8	4.6	NT	5.7	NT	NT	NT	NT	NT	NT	NT	NT	NT	NT	NT	NT	NT	NT	NT	NT	NT	NT	NT	NT	NT	NT	NT	NT	NT	NT	NT	NT	NT	NT	NT	NT	NT	NT	NT	NT	NT
219	*Ent* -7*α* -acetoxy-16*β* ,18-dihydroxy-kaurane	NT	NT	NT	NT	NT	NT	NT	NT	NT	NT	NT	NT	NT	NT	NT	NT	NT	NT	NT	NT	NT	NT	NT	NT	NT	NT	NT	NT	NT	NT	NT	NT	NT	NT	NT	NT	NT	NT	NT	94.52 ± 0.07	303.53 ± 1.22	NA	1.89 ± 0.08	1.19 ± 1.67
220	Epoxysolinearol	NT	NT	NT	NT	NT	NT	NT	NT	NT	NT	NT	NT	NT	NT	NT	NT	NT	NT	NT	NT	NT	NT	NT	NT	NT	NT	NT	NT	NT	NT	NT	NT	NT	NT	NT	NT	NT	NT	NT	195.17 ± 1.11	337.52 ± 2.09	NA	0.87 ± 0.01	0.43 ± 0.02
221	Sideroxol	NT	NT	NT	NT	NT	NT	NT	NT	NT	NT	NT	NT	NT	NT	NT	NT	NT	NT	NT	NT	NT	NT	NT	NT	NT	NT	NT	NT	NT	NT	NT	NT	NT	NT	NT	NT	NT	NT	NT	NA	388.3 ± 3.26	NA	1.27 ± 0.80	0.024 ± 0.00
222	Sideridiol	NT	NT	NT	NT	NT	NT	NT	NT	NT	NT	NT	NT	NT	NT	NT	NT	NT	NT	NT	NT	NT	NT	NT	NT	NT	NT	NT	NT	NT	NT	NT	NT	NT	NT	NT	NT	NT	NT	NT	NA	273.85 ± 1.53	NA	8.04 ± 0.87	3.67 ± 0.99
223	Siderol	NT	NT	NT	NT	NT	NT	NT	NT	NT	NT	NT	NT	NT	NT	NT	NT	NT	NT	NT	NT	NT	NT	NT	NT	NT	NT	NT	NT	NT	NT	NT	NT	NT	NT	NT	NT	NT	NT	NT	NA	298.95 ± 2.55	NA	0.69 ± 0.81	0.65 ± 0.09
224	7-epicandicandiol	NT	NT	NT	NT	NT	NT	NT	NT	NT	NT	NT	NT	NT	NT	NT	NT	NT	NT	NT	NT	NT	NT	NT	NT	NT	NT	NT	NT	NT	NT	NT	NT	NT	NT	NT	NT	NT	NT	NT	53.82 ± 0.59	514.38 ± 4.65	NA	0.23 ± 0.09	0.022 ± 0.01
225	Foliol	NT	NT	NT	NT	NT	NT	NT	NT	NT	NT	NT	NT	NT	NT	NT	NT	NT	NT	NT	NT	NT	NT	NT	NT	NT	NT	NT	NT	NT	NT	NT	NT	NT	NT	NT	NT	NT	NT	NT	NT	NT	NT	NT	NT
226	Linearol	NT	NT	NT	NT	NT	NT	NT	NT	NT	NT	NT	NT	NT	NT	NT	NT	NT	NT	NT	NT	NT	NT	NT	NT	NT	NT	NT	NT	NT	NT	NT	NT	NT	NT	NT	NT	NT	NT	NT	271.70 ± 2.05	571.05 ± 2.19	NA	2.66 ± 1.27	0.15 ± 0.00
227	Sidol	NT	NT	NT	NT	NT	NT	NT	NT	NT	NT	NT	NT	NT	NT	NT	NT	NT	NT	NT	NT	NT	NT	NT	NT	NT	NT	NT	NT	NT	NT	NT	NT	NT	NT	NT	NT	NT	NT	NT	355.82 ± 0.38	548.14 ± 3.29	NA	0.92 ± 0.01	0.05 ± 0.00
228	Sinuolatins A	NT	NT	>10	4.3	NT	NT	NT	NT	NT	NT	NT	NT	>10	NT	NT	NT	NT	NT	NT	NT	NT	NT	NT	NT	NT	NT	NT	NT	NT	NT	NT	NT	NT	NT	NT	1.7			2.7	NT	NT	NT	NT	NT
229	Sinuolatins B	NT	NT	>10	>10	NT	NT	NT	NT	NT	NT	NT	NT	>10	NT	NT	NT	NT	NT	NT	NT	NT	NT	NT	NT	NT	NT	NT	NT	NT	NT	NT	NT	NT	NT	NT	3.6			8.1	NT	NT	NT	NT	NT
230	Sinuolatins C	NT	NT	NA	>10	NT	NT	NT	NT	NT	NT	NT	NT	>10	NT	NT	NT	NT	NT	NT	NT	NT	NT	NT	NT	NT	NT	NT	NT	NT	NT	NT	NT	NT	NT	NT	NA			NA	NT	NT	NT	NT	NT
231	Sinuolatins D	NT	NT	NA	>10	NT	NT	NT	NT	NT	NT	NT	NT	>10	NT	NT	NT	NT	NT	NT	NT	NT	NT	NT	NT	NT	NT	NT	NT	NT	NT	NT	NT	NT	NT	NT	NA			NA	NT	NT	NT	NT	NT
232	Sinuolatins E	NT	NT	>10	>10	NT	NT	NT	NT	NT	NT	NT	NT	>10	NT	NT	NT	NT	NT	NT	NT	NT	NT	NT	NT	NT	NT	NT	NT	NT	NT	NT	NT	NT	NT	NT	>10			6.5	NT	NT	NT	NT	NT
233	Maoecrystal A	NT	NT	>10	>10	NT	NT	NT	NT	NT	NT	NT	NT	>10	NT	NT	NT	NT	NT	NT	NT	NT	NT	NT	NT	NT	NT	NT	NT	NT	NT	NT	NT	NT	NT	NT	6.5			>10	NT	NT	NT	NT	NT
234	Maoecrystal G	NT	NT	NA	>10	NT	NT	NT	NT	NT	NT	NT	NT	>10	NT	NT	NT	NT	NT	NT	NT	NT	NT	NT	NT	NT	NT	NT	NT	NT	NT	NT	NT	NT	NT	NT	NA			>10	NT	NT	NT	NT	NT
235	Longikaurin E	NT	NT	NA	>10	NT	NT	NT	NT	NT	NT	NT	NT	>10	NT	NT	NT	NT	NT	NT	NT	NT	NT	NT	NT	NT	NT	NT	NT	NT	NT	NT	NT	NT	NT	NT	NA			>10	NT	NT	NT	NT	NT
236	Lasiodonin	NT	NT	NA	>10	NT	NT	NT	NT	NT	NT	NT	NT	>10	NT	NT	NT	NT	NT	NT	NT	NT	NT	NT	NT	NT	NT	NT	NT	NT	NT	NT	NT	NT	NT	NT	NA			>10	NT	NT	NT	NT	NT
237	Nodosin	NT	NT	>10	>10	NT	NT	NT	NT	NT	NT	NT	NT	>10	NT	NT	NT	NT	NT	NT	NT	NT	NT	NT	NT	NT	NT	NT	NT	NT	NT	NT	NT	NT	NT	NT	3.5			>10	NT	NT	NT	NT	NT
238	Oreskaurin C	NT	NT	NA	>10	NT	NT	NT	NT	NT	NT	NT	NT	>10	NT	NT	NT	NT	NT	NT	NT	NT	NT	NT	NT	NT	NT	NT	NT	NT	NT	NT	NT	NT	NT	NT	NA			NA	NT	NT	NT	NT	NT
239	Rubescensin Q	NT	NT	>10	>10	NT	NT	NT	NT	NT	NT	NT	NT	>10	NT	NT	NT	NT	NT	NT	NT	NT	NT	NT	NT	NT	NT	NT	NT	NT	NT	NT	NT	NT	NT	NT	7.7			>10	NT	NT	NT	NT	NT
240	Enmenin	NT	NT	>10	>10	NT	NT	NT	NT	NT	NT	NT	NT	>10	NT	NT	NT	NT	NT	NT	NT	NT	NT	NT	NT	NT	NT	NT	NT	NT	NT	NT	NT	NT	NT	NT	9.1			>10	NT	NT	NT	NT	NT
241	Trichorabdal A	NT	NT	7.7	6.3	NT	NT	NT	NT	NT	NT	NT	NT	2.9	NT	NT	NT	NT	NT	NT	NT	NT	NT	NT	NT	NT	NT	NT	NT	NT	NT	NT	NT	NT	NT	NT	2.7			8.8	NT	NT	NT	NT	NT
242	Adenolin A	NT	NT	NA	>10	NT	NT	NT	NT	NT	NT	NT	NT	>10	NT	NT	NT	NT	NT	NT	NT	NT	NT	NT	NT	NT	NT	NT	NT	NT	NT	NT	NT	NT	NT	NT	NA			NA	NT	NT	NT	NT	NT
243	Oreskaurin B	NT	NT	>10	>10	NT	NT	NT	NT	NT	NT	NT	NT	8.2	NT	NT	NT	NT	NT	NT	NT	NT	NT	NT	NT	NT	NT	NT	NT	NT	NT	NT	NT	NT	NT	NT	8.8			>10	NT	NT	NT	NT	NT
244	Isodonal	NT	NT	>10	>10	NT	NT	NT	NT	NT	NT	NT	NT	>10	NT	NT	NT	NT	NT	NT	NT	NT	NT	NT	NT	NT	NT	NT	NT	NT	NT	NT	NT	NT	NT	NT	1.1			>10	NT	NT	NT	NT	NT
245	Adenolin B	NT	NT	>10	>10	NT	NT	NT	NT	NT	NT	NT	NT	>10	NT	NT	NT	NT	NT	NT	NT	NT	NT	NT	NT	NT	NT	NT	NT	NT	NT	NT	NT	NT	NT	NT	>10			>10	NT	NT	NT	NT	NT
246	Wikstroemioidin B	NT	NT	NA	>10	NT	NT	NT	NT	NT	NT	NT	NT	>10	NT	NT	NT	NT	NT	NT	NT	NT	NT	NT	NT	NT	NT	NT	NT	NT	NT	NT	NT	NT	NT	NT	NA			NA	NT	NT	NT	NT	NT
247	Wikstroemioidin C	NT	NT	>10	>10	NT	NT	NT	NT	NT	NT	NT	NT	>10	NT	NT	NT	NT	NT	NT	NT	NT	NT	NT	NT	NT	NT	NT	NT	NT	NT	NT	NT	NT	NT	NT	7.4			>10	NT	NT	NT	NT	NT
248	Macrocalyxin J	NT	NT	5.3	>10	NT	NT	NT	NT	NT	NT	NT	NT	>10	NT	NT	NT	NT	NT	NT	NT	NT	NT	NT	NT	NT	NT	NT	NT	NT	NT	NT	NT	NT	NT	NT	5.4			6.6	NT	NT	NT	NT	NT
249	Effusanin E	NT	NT	>10	>10	NT	NT	NT	NT	NT	NT	NT	NT	>10	NT	NT	NT	NT	NT	NT	NT	NT	NT	NT	NT	NT	NT	NT	NT	NT	NT	NT	NT	NT	NT	NT	4			7.7	NT	NT	NT	NT	NT
250	Lushanrubescensin F	NT	NT	NT	NT	NT	NT	NT	NT	NT	NT	NT	NT	NT	NT	NT	NT	NT	NT	NT	NT	NT	NT	NT	NT	NT	NT	NT	NT	NT	NT	NT	NT	NT	NT	NT	NT	NT	NT	NT	NT	NT	NT	NT	NT
251	Longikaurin B	NT	NT	NT	NT	NT	NT	NT	NT	NT	NT	NT	NT	NT	NT	NT	NT	NT	NT	NT	NT	NT	NT	NT	NT	NT	NT	NT	NT	NT	NT	NT	NT	NT	NT	NT	NT	NT	NT	NT	NT	NT	NT	NT	NT
252	Geopyxin A	NT	NT	NT	NA	NT	NT	NT	NT	NT	NT	NT	NT	NA	NT	NT	NT	NT	NT	NT	NT	NT	NT	NT	NT	NT	NT	NT	NT	NT	NT	NT	NT	NA	NA	NA	NT	NT	NT	NT	NT	NT	NT	NT	NT
253	Geopyxin B	NT	NT	NT	4.32 ± 1.5	NT	NT	NT	NT	NT	NT	NT	NT	5.41 ± 0.9	NT	NT	NT	NT	NT	NT	NT	NT	NT	NT	NT	NT	NT	NT	NT	NT	NT	NT	NT	2.25 ± 0.1	2.35 ± 0.3	3.31 ± 0.4	NT	NT	NT	NT	NT	NT	NT	NT	NT
254	Geopyxin C	NT	NT	NT	NA	NT	NT	NT	NT	NT	NT	NT	NT	NA	NT	NT	NT	NT	NT	NT	NT	NT	NT	NT	NT	NT	NT	NT	NT	NT	NT	NT	NT	NA	NA	NA	NT	NT	NT	NT	NT	NT	NT	NT	NT
255	Geopyxin D	NT	NT	NT	NA	NT	NT	NT	NT	NT	NT	NT	NT	NA	NT	NT	NT	NT	NT	NT	NT	NT	NT	NT	NT	NT	NT	NT	NT	NT	NT	NT	NT	NA	NA	NA	NT	NT	NT	NT	NT	NT	NT	NT	NT
256	Geopyxin E	NT	NT	NT	NA	NT	NT	NT	NT	NT	NT	NT	NT	NA	NT	NT	NT	NT	NT	NT	NT	NT	NT	NT	NT	NT	NT	NT	NT	NT	NT	NT	NT	NA	NA	NA	NT	NT	NT	NT	NT	NT	NT	NT	NT
257	Geopyxin F	NT	NT	NT	NA	NT	NT	NT	NT	NT	NT	NT	NT	NA	NT	NT	NT	NT	NT	NT	NT	NT	NT	NT	NT	NT	NT	NT	NT	NT	NT	NT	NT	NA	NA	NA	NT	NT	NT	NT	NT	NT	NT	NT	NT
258	Methylgeopyxin A	NT	NT	NT	0.79 ± 0.0	NT	NT	NT	NT	NT	NT	NT	NT	2.27 ± 0.0	NT	NT	NT	NT	NT	NT	NT	NT	NT	NT	NT	NT	NT	NT	NT	NT	NT	NT	NT	1.08 ± 0.1	0.72 ± 0.0	0.73 ± 0.1	NT	NT	NT	NT	NT	NT	NT	NT	NT
259	Methylgeopyxin B	NT	NT	NT	0.63 ± 0.1	NT	NT	NT	NT	NT	NT	NT	NT	1.48 ± 0.1	NT	NT	NT	NT	NT	NT	NT	NT	NT	NT	NT	NT	NT	NT	NT	NT	NT	NT	NT	0.92 ± 0.0	0.69 ± 0.0	0.91 ± 0.1	NT	NT	NT	NT	NT	NT	NT	NT	NT
260	Methylgeopyxin C	NT	NT	NT	1.67 ± 0.4	NT	NT	NT	NT	NT	NT	NT	NT	3.63 ± 0.0	NT	NT	NT	NT	NT	NT	NT	NT	NT	NT	NT	NT	NT	NT	NT	NT	NT	NT	NT	2.28 ± 0.1	1.56 ± 0.1	3.38 ± 0.3	NT	NT	NT	NT	NT	NT	NT	NT	NT
261	Methylgeopyxin E	NT	NT	NT	2.08 ± 0.2	NT	NT	NT	NT	NT	NT	NT	NT	3.99 ± 0.3	NT	NT	NT	NT	NT	NT	NT	NT	NT	NT	NT	NT	NT	NT	NT	NT	NT	NT	NT	2.27 ± 0.1	1.02 ± 0.0	3.81 ± 0.4	NT	NT	NT	NT	NT	NT	NT	NT	NT
262	Methylgeopyxin F	NT	NT	NT	NA	NT	NT	NT	NT	NT	NT	NT	NT	NA	NT	NT	NT	NT	NT	NT	NT	NT	NT	NT	NT	NT	NT	NT	NT	NT	NT	NT	NT	NA	NA	NA	NT	NT	NT	NT	NT	NT	NT	NT	NT
263	1-*O* -Acetylgeopyxin A	NT	NT	NT	3.48 ± 0.2	NT	NT	NT	NT	NT	NT	NT	NT	3.88 ± 0.2	NT	NT	NT	NT	NT	NT	NT	NT	NT	NT	NT	NT	NT	NT	NT	NT	NT	NT	NT	3.52 ± 0.2	2.37 ± 0.1	4.11 ± 0.1	NT	NT	NT	NT	NT	NT	NT	NT	NT
264	1,7-*O* -Diacetylgeopyxin A	NT	NT	NT	NA	NT	NT	NT	NT	NT	NT	NT	NT	NA	NT	NT	NT	NT	NT	NT	NT	NT	NT	NT	NT	NT	NT	NT	NT	NT	NT	NT	NT	NA	NA	NA	NT	NT	NT	NT	NT	NT	NT	NT	NT
265	1-*O* -Acetylmethylgeopyxin A	NT	NT	NT	0.53 ± 0.1	NT	NT	NT	NT	NT	NT	NT	NT	1.54 ± 0.0	NT	NT	NT	NT	NT	NT	NT	NT	NT	NT	NT	NT	NT	NT	NT	NT	NT	NT	NT	0.82 ± 0.0	0.66 ± 0.0	1.15 ± 0.0	NT	NT	NT	NT	NT	NT	NT	NT	NT
266	1,7-*O* -Diacetylmethylgeopyxin A	NT	NT	NT	1.18 ± 0.0	NT	NT	NT	NT	NT	NT	NT	NT	0.24 ± 0.0	NT	NT	NT	NT	NT	NT	NT	NT	NT	NT	NT	NT	NT	NT	NT	NT	NT	NT	NT	1.26 ± 0.0	1.18 ± 0.0	1.93 ± 0.1	NT	NT	NT	NT	NT	NT	NT	NT	NT
267	Isodojaponin A	NT	NT	NT	NT	NT	NT	6.3 ± 0.22	NT	NT	NT	NT	NT	NT	NT	NT	NT	NT	NT	NT	NT	NT	NT	NT	NT	NT	NT	NT	NT	NT	NT	NT	NT	NT	NT	NT	NT	NT	NT	NT	NT	NT	NT	NT	NT
268	Isodojaponin B	NT	NT	NT	NT	NT	NT	10.2 ± 0.25	NT	NT	NT	NT	NT	NT	NT	NT	NT	NT	NT	NT	NT	NT	NT	NT	NT	NT	NT	NT	NT	NT	NT	NT	NT	NT	NT	NT	NT	NT	NT	NT	NT	NT	NT	NT	NT
269	Isodojaponin C	NT	NT	NT	NT	NT	NT	8.2 ± 0.12	NT	NT	NT	NT	NT	NT	NT	NT	NT	NT	NT	NT	NT	NT	NT	NT	NT	NT	NT	NT	NT	NT	NT	NT	NT	NT	NT	NT	NT	NT	NT	NT	NT	NT	NT	NT	NT
270	Isodojaponin D	NT	NT	NT	NT	NT	NT	8.7 ± 0.27	NT	NT	NT	NT	NT	NT	NT	NT	NT	NT	NT	NT	NT	NT	NT	NT	NT	NT	NT	NT	NT	NT	NT	NT	NT	NT	NT	NT	NT	NT	NT	NT	NT	NT	NT	NT	NT
271	Isodojaponin E	NT	NT	NT	NT	NT	NT	20.3 ± 0.33	NT	NT	NT	NT	NT	NT	NT	NT	NT	NT	NT	NT	NT	NT	NT	NT	NT	NT	NT	NT	NT	NT	NT	NT	NT	NT	NT	NT	NT	NT	NT	NT	NT	NT	NT	NT	NT
272	Pseurata C	NT	NT	NT	NT	NT	NT	5.4 ± 0.09	NT	NT	NT	NT	NT	NT	NT	NT	NT	NT	NT	NT	NT	NT	NT	NT	NT	NT	NT	NT	NT	NT	NT	NT	NT	NT	NT	NT	NT	NT	NT	NT	NT	NT	NT	NT	NT
273	Longikaurin C	NT	NT	NT	NT	NT	NT	1.0 ± 0.15	NT	NT	NT	NT	NT	NT	NT	NT	NT	NT	NT	NT	NT	NT	NT	NT	NT	NT	NT	NT	NT	NT	NT	NT	NT	NT	NT	NT	NT	NT	NT	NT	NT	NT	NT	NT	NT
274	Effusanin C	NT	NT	NT	NT	NT	NT	5.9 ± 0.06	NT	NT	NT	NT	NT	NT	NT	NT	NT	NT	NT	NT	NT	NT	NT	NT	NT	NT	NT	NT	NT	NT	NT	NT	NT	NT	NT	NT	NT	NT	NT	NT	NT	NT	NT	NT	NT
275	Longikaurin B	NT	NT	NT	NT	NT	NT	0.9 ± 0.08	NT	NT	NT	NT	NT	NT	NT	NT	NT	NT	NT	NT	NT	NT	NT	NT	NT	NT	NT	NT	NT	NT	NT	NT	NT	NT	NT	NT	NT	NT	NT	NT	NT	NT	NT	NT	NT
276	Longikaurin D	NT	NT	NT	NT	NT	NT	2.0 ± 0.11	NT	NT	NT	NT	NT	NT	NT	NT	NT	NT	NT	NT	NT	NT	NT	NT	NT	NT	NT	NT	NT	NT	NT	NT	NT	NT	NT	NT	NT	NT	NT	NT	NT	NT	NT	NT	NT
277	Effusanin D	NT	NT	NT	NT	NT	NT	3.3 ± 0.11	NT	NT	NT	NT	NT	NT	NT	NT	NT	NT	NT	NT	NT	NT	NT	NT	NT	NT	NT	NT	NT	NT	NT	NT	NT	NT	NT	NT	NT	NT	NT	NT	NT	NT	NT	NT	NT
278	Excisanin B	NT	NT	NT	NT	NT	NT	3.3 ± 0.05	NT	NT	NT	NT	NT	NT	NT	NT	NT	NT	NT	NT	NT	NT	NT	NT	NT	NT	NT	NT	NT	NT	NT	NT	NT	NT	NT	NT	NT	NT	NT	NT	NT	NT	NT	NT	NT
279	Lasiokaurin	NT	NT	NT	NT	NT	NT	1.6 ± 0.13	NT	NT	NT	NT	NT	NT	NT	NT	NT	NT	NT	NT	NT	NT	NT	NT	NT	NT	NT	NT	NT	NT	NT	NT	NT	NT	NT	NT	NT	NT	NT	NT	NT	NT	NT	NT	NT
280	Megathyrin A	NT	NT	NT	NT	NT	NT	8.5 ± 0.15	NT	NT	NT	NT	NT	NT	NT	NT	NT	NT	NT	NT	NT	NT	NT	NT	NT	NT	NT	NT	NT	NT	NT	NT	NT	NT	NT	NT	NT	NT	NT	NT	NT	NT	NT	NT	NT
281	Loxothyrin A	NT	NT	NT	NT	NT	NT	24.2 ± 0.25	NT	NT	NT	NT	NT	NT	NT	NT	NT	NT	NT	NT	NT	NT	NT	NT	NT	NT	NT	NT	NT	NT	NT	NT	NT	NT	NT	NT	NT	NT	NT	NT	NT	NT	NT	NT	NT
282	*Ent* -7α,18- diacetoxy-16*β* -hydroxykaurane	NT	NT	NT	NT	NT	NT	NT	NT	NT	NT	NT	NT	NT	NT	NT	NT	NT	NT	NT	NT	NT	NT	NT	NT	NT	NT	NT	NT	NT	NT	NT	NT	NT	NT	NT	NT	NT	NT	NT	NT	NT	NT	NA	NA
283	*Ent* -7*α* -acetoxy,15*α* ,18-dihydroxykaur-16-ene	NT	NT	NT	NT	NT	NT	NT	NT	NT	NT	NT	NT	NT	NT	NT	NT	NT	NT	NT	NT	NT	NT	NT	NT	NT	NT	NT	NT	NT	NT	NT	NT	NT	NT	NT	NT	NT	NT	NT	NT	NT	NT	NT	NT
284	Eubol	NT	NT	NT	NT	NT	NT	NT	NT	NT	NT	NT	NT	NT	NT	NT	NT	NT	NT	NT	NT	NT	NT	NT	NT	NT	NT	NT	NT	NT	NT	NT	NT	NT	NT	NT	NT	NT	NT	NT	NT	NT	NT	NA	23.2 ± 3.2
285	Eubotriol	NT	NT	NT	NT	NT	NT	NT	NT	NT	NT	NT	NT	NT	NT	NT	NT	NT	NT	NT	NT	NT	NT	NT	NT	NT	NT	NT	NT	NT	NT	NT	NT	NT	NT	NT	NT	NT	NT	NT	NT	NT	NT	NA	98.1 ± 2.6
286	Sideroxol	NT	NT	NT	NT	NT	NT	NT	NT	NT	NT	NT	NT	NT	NT	NT	NT	NT	NT	NT	NT	NT	NT	NT	NT	NT	NT	NT	NT	NT	NT	NT	NT	NT	NT	NT	NT	NT	NT	NT	NT	NT	NT	14.5 ± 0.8	25.0 ± 3.0
287	7-*epi* -candicandiol	NT	NT	NT	NT	NT	NT	NT	NT	NT	NT	NT	NT	NT	NT	NT	NT	NT	NT	NT	NT	NT	NT	NT	NT	NT	NT	NT	NT	NT	NT	NT	NT	NT	NT	NT	NT	NT	NT	NT	43.1 ± 0.5	NA	NA	22.8 ± 0.6	21.1 ± 5.1
288	Jungermatrobrunin A	NT	NT	NT	NT	NT	NT	NT	NT	NT	NT	NT	NT	NT	NT	NT	NT	NT	NT	NT	NT	NT	NT	NT	NT	NT	NT	NT	NT	NT	NT	NT	NT	NT	NT	NT	NT	NT	NT	NT	NT	NT	NT	NT	NT
289	1*α* ,6*α* -diacetoxyjungermannenone C	NT	NT	NT	NT	NT	NT	NT	NT	NT	NT	NT	NT	NT	NT	NT	NT	NT	NT	NT	NT	NT	NT	NT	NT	NT	NT	NT	NT	NT	NT	NT	NT	NT	NT	NT	NT	NT	NT	NT	NT	NT	NT	NT	NT
290	1*α* -acetoxy-6*α* -hydroxyjungermannenone C	NT	NT	NT	NT	NT	NT	NT	NT	NT	NT	NT	NT	NT	NT	NT	NT	NT	NT	NT	NT	NT	NT	NT	NT	NT	NT	NT	NT	NT	NT	NT	NT	NT	NT	NT	NT	NT	NT	NT	NT	NT	NT	NT	NT
291	1α,6α-diacetoxy-*ent* -kaura-9(11),16-dien-12,15-dione	NT	NT	NT	NT	NT	NT	NT	NT	NT	NT	NT	NT	NT	NT	NT	NT	NT	NT	NT	NT	NT	NT	NT	NT	NT	NT	NT	NT	NT	NT	NT	NT	NT	NT	NT	NT	NT	NT	NT	NT	NT	NT	NT	NT
292	16*R* -1*α* ,6*β* -Diacetoxy-*ent* -9(11)-kauren-12,15-dione	NT	NT	NT	NT	NT	NT	NT	NT	NT	NT	NT	NT	NT	NT	NT	NT	NT	NT	NT	NT	NT	NT	NT	NT	NT	NT	NT	NT	NT	NT	NT	NT	NT	NT	NT	NT	NT	NT	NT	NT	NT	NT	NT	NT
293	12*β* -hydroxy-1α,6α-diacetoxy-*ent* -kaura-9(11),16-dien-15-one	NT	NT	NT	NT	NT	NT	NT	NT	NT	NT	NT	NT	NT	NT	NT	NT	NT	NT	NT	NT	NT	NT	NT	NT	NT	NT	NT	NT	NT	NT	NT	NT	NT	NT	NT	NT	NT	NT	NT	NT	NT	NT	NT	NT
294	1*α* ,12*β* -dihydroxy-6*α* -acetoxy-*ent* -kaura-9(11),16-dien-15-one	NT	NT	NT	NT	NT	NT	NT	NT	NT	NT	NT	NT	NT	NT	NT	NT	NT	NT	NT	NT	NT	NT	NT	NT	NT	NT	NT	NT	NT	NT	NT	NT	NT	NT	NT	NT	NT	NT	NT	NT	NT	NT	NT	NT
295	6*α* -hydroxy-15*β* -acetoxy-*ent* -kaura-9(11),16-diene	NT	NT	NT	NT	NT	NT	NT	NT	NT	NT	NT	NT	NT	NT	NT	NT	NT	NT	NT	NT	NT	NT	NT	NT	NT	NT	NT	NT	NT	NT	NT	NT	NT	NT	NT	NT	NT	NT	NT	NT	NT	NT	NT	NT
296	6*α* ,15*β* -diacetoxy-*ent* -kaura-9(11),16-diene	NT	NT	NT	NT	NT	NT	NT	NT	NT	NT	NT	NT	NT	NT	NT	NT	NT	NT	NT	NT	NT	NT	NT	NT	NT	NT	NT	NT	NT	NT	NT	NT	NT	NT	NT	NT	NT	NT	NT	NT	NT	NT	NT	NT
297	6*α* ,15*β* -diacetoxy-*ent* -kaura-9(11),16-dien-12-one	NT	NT	NT	NT	NT	NT	NT	NT	NT	NT	NT	NT	NT	NT	NT	NT	NT	NT	NT	NT	NT	NT	NT	NT	NT	NT	NT	NT	NT	NT	NT	NT	NT	NT	NT	NT	NT	NT	NT	NT	NT	NT	NT	NT
298	Nervonin A	NT	NT	NT	NT	NT	NT	NT	NT	NT	NT	NT	NT	NT	NT	NT	NT	NT	NT	NT	NT	NT	NT	NT	NT	NT	NT	NT	NT	NT	NT	NT	NT	NT	NT	NT	NT	NT	NT	NT	NT	NT	NT	NT	NT
299	Nervonin B	NT	NT	NT	NT	NT	NT	NT	1.16	NT	NT	1.53	NT	NT	NT	NT	NT	NT	NT	NT	NT	NT	1.18	NT	NT	NT	NT	NT	NT	NT	NT	NT	NT	NT	NT	NT	NT	NT	NT	NT	NT	NT	NT	NT	NT
300	Nervonin C	NT	NT	NT	NT	NT	NT	NT	NT	NT	NT	NT	NT	NT	NT	NT	NT	NT	NT	NT	NT	NT	NT	NT	NT	NT	NT	NT	NT	NT	NT	NT	NT	NT	NT	NT	NT	NT	NT	NT	NT	NT	NT	NT	NT

301	Nervonin D	NT	NT	NT	NT	NT	NT	NT	NT	NT	NT	NT	NT	NT	NT	NT	NT	NT	NT	NT	NT	NT	NT	NT	NT	NT	NT	NT	NT	NT	NT	NT	NT	NT	NT	NT	NT	NT	NT	NT	NT	NT	NT	NT	NT
302	Nervonin E	NT	NT	NT	NT	NT	NT	NT	NT	NT	NT	NT	NT	NT	NT	NT	NT	NT	NT	NT	NT	NT	NT	NT	NT	NT	NT	NT	NT	NT	NT	NT	NT	NT	NT	NT	NT	NT	NT	NT	NT	NT	NT	NT	NT
303	Nervonin F	NT	NT	NT	NT	NT	NT	NT	NT	NT	NT	NT	NT	NT	NT	NT	NT	NT	NT	NT	NT	NT	NT	NT	NT	NT	NT	NT	NT	NT	NT	NT	NT	NT	NT	NT	NT	NT	NT	NT	NT	NT	NT	NT	NT
304	Nervonin G	NT	NT	NT	NT	NT	NT	NT	NT	NT	NT	NT	NT	NT	NT	NT	NT	NT	NT	NT	NT	NT	NT	NT	NT	NT	NT	NT	NT	NT	NT	NT	NT	NT	NT	NT	NT	NT	NT	NT	NT	NT	NT	NT	NT
305	Nervonin H	NT	NT	NT	NT	NT	NT	NT	NT	NT	NT	NT	NT	NT	NT	NT	NT	NT	NT	NT	NT	NT	NT	NT	NT	NT	NT	NT	NT	NT	NT	NT	NT	NT	NT	NT	NT	NT	NT	NT	NT	NT	NT	NT	NT
306	Nervonin I	NT	NT	NT	NT	NT	NT	NT	NT	NT	NT	NT	NT	NT	NT	NT	NT	NT	NT	NT	NT	NT	NT	NT	NT	NT	NT	NT	NT	NT	NT	NT	NT	NT	NT	NT	NT	NT	NT	NT	NT	NT	NT	NT	NT
307	Nervonin J	NT	NT	NT	NT	NT	NT	NT	NT	NT	NT	NT	NT	NT	NT	NT	NT	NT	NT	NT	NT	NT	NT	NT	NT	NT	NT	NT	NT	NT	NT	NT	NT	NT	NT	NT	NT	NT	NT	NT	NT	NT	NT	NT	NT
308	Calcicolin A	NT	NT	NT	NT	NT	NT	NT	0.51	NT	NT	12.2	NT	NT	NT	NT	NT	NT	NT	NT	NT	NT	2.39	NT	NT	NT	NT	NT	NT	NT	NT	NT	NT	NT	NT	NT	NT	NT	NT	NT	NT	NT	NT	NT	NT
309	Nervosanin	NT	NT	NT	NT	NT	NT	NT	NT	NT	NT	NT	NT	NT	NT	NT	NT	NT	NT	NT	NT	NT	NT	NT	NT	NT	NT	NT	NT	NT	NT	NT	NT	NT	NT	NT	NT	NT	NT	NT	NT	NT	NT	NT	NT
310	Adenanthin K	NT	NT	NT	NT	NT	NT	NT	NT	NT	NT	NT	NT	NT	NT	NT	NT	NT	NT	NT	NT	NT	NT	NT	NT	NT	NT	NT	NT	NT	NT	NT	NT	NT	NT	NT	NT	NT	NT	NT	NT	NT	NT	NT	NT
311	Forrestin B	NT	NT	NT	NT	NT	NT	NT	NT	NT	NT	NT	NT	NT	NT	NT	NT	NT	NT	NT	NT	NT	NT	NT	NT	NT	NT	NT	NT	NT	NT	NT	NT	NT	NT	NT	NT	NT	NT	NT	NT	NT	NT	NT	NT
312	Adenanthin E	NT	NT	NT	NT	NT	NT	NT	NT	NT	NT	NT	NT	NT	NT	NT	NT	NT	NT	NT	NT	NT	NT	NT	NT	NT	NT	NT	NT	NT	NT	NT	NT	NT	NT	NT	NT	NT	NT	NT	NT	NT	NT	NT	NT
313	Weisiensin A	NT	NT	NT	NT	NT	NT	NT	1.43	NT	NT	1.88	NT	NT	NT	NT	NT	NT	NT	NT	NT	NT	4.11	NT	NT	NT	NT	NT	NT	NT	NT	NT	NT	NT	NT	NT	NT	NT	NT	NT	NT	NT	NT	NT	NT
314	Adenanthin	NT	NT	NT	NT	NT	NT	NT	0.32	NT	NT	8.07	NT	NT	NT	NT	NT	NT	NT	NT	NT	NT	1.05	NT	NT	NT	NT	NT	NT	NT	NT	NT	NT	NT	NT	NT	NT	NT	NT	NT	NT	NT	NT	NT	NT
315	Forrestin C	NT	NT	NT	NT	NT	NT	NT	NT	NT	NT	NT	NT	NT	NT	NT	NT	NT	NT	NT	NT	NT	NT	NT	NT	NT	NT	NT	NT	NT	NT	NT	NT	NT	NT	NT	NT	NT	NT	NT	NT	NT	NT	NT	NT
316	Adenanthin J	NT	NT	NT	NT	NT	NT	NT	NT	NT	NT	NT	NT	NT	NT	NT	NT	NT	NT	NT	NT	NT	NT	NT	NT	NT	NT	NT	NT	NT	NT	NT	NT	NT	NT	NT	NT	NT	NT	NT	NT	NT	NT	NT	NT
317	Calcicolin B	NT	NT	NT	NT	NT	NT	NT	0.52	NT	NT	3.84	NT	NT	NT	NT	NT	NT	NT	NT	NT	NT	1.55	NT	NT	NT	NT	NT	NT	NT	NT	NT	NT	NT	NT	NT	NT	NT	NT	NT	NT	NT	NT	NT	NT
318	Albopilosin B	NT	NT	NT	NT	NT	NT	NT	NT	NT	NT	NT	NT	NT	NT	NT	NT	NT	NT	NT	NT	NT	NT	NT	NT	NT	NT	NT	NT	NT	NT	NT	NT	NT	NT	NT	NT	NT	NT	NT	NT	NT	NT	NT	NT
319	Albopilosin C	NT	NT	NT	NT	NT	NT	NT	NT	NT	NT	NT	NT	NT	NT	NT	NT	NT	NT	NT	NT	NT	NT	NT	NT	NT	NT	NT	NT	NT	NT	NT	NT	NT	NT	NT	NT	NT	NT	NT	NT	NT	NT	NT	NT
320	Albopilosin D	NT	NT	NT	NT	NT	NT	NT	NT	NT	NT	NT	NT	NT	NT	NT	NT	NT	NT	NT	NT	NT	NT	NT	NT	NT	NT	NT	NT	NT	NT	NT	NT	NT	NT	NT	NT	NT	NT	NT	NT	NT	NT	NT	NT
321	Albopilosin E	NT	NT	NT	NT	NT	NT	NT	NT	NT	NT	NT	NT	NT	NT	NT	NT	NT	NT	NT	NT	NT	NT	NT	NT	NT	NT	NT	NT	NT	NT	NT	NT	NT	NT	NT	NT	NT	NT	NT	NT	NT	NT	NT	NT
322	Albopilosin F	NT	NT	NT	NT	NT	NT	NT	NT	NT	NT	NT	NT	NT	NT	NT	NT	NT	NT	NT	NT	NT	NT	NT	NT	NT	NT	NT	NT	NT	NT	NT	NT	NT	NT	NT	NT	NT	NT	NT	NT	NT	NT	NT	NT
323	Albopilosin G	NT	NT	NT	NT	NT	NT	NT	NT	NT	NT	NT	NT	NT	NT	NT	NT	NT	NT	NT	NT	NT	NT	NT	NT	NT	NT	NT	NT	NT	NT	NT	NT	NT	NT	NT	NT	NT	NT	NT	NT	NT	NT	NT	NT
324	Albopilosin H	NT	NT	NT	NT	NT	NT	NT	NT	NT	NT	NT	NT	NT	NT	NT	NT	NT	NT	NT	NT	NT	NT	NT	NT	NT	NT	NT	NT	NT	NT	NT	NT	NT	NT	NT	NT	NT	NT	NT	NT	NT	NT	NT	NT
325	Albopilosin I	NT	NT	NT	NT	NT	NT	NT	NT	NT	NT	NT	NT	NT	NT	NT	NT	NT	NT	NT	NT	NT	NT	NT	NT	NT	NT	NT	NT	NT	NT	NT	NT	NT	NT	NT	NT	NT	NT	NT	NT	NT	NT	NT	NT
326	Albopilosin J	NT	NT	NT	NT	NT	NT	NT	NT	NT	NT	NT	NT	NT	NT	NT	NT	NT	NT	NT	NT	NT	NT	NT	NT	NT	NT	NT	NT	NT	NT	NT	NT	NT	NT	NT	NT	NT	NT	NT	NT	NT	NT	NT	NT
327	Albopilosin A	NT	NT	NT	NT	NT	NT	NT	NT	NT	NT	NT	NT	NT	NT	NT	NT	NT	NT	NT	NT	NT	NT	NT	NT	NT	NT	NT	NT	NT	NT	NT	NT	NT	NT	NT	NT	NT	NT	NT	NT	NT	NT	NT	NT
328	Macrocalyxin C	NT	NT	NT	NT	NT	NT	NT	NT	NT	NT	NT	NT	NT	NT	NT	NT	NT	NT	NT	NT	NT	NT	NT	NT	NT	NT	NT	NT	NT	NT	NT	NT	NT	NT	NT	NT	NT	NT	NT	NT	NT	NT	NT	NT
329	Rabdokunmin C	NT	NT	NT	NT	NT	NT	NT	NT	NT	NT	NT	NT	NT	NT	NT	NT	NT	NT	NT	NT	NT	NT	NT	NT	NT	NT	NT	NT	NT	NT	NT	NT	NT	NT	NT	NT	NT	NT	NT	NT	NT	NT	NT	NT
330	Excisanin	NT	NT	NT	NT	NT	NT	NT	NT	NT	NT	NT	NT	NT	NT	NT	NT	NT	NT	NT	NT	NT	NT	NT	NT	NT	NT	NT	NT	NT	NT	NT	NT	NT	NT	NT	NT	NT	NT	NT	NT	NT	NT	NT	NT
331	Amethystonoic acid	NT	NT	NT	NT	NT	NT	NT	NT	NT	NT	NT	NT	NT	NT	NT	NT	NT	NT	NT	NT	NT	NT	NT	NT	NT	NT	NT	NT	NT	NT	NT	NT	NT	NT	NT	NT	NT	NT	NT	NT	NT	NT	NT	NT
332	Coetsanoic acid	NT	NT	NT	NT	NT	NT	NT	NT	NT	NT	NT	NT	NT	NT	NT	NT	NT	NT	NT	NT	NT	NT	NT	NT	NT	NT	NT	NT	NT	NT	NT	NT	NT	NT	NT	NT	NT	NT	NT	NT	NT	NT	NT	NT
333	Isolushinin A	NA	NA	NA	NT	NT	NT	NT	NT	NT	NT	NT	NT	NT	NT	NT	NT	NT	NT	NT	NT	NT	NT	NT	NT	NT	NT	NT	NT	NT	NT	NT	NT	NT	NT	NT	NT	NA	NA	NT	NT	NT	NT	NT	NT
334	Isolushinin B	NA	NA	NA	NT	NT	NT	NT	NT	NT	NT	NT	NT	NT	NT	NT	NT	NT	NT	NT	NT	NT	NT	NT	NT	NT	NT	NT	NT	NT	NT	NT	NT	NT	NT	NT	NT	NA	NA	NT	NT	NT	NT	NT	NT
335	Isolushinin C	NA	NA	NA	NT	NT	NT	NT	NT	NT	NT	NT	NT	NT	NT	NT	NT	NT	NT	NT	NT	NT	NT	NT	NT	NT	NT	NT	NT	NT	NT	NT	NT	NT	NT	NT	NT	NA	NA	NT	NT	NT	NT	NT	NT
336	Isolushinin D	1.1	4.7	6.4	NT	NT	NT	NT	NT	NT	NT	NT	NT	NT	NT	NT	NT	NT	NT	NT	NT	NT	NT	NT	NT	NT	NT	NT	NT	NT	NT	NT	NT	NT	NT	NT	NT	7	6.4	NT	NT	NT	NT	NT	NT
337	Isolushinin E	NA	NA	NA	NT	NT	NT	NT	NT	NT	NT	NT	NT	NT	NT	NT	NT	NT	NT	NT	NT	NT	NT	NT	NT	NT	NT	NT	NT	NT	NT	NT	NT	NT	NT	NT	NT	NA	NA	NT	NT	NT	NT	NT	NT
338	Isolushinin F	NA	NA	NA	NT	NT	NT	NT	NT	NT	NT	NT	NT	NT	NT	NT	NT	NT	NT	NT	NT	NT	NT	NT	NT	NT	NT	NT	NT	NT	NT	NT	NT	NT	NT	NT	NT	NA	NA	NT	NT	NT	NT	NT	NT
339	Isolushinin G	NA	NA	NA	NT	NT	NT	NT	NT	NT	NT	NT	NT	NT	NT	NT	NT	NT	NT	NT	NT	NT	NT	NT	NT	NT	NT	NT	NT	NT	NT	NT	NT	NT	NT	NT	NT	NA	NA	NT	NT	NT	NT	NT	NT
340	Isolushinin H	2.5	>10	>10	NT	NT	NT	NT	NT	NT	NT	NT	NT	NT	NT	NT	NT	NT	NT	NT	NT	NT	NT	NT	NT	NT	NT	NT	NT	NT	NT	NT	NT	NT	NT	NT	NT	6.6	6.1	NT	NT	NT	NT	NT	NT
341	Isolushinin I	NA	NA	NA	NT	NT	NT	NT	NT	NT	NT	NT	NT	NT	NT	NT	NT	NT	NT	NT	NT	NT	NT	NT	NT	NT	NT	NT	NT	NT	NT	NT	NT	NT	NT	NT	NT	NA	NA	NT	NT	NT	NT	NT	NT
342	Isolushinin J	NA	NA	NA	NT	NT	NT	NT	NT	NT	NT	NT	NT	NT	NT	NT	NT	NT	NT	NT	NT	NT	NT	NT	NT	NT	NT	NT	NT	NT	NT	NT	NT	NT	NT	NT	NT	NA	NA	NT	NT	NT	NT	NT	NT
343	(20S)-11β ,14β,20 -trihydroxy-7α,20-epoxy-ent-kaur-16-en-15-one	1.2	6.2	>10	NT	NT	NT	NT	NT	NT	NT	NT	NT	NT	NT	NT	NT	NT	NT	NT	NT	NT	NT	NT	NT	NT	NT	NT	NT	NT	NT	NT	NT	NT	NT	NT	NT	8.6	4.4	NT	NT	NT	NT	NT	NT
344	(20S)-11β ,14β -dihydroxy-20-methoxy-7α,20-epoxy-ent-kaur-16-en-15-one	0.95	4.4	5.9	NT	NT	NT	NT	NT	NT	NT	NT	NT	NT	NT	NT	NT	NT	NT	NT	NT	NT	NT	NT	NT	NT	NT	NT	NT	NT	NT	NT	NT	NT	NT	NT	NT	9.8	3.3	NT	NT	NT	NT	NT	NT
345	(20S)-11β ,14β -dihydroxy-20-ethoxy-7α,20-epoxy-ent-kaur-16-en-15-one	0.64	3.5	5.6	NT	NT	NT	NT	NT	NT	NT	NT	NT	NT	NT	NT	NT	NT	NT	NT	NT	NT	NT	NT	NT	NT	NT	NT	NT	NT	NT	NT	NT	NT	NT	NT	NT	5.2	2.4	NT	NT	NT	NT	NT	NT
346	Flexicalin A	0.64	9.9	4.2	NT	NT	NT	NT	NT	NT	NT	NT	NT	NT	NT	NT	NT	NT	NT	NT	NT	NT	NT	NT	NT	NT	NT	NT	NT	NT	NT	NT	NT	NT	NT	NT	NT	5.7	>10	NT	NT	NT	NT	NT	NT
347	Kamebakaurinin	1.8	>10	9.8	NT	NT	NT	NT	NT	NT	NT	NT	NT	NT	NT	NT	NT	NT	NT	NT	NT	NT	NT	NT	NT	NT	NT	NT	NT	NT	NT	NT	NT	NT	NT	NT	NT	>10	3.3	NT	NT	NT	NT	NT	NT
348	Phyllostachyisn F	0.85	5.5	>10	NT	NT	NT	NT	NT	NT	NT	NT	NT	NT	NT	NT	NT	NT	NT	NT	NT	NT	NT	NT	NT	NT	NT	NT	NT	NT	NT	NT	NT	NT	NT	NT	NT	4.5	>10	NT	NT	NT	NT	NT	NT
349	Phyllostachyisn H	6.4	>10	3.3	NT	NT	NT	NT	NT	NT	NT	NT	NT	NT	NT	NT	NT	NT	NT	NT	NT	NT	NT	NT	NT	NT	NT	NT	NT	NT	NT	NT	NT	NT	NT	NT	NT	>10	>10	NT	NT	NT	NT	NT	NT
350	Oridonin	3.9	>10	>10	NT	NT	NT	NT	NT	NT	NT	NT	NT	NT	NT	NT	NT	NT	NT	NT	NT	NT	NT	NT	NT	NT	NT	NT	NT	NT	NT	NT	NT	NT	NT	NT	NT	>10	>10	NT	NT	NT	NT	NT	NT
351	Enmenol	6.7	>10	>10	NT	NT	NT	NT	NT	NT	NT	NT	NT	NT	NT	NT	NT	NT	NT	NT	NT	NT	NT	NT	NT	NT	NT	NT	NT	NT	NT	NT	NT	NT	NT	NT	NT	>10	>10	NT	NT	NT	NT	NT	NT
352	ludognin A	3.1	>10	>10	NT	NT	NT	NT	NT	NT	NT	NT	NT	NT	NT	NT	NT	NT	NT	NT	NT	NT	NT	NT	NT	NT	NT	NT	NT	NT	NT	NT	NT	NT	NT	NT	NT	>10	>10	NT	NT	NT	NT	NT	NT
353	Iudongnin G	NA	NA	NA	NT	NT	NT	NT	NT	NT	NT	NT	NT	NT	NT	NT	NT	NT	NT	NT	NT	NT	NT	NT	NT	NT	NT	NT	NT	NT	NT	NT	NT	NT	NT	NT	NT	NA	NA	NT	NT	NT	NT	NT	NT
354	Guidongnin E	NA	NA	NA	NT	NT	NT	NT	NT	NT	NT	NT	NT	NT	NT	NT	NT	NT	NT	NT	NT	NT	NT	NT	NT	NT	NT	NT	NT	NT	NT	NT	NT	NT	NT	NT	NT	NA	NA	NT	NT	NT	NT	NT	NT
355	Guidongnin F	NA	NA	NA	NT	NT	NT	NT	NT	NT	NT	NT	NT	NT	NT	NT	NT	NT	NT	NT	NT	NT	NT	NT	NT	NT	NT	NT	NT	NT	NT	NT	NT	NT	NT	NT	NT	NA	NA	NT	NT	NT	NT	NT	NT
356	Sculponeatin B	NA	NA	NA	NT	NT	NT	NT	NT	NT	NT	NT	NT	NT	NT	NT	NT	NT	NT	NT	NT	NT	NT	NT	NT	NT	NT	NT	NT	NT	NT	NT	NT	NT	NT	NT	NT	NA	NA	NT	NT	NT	NT	NT	NT
357	3β ,7α ,12α ,14β - tetrahydroxy-*ent* -kaur-16-en-15-one.	NT	NT	NT	NA	NT	NT	NT	NT	NT	NT	NA	NT	NA	NT	NT	NT	NT	NT	NT	NT	NT	NT	NA	NT	NT	NT	NT	NT	NT	NT	NT	NT	NT	NT	NT	NA	NT	NT	NT	NT	NT	NT	NT	NT
358	3α ,7α ,14β -trihydroxy-12α -acetoxy-*ent* -kaur-16-en15-one.	NT	NT	NT	>10	NT	NT	NT	NT	NT	NT	>10	NT	>10	NT	NT	NT	NT	NT	NT	NT	NT	NT	8.12	NT	NT	NT	NT	NT	NT	NT	NT	NT	NT	NT	NT	8.32	NT	NT	NT	NT	NT	NT	NT	NT
359	14β - hydroxy-3β ,7α -diacetoxy-*ent* -kaur-16-en-12,15-dione	NT	NT	NT	NA	NT	NT	NT	NT	NT	NT	NA	NT	NA	NT	NT	NT	NT	NT	NT	NT	NT	NT	NA	NT	NT	NT	NT	NT	NT	NT	NT	NT	NT	NT	NT	NA	NT	NT	NT	NT	NT	NT	NT	NT
360	3β ,14β ,15β -trihydroxy-*ent* -kaur-16-en-12-one.	NT	NT	NT	NT	NT	NT	NT	NT	NT	NT	NT	NT	NT	NT	NT	NT	NT	NT	NT	NT	NT	NT	NT	NT	NT	NT	NT	NT	NT	NT	NT	NT	NT	NT	NT	NT	NT	NT	NT	NT	NT	NT	NT	NT
361	3β ,12α ,15β -trihydroxy-14β -acetoxyent-kaur-16-ene	NT	NT	NT	NA	NT	NT	NT	NT	NT	NT	NA	NT	NA	NT	NT	NT	NT	NT	NT	NT	NT	NT	NA	NT	NT	NT	NT	NT	NT	NT	NT	NT	NT	NT	NT	NA	NT	NT	NT	NT	NT	NT	NT	NT
362	3α ,7α ,14β ,15β -tetrahydroxy-*ent* -kaur-16-en-12-one	NT	NT	NT	NT	NT	NT	NT	NT	NT	NT	NT	NT	NT	NT	NT	NT	NT	NT	NT	NT	NT	NT	NT	NT	NT	NT	NT	NT	NT	NT	NT	NT	NT	NT	NT	NT	NT	NT	NT	NT	NT	NT	NT	NT
363	1α ,7α ,12α ,14β -tetrahydroxy-3β -acetoxy-*ent* -kaur-16-en-15-one	NT	NT	NT	NA	NT	NT	NT	NT	NT	NT	NA	NT	NA	NT	NT	NT	NT	NT	NT	NT	NT	NT	NA	NT	NT	NT	NT	NT	NT	NT	NT	NT	NT	NT	NT	NT	NT	NT	NT	NT	NT	NT	NT	NT
364	1α ,7α ,14β ,15β -tetrahydroxy-3β -acetoxy-*ent* -kaur-16-en-12-one	NT	NT	NT	NA	NT	NT	NT	NT	NT	NT	NA	NT	NA	NT	NT	NT	NT	NT	NT	NT	NT	NT	NA	NT	NT	NT	NT	NT	NT	NT	NT	NT	NT	NT	NT	NA	NT	NT	NT	NT	NT	NT	NT	NT
365	16(*R*)-7α ,14β -dihydroxy-17-methoxy-3β - acetoxy-*ent* -kaur-12,15-dione	NT	NT	NT	>10	NT	NT	NT	NT	NT	NT	>10	NT	7.42	NT	NT	NT	NT	NT	NT	NT	NT	NT	>10	NT	NT	NT	NT	NT	NT	NT	NT	NT	NT	NT	NT	8.74	NT	NT	NT	NT	NT	NT	NT	NT
366	3α ,7α ,12α ,14β -tetrahydroxy-*ent* -kaur-16-en15-one 7,14-acetonide	NT	NT	NT	>10	NT	NT	NT	NT	NT	NT	6.02	NT	>10	NT	NT	NT	NT	NT	NT	NT	NT	NT	>10	NT	NT	NT	NT	NT	NT	NT	NT	NT	NT	NT	NT	3.56	NT	NT	NT	NT	NT	NT	NT	NT
367	3β ,7α ,12α ,14β - tetrahydroxy-16-*ent* -kaur-15-oe-7,14-acetonide	NT	NT	NT	NT	NT	NT	NT	NT	NT	NT	NT	NT	NT	NT	NT	NT	NT	NT	NT	NT	NT	NT	NT	NT	NT	NT	NT	NT	NT	NT	NT	NT	NT	NT	NT	NT	NT	NT	NT	NT	NT	NT	NT	NT
368	1α ,7α ,12α ,14β - tetrahydroxy-3β -acetoxy-*ent* -kaur-16-en-15-one 7,14-acetonide	NT	NT	NT	NT	NT	NT	NT	NT	NT	NT	NT	NT	NT	NT	NT	NT	NT	NT	NT	NT	NT	NT	NT	NT	NT	NT	NT	NT	NT	NT	NT	NT	NT	NT	NT	NT	NT	NT	NT	NT	NT	NT	NT	NT
369	3α ,7α ,12α ,14β -tetrahydroxy-*ent* -kaur-16-en-15-one 7,14-acetal	NT	NT	NT	NT	NT	NT	NT	NT	NT	NT	NT	NT	NT	NT	NT	NT	NT	NT	NT	NT	NT	NT	NT	NT	NT	NT	NT	NT	NT	NT	NT	NT	NT	NT	NT	NT	NT	NT	NT	NT	NT	NT	NT	NT
370	Pseurata A	NT	NT	NT	9.05	NT	NT	NT	NT	NT	NT	2.92	NT	>10	NT	NT	NT	NT	NT	NT	NT	NT	NT	>10	NT	NT	NT	NT	NT	NT	NT	NT	NT	NT	NT	NT	7.69	NT	NT	NT	NT	NT	NT	NT	NT
371	Pseurata B	NT	NT	NT	NA	NT	NT	NT	NT	NT	NT	NA	NT	NA	NT	NT	NT	NT	NT	NT	NT	NT	NT	NA	NT	NT	NT	NT	NT	NT	NT	NT	NT	NT	NT	NT	NA	NT	NT	NT	NT	NT	NT	NT	NT
372	Pseurata C	NT	NT	NT	>10	NT	NT	NT	NT	NT	NT	7.62	NT	>10	NT	NT	NT	NT	NT	NT	NT	NT	NT	>10	NT	NT	NT	NT	NT	NT	NT	NT	NT	NT	NT	NT	2.08	NT	NT	NT	NT	NT	NT	NT	NT
373	Pseurata F	NT	NT	NT	NA	NT	NT	NT	NT	NT	NT	NA	NT	NA	NT	NT	NT	NT	NT	NT	NT	NT	NT	NA	NT	NT	NT	NT	NT	NT	NT	NT	NT	NT	NT	NT	NA	NT	NT	NT	NT	NT	NT	NT	NT
374	Glaucocalyxin A	NT	NT	NT	8.73	NT	NT	NT	NT	NT	NT	5.39	NT	>10	NT	NT	NT	NT	NT	NT	NT	NT	NT	>10	NT	NT	NT	NT	NT	NT	NT	NT	NT	NT	NT	NT	2.9	NT	NT	NT	NT	NT	NT	NT	NT
375	Glaucocalyxin B	NT	NT	NT	>10	NT	NT	NT	NT	NT	NT	6.22	NT	>10	NT	NT	NT	NT	NT	NT	NT	NT	NT	>10	NT	NT	NT	NT	NT	NT	NT	NT	NT	NT	NT	NT	7.29	NT	NT	NT	NT	NT	NT	NT	NT
376	Isodomedin	NT	NT	NT	>10	NT	NT	NT	NT	NT	NT	>10	NT	>10	NT	NT	NT	NT	NT	NT	NT	NT	NT	>10	NT	NT	NT	NT	NT	NT	NT	NT	NT	NT	NT	NT	NA	NT	NT	NT	NT	NT	NT	NT	NT
377	Minheryin G	NT	NT	NT	NA	NT	NT	NT	NT	NT	NT	NA	NT	NA	NT	NT	NT	NT	NT	NT	NT	NT	NT	NA	NT	NT	NT	NT	NT	NT	NT	NT	NT	NT	NT	NT	NT	NT	NT	NT	NT	NT	NT	NT	NT
378	Kamebanin	NT	NT	NT	NT	NT	NT	NT	NT	NT	NT	NT	NT	NT	NT	NT	NT	NT	NT	NT	NT	NT	NT	NT	NT	NT	NT	NT	NT	NT	NT	NT	NT	NT	NT	NT	9.02	NT	NT	NT	NT	NT	NT	NT	NT
379	Wangzaozin A	NT	NT	NT	5.8	NT	NT	NT	NT	NT	NT	9.29	NT	>10	NT	NT	NT	NT	NT	NT	NT	NT	NT	>10	NT	NT	NT	NT	NT	NT	NT	NT	NT	NT	NT	NT	4	NT	NT	NT	NT	NT	NT	NT	NT
380	Leukamenin E	NT	NT	NT	>10	NT	NT	NT	NT	NT	NT	>10	NT	>10	NT	NT	NT	NT	NT	NT	NT	NT	NT	>10	NT	NT	NT	NT	NT	NT	NT	NT	NT	NT	NT	NT	NT	NT	NT	NT	NT	NT	NT	NT	NT
381	Dihydropseurata F	NT	NT	NT	NT	NT	NT	NT	NT	NT	NT	NT	NT	NT	NT	NT	NT	NT	NT	NT	NT	NT	NT	NT	NT	NT	NT	NT	NT	NT	NT	NT	NT	NT	NT	NT	NT	NT	NT	NT	NT	NT	NT	NT	NT
382	Sculponeatin N	NT	NT	NT	NT	NT	NT	NT	0.29 ± 0.0	NT	NT	NT	NT	NT	NT	NT	NT	NT	NT	NT	NT	NT	0.21 ± 0.0	NT	NT	NT	NT	NT	NT	NT	NT	NT	NT	NT	NT	NT	NT	NT	NT	NT	NT	NT	NT	NT	NT
383	Sculponeatin O	NT	NT	NT	NT	NT	NT	NT	0.39 ± 0.0	NT	NT	NT	NT	NT	NT	NT	NT	NT	NT	NT	NT	NT	0.34 ± 0.0	NT	NT	NT	NT	NT	NT	NT	NT	NT	NT	NT	NT	NT	NT	NT	NT	NT	NT	NT	NT	NT	NT
384	Sculponeatin P	NT	NT	NT	NT	NT	NT	NT	>100	NT	NT	NT	NT	NT	NT	NT	NT	NT	NT	NT	NT	NT	>100	NT	NT	NT	NT	NT	NT	NT	NT	NT	NT	NT	NT	NT	NT	NT	NT	NT	NT	NT	NT	NT	NT
385	Sculponeatin Q	NT	NT	NT	NT	NT	NT	NT	>100	NT	NT	NT	NT	NT	NT	NT	NT	NT	NT	NT	NT	NT	>100	NT	NT	NT	NT	NT	NT	NT	NT	NT	NT	NT	NT	NT	NT	NT	NT	NT	NT	NT	NT	NT	NT
386	Sculponeatin R	NT	NT	NT	NT	NT	NT	NT	20.46 ± 0.	NT	NT	NT	NT	NT	NT	NT	NT	NT	NT	NT	NT	NT	2.63 ± 0.2	NT	NT	NT	NT	NT	NT	NT	NT	NT	NT	NT	NT	NT	NT	NT	NT	NT	NT	NT	NT	NT	NT
387	Sculponeatin S	NT	NT	NT	NT	NT	NT	NT	>100	NT	NT	NT	NT	NT	NT	NT	NT	NT	NT	NT	NT	NT	>100	NT	NT	NT	NT	NT	NT	NT	NT	NT	NT	NT	NT	NT	NT	NT	NT	NT	NT	NT	NT	NT	NT
388	Sculponeatin C	NT	NT	NT	NT	NT	NT	NT	0.33 ± 0.0	NT	NT	NT	NT	NT	NT	NT	NT	NT	NT	NT	NT	NT	0.29 ± 0.0	NT	NT	NT	NT	NT	NT	NT	NT	NT	NT	NT	NT	NT	NT	NT	NT	NT	NT	NT	NT	NT	NT
389	Diol	NT	NT	NT	NT	NT	NT	NT	>100	NT	NT	NT	NT	NT	NT	NT	NT	NT	NT	NT	NT	NT	>100	NT	NT	NT	NT	NT	NT	NT	NT	NT	NT	NT	NT	NT	NT	NT	NT	NT	NT	NT	NT	NT	NT
390	Macrocalyxoformin E	NT	NT	NT	NT	NT	NT	NT	>100	NT	NT	NT	NT	NT	NT	NT	NT	NT	NT	NT	NT	NT	>100	NT	NT	NT	NT	NT	NT	NT	NT	NT	NT	NT	NT	NT	NT	NT	NT	NT	NT	NT	NT	NT	NT
391	Epinodosin	NT	NT	NT	NT	NT	NT	NT	0.45 ± 0.0	NT	NT	NT	NT	NT	NT	NT	NT	NT	NT	NT	NT	NT	0.32 ± 0.0	NT	NT	NT	NT	NT	NT	NT	NT	NT	NT	NT	NT	NT	NT	NT	NT	NT	NT	NT	NT	NT	NT
392	Enmein	NT	NT	NT	NT	NT	NT	NT	22.09 ± 0.	NT	NT	NT	NT	NT	NT	NT	NT	NT	NT	NT	NT	NT	0.59 ± 0.0	NT	NT	NT	NT	NT	NT	NT	NT	NT	NT	NT	NT	NT	NT	NT	NT	NT	NT	NT	NT	NT	NT
393	Isodocarpin	NT	NT	NT	NT	NT	NT	NT	2.43 ± 0.2	NT	NT	NT	NT	NT	NT	NT	NT	NT	NT	NT	NT	NT	1.02 ± 0.0	NT	NT	NT	NT	NT	NT	NT	NT	NT	NT	NT	NT	NT	NT	NT	NT	NT	NT	NT	NT	NT	NT
394	Nodosin	NT	NT	NT	NT	NT	NT	NT	2.55 ± 0.2	NT	NT	NT	NT	NT	NT	NT	NT	NT	NT	NT	NT	NT	0.58 ± 0.0	NT	NT	NT	NT	NT	NT	NT	NT	NT	NT	NT	NT	NT	NT	NT	NT	NT	NT	NT	NT	NT	NT
395	Longirabdolide C	NT	NT	NT	NT	NT	NT	NT	>100	NT	NT	NT	NT	NT	NT	NT	NT	NT	NT	NT	NT	NT	3.98 ± 0.2	NT	NT	NT	NT	NT	NT	NT	NT	NT	NT	NT	NT	NT	NT	NT	NT	NT	NT	NT	NT	NT	NT
396	Macrocalyxoformin B	NT	NT	NT	NT	NT	NT	NT	20.89 ± 0.	NT	NT	NT	NT	NT	NT	NT	NT	NT	NT	NT	NT	NT	0.89 ± 0.0	NT	NT	NT	NT	NT	NT	NT	NT	NT	NT	NT	NT	NT	NT	NT	NT	NT	NT	NT	NT	NT	NT
397	Sculponeatin A	NT	NT	NT	NT	NT	NT	NT	0.31 ± 0.0	NT	NT	NT	NT	NT	NT	NT	NT	NT	NT	NT	NT	NT	0.23 ± 0.0	NT	NT	NT	NT	NT	NT	NT	NT	NT	NT	NT	NT	NT	NT	NT	NT	NT	NT	NT	NT	NT	NT
398	Sculponeatin B	NT	NT	NT	NT	NT	NT	NT	>100	NT	NT	NT	NT	NT	NT	NT	NT	NT	NT	NT	NT	NT	>100	NT	NT	NT	NT	NT	NT	NT	NT	NT	NT	NT	NT	NT	NT	NT	NT	NT	NT	NT	NT	NT	NT
399	Xerophilusin N	NT	NT	NT	NT	NT	NT	NT	NT	NT	NT	NT	NT	NT	NT	NT	NT	NT	NT	NT	NT	NT	NT	NT	NT	NT	NT	NT	NT	NT	NT	NT	NT	NT	NT	NT	NT	NT	NT	NT	NT	NT	NT	NT	NT
400	Laxiflorin J	NT	NT	49.054	NT	NT	NT	NT	NT	NT	NT	NT	NT	NT	NT	NT	NT	NT	NT	NT	NT	0.314	0.473	NT	NT	NT	NT	NT	NT	NT	NT	NT	NT	NT	NT	NT	NT	NT	NT	NT	NT	NT	NT	NT	NT

401	Laxiflorin K	NT	NT	NT	NT	NT	NT	NT	NT	NT	NT	NT	NT	NT	NT	NT	NT	NT	NT	NT	NT	1753.166	11.874	NT	NT	NT	NT	NT	NT	NT	NT	NT	NT	NT	NT	NT	NT	NT	NT	NT	NT	NT	NT	NT	NT
402	Laxiflorin L	NT	NT	24.659	NT	NT	NT	NT	NT	NT	NT	NT	NT	NT	NT	NT	NT	NT	NT	NT	NT	5.162	1.126	NT	NT	NT	NT	NT	NT	NT	NT	NT	NT	NT	NT	NT	NT	NT	NT	NT	NT	NT	NT	NT	NT
403	Laxiflorin M	NT	NT	NT	NT	NT	NT	NT	NT	NT	NT	NT	NT	NT	NT	NT	NT	NT	NT	NT	NT	NT	218.685	NT	NT	NT	NT	NT	NT	NT	NT	NT	NT	NT	NT	NT	NT	NT	NT	NT	NT	NT	NT	NT	NT
404	Laxiflorin N	NT	NT	NT	NT	NT	NT	NT	NT	NT	NT	NT	NT	NT	NT	NT	NT	NT	NT	NT	NT	NT	NT	NT	NT	NT	NT	NT	NT	NT	NT	NT	NT	NT	NT	NT	NT	NT	NT	NT	NT	NT	NT	NT	NT
405	maoecrystal A	NT	NT	64.494	NT	NT	NT	NT	NT	NT	NT	NT	NT	NT	NT	NT	NT	NT	NT	NT	NT	0.051	0.132	NT	NT	NT	NT	NT	NT	NT	NT	NT	NT	NT	NT	NT	NT	NT	NT	NT	NT	NT	NT	NT	NT
406	Phyllostacin F	NT	NT	NT	NT	NT	NT	NT	NT	NT	NT	NT	NT	NT	NT	NT	NT	NT	NT	NT	NT	NT	NT	NT	NT	NT	NT	NT	NT	NT	NT	NT	NT	NT	NT	NT	NT	NT	NT	NT	NT	NT	NT	NT	NT
407	Rosthorin A	NT	NT	NT	NT	NT	NT	NT	NT	NT	NT	NT	NT	NT	NT	NT	NT	NT	NT	NT	NT	NT	NT	NT	NT	NT	NT	NT	NT	NT	NT	NT	NT	NT	NT	NT	NT	NT	NT	NT	NT	NT	NT	NT	NT
408	Rabdoternin C	NT	NT	NT	NT	NT	NT	NT	NT	NT	NT	NT	NT	NT	NT	NT	NT	NT	NT	NT	NT	NT	NT	NT	NT	NT	NT	NT	NT	NT	NT	NT	NT	NT	NT	NT	NT	NT	NT	NT	NT	NT	NT	NT	NT
409	Enmenol	NT	NT	NT	NT	NT	NT	NT	NT	NT	NT	NT	NT	NT	NT	NT	NT	NT	NT	NT	NT	NT	NT	NT	NT	NT	NT	NT	NT	NT	NT	NT	NT	NT	NT	NT	NT	NT	NT	NT	NT	NT	NT	NT	NT
410	Oridonin	NT	NT	NT	NT	NT	NT	NT	NT	NT	NT	NT	NT	NT	NT	NT	NT	NT	NT	NT	NT	NT	NT	NT	NT	NT	NT	NT	NT	NT	NT	NT	NT	NT	NT	NT	NT	NT	NT	NT	NT	NT	NT	NT	NT
411	Lasiocarpanin	NT	NT	NT	NT	NT	NT	NT	NT	NT	NT	NT	NT	NT	NT	NT	NT	NT	NT	NT	NT	NT	NT	NT	NT	NT	NT	NT	NT	NT	NT	NT	NT	NT	NT	NT	NT	NT	NT	NT	NT	NT	NT	NT	NT
412	Sculponeatin C	NT	NT	NT	NT	NT	NT	NT	NT	NT	NT	NT	NT	NT	NT	NT	NT	NT	NT	NT	NT	NT	NT	NT	NT	NT	NT	NT	NT	NT	NT	NT	NT	NT	NT	NT	NT	NT	NT	NT	NT	NT	NT	NT	NT
413	Phyllostacin H	NT	NT	NT	NT	NT	NT	NT	NT	NT	NT	NT	NT	NT	NT	NT	NT	NT	NT	NT	NT	NT	NT	NT	NT	NT	NT	NT	NT	NT	NT	NT	NT	NT	NT	NT	NT	NT	NT	NT	NT	NT	NT	NT	NT
414	Phyllostacin I	NT	NT	NT	NT	NT	NT	NT	NT	NT	NT	NT	NT	NT	NT	NT	NT	NT	NT	NT	NT	NT	NT	NT	NT	NT	NT	NT	NT	NT	NT	NT	NT	NT	NT	NT	NT	NT	NT	NT	NT	NT	NT	NT	NT
415	Xerophilusin B	NT	NT	NT	NT	NT	NT	NT	NT	NT	NT	NT	NT	NT	NT	NT	NT	NT	NT	NT	NT	NT	NT	NT	NT	NT	NT	NT	NT	NT	NT	NT	NT	NT	NT	NT	NT	NT	NT	NT	NT	NT	NT	NT	NT
416	Ponicidin	NT	NT	NT	NT	NT	NT	NT	NT	NT	NT	NT	NT	NT	NT	NT	NT	NT	NT	NT	NT	NT	NT	NT	NT	NT	NT	NT	NT	NT	NT	NT	NT	NT	NT	NT	NT	NT	NT	NT	NT	NT	NT	NT	NT
417	Macrocalin B	NT	NT	NT	NT	NT	NT	NT	NT	NT	NT	NT	NT	NT	NT	NT	NT	NT	NT	NT	NT	NT	NT	NT	NT	NT	NT	NT	NT	NT	NT	NT	NT	NT	NT	NT	NT	NT	NT	NT	NT	NT	NT	NT	NT
418	Phyllostacin G	NT	NT	NT	NT	NT	NT	NT	NT	NT	NT	NT	NT	NT	NT	NT	NT	NT	NT	NT	NT	NT	NT	NT	NT	NT	NT	NT	NT	NT	NT	NT	NT	NT	NT	NT	NT	NT	NT	NT	NT	NT	NT	NT	NT
419	Phyllostachysin A	NT	NT	NT	NT	NT	NT	NT	NT	NT	NT	NT	NT	NT	NT	NT	NT	NT	NT	NT	NT	NT	NT	NT	NT	NT	NT	NT	NT	NT	NT	NT	NT	NT	NT	NT	NT	NT	NT	NT	NT	NT	NT	NT	NT
420	Macrocalyxoformin E	NT	NT	NT	NT	NT	NT	NT	NT	NT	NT	NT	NT	NT	NT	NT	NT	NT	NT	NT	NT	NT	NT	NT	NT	NT	NT	NT	NT	NT	NT	NT	NT	NT	NT	NT	NT	NT	NT	NT	NT	NT	NT	NT	NT
421	6β,13α,15β-trihydroxy-16-ene-3α,20-epoxy-*ent*-kaur-1,7-dione	NA	NA	NT	NA	NA	NT	NT	NT	NT	NT	NA	NT	NT	NT	NT	NT	NT	NT	NT	NT	NT	NT	NT	NT	NT	NT	NT	NT	NT	NT	NT	NT	NT	NT	NT	NT	NT	NT	NT	NT	NT	NT	NT	NT
422	6-hydroxy-3α,20-epoxy-5(6)-ene-*ent*-kaur-1,7,15-trione	NA	NA	NT	NA	NA	NT	NT	NT	NT	NT	NA	NT	NT	NT	NT	NT	NT	NT	NT	NT	NT	NT	NT	NT	NT	NT	NT	NT	NT	NT	NT	NT	NT	NT	NT	NT	NT	NT	NT	NT	NT	NT	NT	NT
423	6-hydroxy-15β-acetoxy-3α,20-epoxy-16β,17-epoxy5(6)-ene-*ent*-kaur-1,7-dione	NA	NA	NT	NA	NA	NT	NT	NT	NT	NT	NA	NT	NT	NT	NT	NT	NT	NT	NT	NT	NT	NT	NT	NT	NT	NT	NT	NT	NT	NT	NT	NT	NT	NT	NT	NT	NT	NT	NT	NT	NT	NT	NT	NT
424	3α,17-dihydroxy-15(16)-ene-*ent*-kaur-7-one	NA	NA	NT	NA	NA	NT	NT	NT	NT	NT	NA	NT	NT	NT	NT	NT	NT	NT	NT	NT	NT	NT	NT	NT	NT	NT	NT	NT	NT	NT	NT	NT	NT	NT	NT	NT	NT	NT	NT	NT	NT	NT	NT	NT
425	Laxiflorin M	NA	NA	NT	NA	NA	NT	NT	NT	NT	NT	NA	NT	NT	NT	NT	NT	NT	NT	NT	NT	NT	NT	NT	NT	NT	NT	NT	NT	NT	NT	NT	NT	NT	NT	NT	NT	NT	NT	NT	NT	NT	NT	NT	NT
426	Eriocalyxin C	NA	NA	NT	NA	NA	NT	NT	NT	NT	NT	NA	NT	NT	NT	NT	NT	NT	NT	NT	NT	NT	NT	NT	NT	NT	NT	NT	NT	NT	NT	NT	NT	NT	NT	NT	NT	NT	NT	NT	NT	NT	NT	NT	NT
427	Maoecrystal A	NA	NA	NT	NA	NA	NT	NT	NT	NT	NT	NA	NT	NT	NT	NT	NT	NT	NT	NT	NT	NT	NT	NT	NT	NT	NT	NT	NT	NT	NT	NT	NT	NT	NT	NT	NT	NT	NT	NT	NT	NT	NT	NT	NT
428	Maoecrystal U	4.6	16.2	NT		8.53	6.41	NT	NT	NT	NT	>40	NT	NT	NT	NT	NT	NT	NT	NT	NT	NT	NT	NT	NT	NT	NT	NT	NT	NT	NT	NT	NT	NT	NT	NT	NT	NT	NT	NT	NT	NT	NT	NT	NT
429	Maoecrystal P	NA	NA	NT	NA	NA	NT	NT	NT	NT	NT	NA	NT	NT	NT	NT	NT	NT	NT	NT	NT	NT	NT	NT	NT	NT	NT	NT	NT	NT	NT	NT	NT	NT	NT	NT	NT	NT	NT	NT	NT	NT	NT	NT	NT
577	Enmedol	NT	NT	NT	NT	NT	NT	NT	NT	NT	NT	NT	NT	NT	NT	NT	NT	NT	NT	NT	NT	NT	NT	NT	NT	NT	NT	NT	NT	NT	NT	NT	NT	NT	NT	NT	NT			NT	NT	NT	NT	NT	NT
431	Xindongnin C	NT	NT	NT	NT	NT	NT	NT	NT	NT	NT	NT	NT	NT	NT	NT	NT	NT	NT	NT	NT	NT	5.6	NT	NT	NT	NT	NT	NT	NT	NT	NT	NT	NT	NT	NT	NT	NT	NT	NT	NT	NT	NT	NT	NT
432	Xindongnin D	NT	NT	NT	NT	NT	NT	NT	NT	NT	NT	NT	NT	NT	NT	NT	NT	NT	NT	NT	NT	NT	NA	NT	NT	NT	NT	NT	NT	NT	NT	NT	NT	NT	NT	NT	NT	NT	NT	NT	NT	NT	NT	NT	NT
433	Xindongnin E	NT	NT	NT	NT	NT	NT	NT	NT	NT	NT	NT	NT	NT	NT	NT	NT	NT	NT	NT	NT	NT	NA	NT	NT	NT	NT	NT	NT	NT	NT	NT	NT	NT	NT	NT	NT	NT	NT	NT	NT	NT	NT	NT	NT
434	Xindongnin F	NT	NT	NT	NT	NT	NT	NT	NT	NT	NT	NT	NT	NT	NT	NT	NT	NT	NT	NT	NT	NT	7.3	NT	NT	NT	NT	NT	NT	NT	NT	NT	NT	NT	NT	NT	NT	NT	NT	NT	NT	NT	NT	NT	NT
435	Xindongnin G	NT	NT	NT	NT	NT	NT	NT	NT	NT	NT	NT	NT	NT	NT	NT	NT	NT	NT	NT	NT	NT	NA	NT	NT	NT	NT	NT	NT	NT	NT	NT	NT	NT	NT	NT	NT	NT	NT	NT	NT	NT	NT	NT	NT
436	Xindongnin A	NT	NT	NT	NT	NT	NT	NT	NT	NT	NT	NT	NT	NT	NT	NT	NT	NT	NT	NT	NT	NT	0.9	NT	NT	NT	NT	NT	NT	NT	NT	NT	NT	NT	NT	NT	NT	NT	NT	NT	NT	NT	NT	NT	NT
437	Xindongnin B	NT	NT	NT	NT	NT	NT	NT	NT	NT	NT	NT	NT	NT	NT	NT	NT	NT	NT	NT	NT	NT	1.2	NT	NT	NT	NT	NT	NT	NT	NT	NT	NT	NT	NT	NT	NT	NT	NT	NT	NT	NT	NT	NT	NT
438	Memissoidesin G	NT	NT	NT	NT	NT	NT	NT	NT	NT	NT	NT	NT	NT	NT	NT	NT	NT	NT	NT	NT	NT	0.3	NT	NT	NT	NT	NT	NT	NT	NT	NT	NT	NT	NT	NT	NT	NT	NT	NT	NT	NT	NT	NT	NT
439	Dawoensin A	NT	NT	NT	NT	NT	NT	NT	NT	NT	NT	NT	NT	NT	NT	NT	NT	NT	NT	NT	NT	NT	2.O	NT	NT	NT	NT	NT	NT	NT	NT	NT	NT	NT	NT	NT	NT	NT	NT	NT	NT	NT	NT	NT	NT
440	Glabcensin V	NT	NT	NT	NT	NT	NT	NT	NT	NT	NT	NT	NT	NT	NT	NT	NT	NT	NT	NT	NT	NT	0.4	NT	NT	NT	NT	NT	NT	NT	NT	NT	NT	NT	NT	NT	NT	NT	NT	NT	NT	NT	NT	NT	NT
441	Isorosthornin A	>50	>50	>50	>50	>50	NT	NT	NT	NT	NT	NT	NT	NT	NT	NT	NT	NT	NT	NT	NT	NT	NT	NT	NT	NT	NT	NT	NT	NT	NT	NT	NT	NT	NT	NT	NT	NT	NT	NT	NT	NT	NT	NT	NT
442	Isorosthornin B	>50	>50	>50	>50	>50	NT	NT	NT	NT	NT	NT	NT	NT	NT	NT	NT	NT	NT	NT	NT	NT	NT	NT	NT	NT	NT	NT	NT	NT	NT	NT	NT	NT	NT	NT	NT	NT	NT	NT	NT	NT	NT	NT	NT
443	Isorosthornin C	>50	>50	>50	>50	>50	NT	NT	NT	NT	NT	NT	NT	NT	NT	NT	NT	NT	NT	NT	NT	NT	NT	NT	NT	NT	NT	NT	NT	NT	NT	NT	NT	NT	NT	NT	NT	NT	NT	NT	NT	NT	NT	NT	NT
444	dihydroponicidin	>50	>50	>50	>50	>50	NT	NT	NT	NT	NT	NT	NT	NT	NT	NT	NT	NT	NT	NT	NT	NT	NT	NT	NT	NT	NT	NT	NT	NT	NT	NT	NT	NT	NT	NT	NT	NT	NT	NT	NT	NT	NT	NT	NT
445	Ponicidin	12.5	14.8	17.1	14.4	7.3	NT	NT	NT	NT	NT	NT	NT	NT	NT	NT	NT	NT	NT	NT	NT	NT	NT	NT	NT	NT	NT	NT	NT	NT	NT	NT	NT	NT	NT	NT	NT	NT	NT	NT	NT	NT	NT	NT	NT
446	Macrocalin B	14.7	35.5	>40		28.5	9.4	NT	NT	NT	NT	NT	NT	NT	NT	NT	NT	NT	NT	NT	NT	NT	NT	NT	NT	NT	NT	NT	NT	NT	NT	NT	NT	NT	NT	NT	NT	NT	NT	NT	NT	NT	NT	NT	NT
447	Xerophilusin B	1.37	2.37	3.95	2.43	2.13	NT	NT	NT	NT	NT	NT	NT	NT	NT	NT	NT	NT	NT	NT	NT	NT	NT	NT	NT	NT	NT	NT	NT	NT	NT	NT	NT	NT	NT	NT	NT	NT	NT	NT	NT	NT	NT	NT	NT
448	Rabdonervosin A	>50	>50	>50	>50	>50	NT	NT	NT	NT	NT	NT	NT	NT	NT	NT	NT	NT	NT	NT	NT	NT	NT	NT	NT	NT	NT	NT	NT	NT	NT	NT	NT	NT	NT	NT	NT	NT	NT	NT	NT	NT	NT	NT	NT
449	Rabdonervosin B	>50	>50	>50	>50	>50	NT	NT	NT	NT	NT	NT	NT	NT	NT	NT	NT	NT	NT	NT	NT	NT	NT	NT	NT	NT	NT	NT	NT	NT	NT	NT	NT	NT	NT	NT	NT	NT	NT	NT	NT	NT	NT	NT	NT
450	Parvifolines X	NT	NT	NT	NT	NT	NT	NT	NT	NT	NT	NT	NT	NT	NT	NT	NT	NT	NT	NT	NT	NT	>100	NT	NT	NT	NT	NT	NT	NT	NT	NT	NT	NT	NT	NT	NT	NT	NT	NT	NT	NT	NT	NT	NT
451	Parvifolines Y	NT	NT	NT	NT	NT	NT	NT	NT	NT	NT	NT	NT	NT	NT	NT	NT	NT	NT	NT	NT	NT	5.81	NT	NT	NT	NT	NT	NT	NT	NT	NT	NT	NT	NT	NT	NT	NT	NT	NT	NT	NT	NT	NT	NT
452	lasiodonin	NT	NT	NT	NT	NT	NT	NT	NT	NT	NT	NT	NT	NT	NT	NT	NT	NT	NT	NT	NT	NT	NT	NT	NT	NT	NT	NT	NT	NT	NT	NT	NT	NT	NT	NT	NT	NT	NT	NT	NT	NT	NT	NT	NT
453	Minheryins A	NT	NT	NT	NT	NT	NT	NT	NT	NT	NT	NT	NT	NT	NT	NT	NT	NT	NT	NT	NT	NT	NT	NT	NT	NT	NT	NT	NT	NT	NT	NT	NT	NT	NT	NT	NT	NT	NT	NT	NT	NT	NT	NT	NT
454	Minheryins B	NT	NT	NT	NT	NT	NT	NT	NT	NT	NT	NT	NT	NT	NT	NT	NT	NT	NT	NT	NT	NT	NT	NT	NT	NT	NT	NT	NT	NT	NT	NT	NT	NT	NT	NT	NT	NT	NT	NT	NT	NT	NT	NT	NT
455	Minheryins C	NT	NT	NT	NT	NT	NT	NT	NT	NT	NT	NT	NT	NT	NT	NT	NT	NT	NT	NT	NT	NT	NT	NT	NT	NT	NT	NT	NT	NT	NT	NT	NT	NT	NT	NT	NT	NT	NT	NT	NT	NT	NT	NT	NT
456	Minheryins D	NT	NT	NT	NT	NT	NT	NT	NT	NT	NT	NT	NT	NT	NT	NT	NT	NT	NT	NT	NT	NT	NT	NT	NT	NT	NT	NT	NT	NT	NT	NT	NT	NT	NT	NT	NT	NT	NT	NT	NT	NT	NT	NT	NT
457	Minheryins E	NT	NT	NT	NT	NT	NT	NT	NT	NT	NT	NT	NT	NT	NT	NT	NT	NT	NT	NT	NT	NT	NT	NT	NT	NT	NT	NT	NT	NT	NT	NT	NT	NT	NT	NT	NT	NT	NT	NT	NT	NT	NT	NT	NT
458	Minheryins F	NT	NT	NT	NT	NT	NT	NT	NT	NT	NT	NT	NT	NT	NT	NT	NT	NT	NT	NT	NT	NT	NT	NT	NT	NT	NT	NT	NT	NT	NT	NT	NT	NT	NT	NT	NT	NT	NT	NT	NT	NT	NT	NT	NT
459	Minheryins G	NT	NT	NT	NT	NT	NT	NT	NT	NT	NT	NT	NT	NT	NT	NT	NT	NT	NT	NT	NT	NT	NT	NT	NT	NT	NT	NT	NT	NT	NT	NT	NT	NT	NT	NT	NT	NT	NT	NT	NT	NT	NT	NT	NT
460	leukamenin F	NT	NT	NT	NT	NT	NT	NT	NT	NT	NT	NT	NT	NT	NT	NT	NT	NT	NT	NT	NT	NT	NT	NT	NT	NT	NT	NT	NT	NT	NT	NT	NT	NT	NT	NT	NT	NT	NT	NT	NT	NT	NT	NT	NT
461	Excisoidesin	NT	NT	NT	NT	NT	NT	NT	NT	NT	NT	NT	NT	NT	NT	NT	NT	NT	NT	NT	NT	NT	NT	NT	NT	NT	NT	NT	NT	NT	NT	NT	NT	NT	NT	NT	NT	NT	NT	NT	NT	NT	NT	NT	NT
462	Leukamenin E	NT	NT	NT	NT	NT	NT	NT	NT	NT	NT	NT	NT	NT	NT	NT	NT	NT	NT	NT	NT	NT	NT	NT	NT	NT	NT	NT	NT	NT	NT	NT	NT	NT	NT	NT	NT	NT	NT	NT	NT	NT	NT	NT	NT
463	Wangzaozin A	NT	NT	NT	NT	NT	NT	NT	NT	NT	NT	NT	NT	NT	NT	NT	NT	NT	NT	NT	NT	NT	NT	NT	NT	NT	NT	NT	NT	NT	NT	NT	NT	NT	NT	NT	NT	NT	NT	NT	NT	NT	NT	NT	NT
464	Pseurata A	NT	NT	NT	NT	NT	NT	NT	NT	NT	NT	NT	NT	NT	NT	NT	NT	NT	NT	NT	NT	NT	NT	NT	NT	NT	NT	NT	NT	NT	NT	NT	NT	NT	NT	NT	NT	NT	NT	NT	NT	NT	NT	NT	NT
465	Racemosin A	NT	NT	NT	NT	NT	NT	NT	NT	NT	NT	NT	NT	NT	NT	NT	NT	NT	NT	NT	NT	NT	NT	NT	NT	NT	NT	NT	NT	NT	NT	NT	NT	NT	NT	NT	NT	NT	NT	NT	NT	NT	NT	NT	NT
466	Isojaponin A	NT	NT	NT	NT	NT	NT	NT	NT	NT	NT	NT	NT	NT	NT	NT	NT	NT	NT	NT	NT	NT	NT	NT	NT	NT	NT	NT	NT	NT	NT	NT	NT	NT	NT	NT	NT	NT	NT	NT	NT	NT	NT	NT	NT
467	Isojaponin B	NT	NT	NT	NT	NT	NT	NT	NT	NT	NT	NT	NT	NT	NT	NT	NT	NT	NT	NT	NT	NT	NT	NT	NT	NT	NT	NT	NT	NT	NT	NT	NT	NT	NT	NT	NT	NT	NT	NT	NT	NT	NT	NT	NT
468	Rabdosinate	NT	NT	NT	NT	NT	NT	NT	NT	NT	NT	NT	NT	NT	NT	NT	NT	NT	NT	NT	NT	NT	NT	NT	NT	NT	NT	NT	NT	NT	NT	NT	NT	NT	NT	NT	NT	NT	NT	NT	NT	NT	NT	NT	NT
469	Maoyecrystal D	NT	NT	NT	NT	NT	NT	NT	NT	NT	NT	NT	NT	NT	NT	NT	NT	NT	NT	NT	NT	NT	NT	NT	NT	NT	NT	NT	NT	NT	NT	NT	NT	NT	NT	NT	NT	NT	NT	NT	NT	NT	NT	NT	NT
470	Rabdosin A	NT	NT	NT	NT	NT	NT	NT	NT	NT	NT	NT	NT	NT	NT	NT	NT	NT	NT	NT	NT	NT	NT	NT	NT	NT	NT	NT	NT	NT	NT	NT	NT	NT	NT	NT	NT	NT	NT	NT	NT	NT	NT	NT	NT
471	Isodonoiol	NT	NT	NT	NT	NT	NT	NT	NT	NT	NT	NT	NT	NT	NT	NT	NT	NT	NT	NT	NT	NT	NT	NT	NT	NT	NT	NT	NT	NT	NT	NT	NT	NT	NT	NT	NT	NT	NT	NT	NT	NT	NT	NT	NT
472	Epinodosin	NT	NT	NT	NT	NT	NT	NT	NT	NT	NT	NT	NT	NT	NT	NT	NT	NT	NT	NT	NT	NT	NT	NT	NT	NT	NT	NT	NT	NT	NT	NT	NT	NT	NT	NT	NT	NT	NT	NT	NT	NT	NT	NT	NT
473	Rabdoternin H	NT	NT	NT	NT	NT	NT	NT	NT	NT	NT	NT	NT	NT	NT	NT	NT	NT	NT	NT	NT	NT	NT	NT	NT	NT	NT	NT	NT	NT	NT	NT	NT	NT	NT	NT	NT	NT	NT	NT	NT	NT	NT	NT	NT
474	Rabdosin B	NT	NT	NT	NT	NT	NT	NT	NT	NT	NT	NT	NT	NT	NT	NT	NT	NT	NT	NT	NT	NT	NT	NT	NT	NT	NT	NT	NT	NT	NT	NT	NT	NT	NT	NT	NT	NT	NT	NT	NT	NT	NT	NT	NT
475	Acetylexidonin	NT	NT	NT	NT	NT	NT	NT	NT	NT	NT	NT	NT	NT	NT	NT	NT	NT	NT	NT	NT	NT	NT	NT	NT	NT	NT	NT	NT	NT	NT	NT	NT	NT	NT	NT	NT	NT	NT	NT	NT	NT	NT	NT	NT
476	Lasiokaurin	NT	NT	NT	NT	NT	NT	NT	NT	NT	NT	NT	NT	NT	NT	NT	NT	NT	NT	NT	NT	NT	NT	NT	NT	NT	NT	NT	NT	NT	NT	NT	NT	NT	NT	NT	NT	NT	NT	NT	NT	NT	NT	NT	NT
477	Lasiodonin	NT	NT	NT	NT	NT	NT	NT	NT	NT	NT	NT	NT	NT	NT	NT	NT	NT	NT	NT	NT	NT	NT	NT	NT	NT	NT	NT	NT	NT	NT	NT	NT	NT	NT	NT	NT	NT	NT	NT	NT	NT	NT	NT	NT
478	Oridonin	NT	NT	NT	NT	NT	NT	NT	NT	NT	NT	NT	NT	NT	NT	NT	NT	NT	NT	NT	NT	NT	NT	NT	NT	NT	NT	NT	NT	NT	NT	NT	NT	NT	NT	NT	NT	NT	NT	NT	NT	NT	NT	NT	NT
479	Effusanin A	NT	NT	NT	NT	NT	NT	NT	NT	NT	NT	NT	NT	NT	NT	NT	NT	NT	NT	NT	NT	NT	NT	NT	NT	NT	NT	NT	NT	NT	NT	NT	NT	NT	NT	NT	NT	NT	NT	NT	NT	NT	NT	NT	NT
480	Shikokianin	NT	NT	NT	NT	NT	NT	NT	NT	NT	NT	NT	NT	NT	NT	NT	NT	NT	NT	NT	NT	NT	NT	NT	NT	NT	NT	NT	NT	NT	NT	NT	NT	NT	NT	NT	NT	NT	NT	NT	NT	NT	NT	NT	NT
481	Rosthorin A	NT	NT	NT	NT	NT	NT	NT	NT	NT	NT	NT	NT	NT	NT	NT	NT	NT	NT	NT	NT	NT	NT	NT	NT	NT	NT	NT	NT	NT	NT	NT	NT	NT	NT	NT	NT	NT	NT	NT	NT	NT	NT	NT	NT
482	Isodonal	NT	NT	NT	NT	NT	NT	NT	NT	NT	NT	NT	NT	NT	NT	NT	NT	NT	NT	NT	NT	NT	NT	NT	NT	NT	NT	NT	NT	NT	NT	NT	NT	NT	NT	NT	NT	NT	NT	NT	NT	NT	NT	NT	NT
483	Lasionkaurinol	NT	NT	NT	NT	NT	NT	NT	NT	NT	NT	NT	NT	NT	NT	NT	NT	NT	NT	NT	NT	NT	NT	NT	NT	NT	NT	NT	NT	NT	NT	NT	NT	NT	NT	NT	NT	NT	NT	NT	NT	NT	NT	NT	NT
484	Enmenol	NT	NT	NT	NT	NT	NT	NT	NT	NT	NT	NT	NT	NT	NT	NT	NT	NT	NT	NT	NT	NT	NT	NT	NT	NT	NT	NT	NT	NT	NT	NT	NT	NT	NT	NT	NT	NT	NT	NT	NT	NT	NT	NT	NT
485	Lushanrubescensin F	NT	NT	NT	NT	NT	NT	NT	NT	NT	NT	NT	NT	NT	NT	NT	NT	NT	NT	NT	NT	NT	NT	NT	NT	NT	NT	NT	NT	NT	NT	NT	NT	NT	NT	NT	NT	NT	NT	NT	NT	NT	NT	NT	NT
486	Jianshirubesin G	NT	NT	NT	NT	NT	NT	NT	NT	NT	NT	NT	NT	NT	NT	NT	NT	NT	NT	NT	NT	NT	NT	NT	NT	NT	NT	NT	NT	NT	NT	NT	NT	NT	NT	NT	NT	NT	NT	NT	NT	NT	NT	NT	NT
487	Jianshirubesin H	NT	NT	NT	NT	NT	NT	NT	NT	NT	NT	NT	NT	NT	NT	NT	NT	NT	NT	NT	NT	NT	NT	NT	NT	NT	NT	NT	NT	NT	NT	NT	NT	NT	NT	NT	NT	NT	NT	NT	NT	NT	NT	NT	NT
488	Jianshirubesin I	NT	NT	NT	NT	NT	NT	NT	NT	NT	NT	NT	NT	NT	NT	NT	NT	NT	NT	NT	NT	NT	NT	NT	NT	NT	NT	NT	NT	NT	NT	NT	NT	NT	NT	NT	NT	NT	NT	NT	NT	NT	NT	NT	NT
489	Ponicidin	3.56	4.22	10.92	13.29	6.88	NT	NT	NT	NT	NT	NT	NT	NT	NT	NT	NT	NT	NT	NT	NT	NT	NT	NT	NT	NT	NT	NT	NT	NT	NT	NT	NT	NT	NT	NT	NT	NT	NT	NT	NT	NT	NT	NT	NT
490	Parvifoline AA	10.2	10.2	17.31	17.61	24.11	NT	NT	NT	NT	NT	NT	NT	NT	NT	NT	NT	NT	NT	NT	NT	NT	NT	NT	NT	NT	NT	NT	NT	NT	NT	NT	NT	NT	NT	NT	NT	NT	NT	NT	NT	NT	NT	NT	NT
491	Laxiflorin D	NT	NT	NT	NT	NT	NT	NT	NT	NT	NT	NT	NT	NT	NT	NT	NT	NT	NT	NT	NT	NT	NT	NT	NT	NT	NT	NT	NT	NT	NT	NT	NT	NT	NT	NT	NT	NT	NT	NT	NT	NT	NT	NT	NT
492	Laxiflorin E	NT	NT	NT	NT	NT	NT	NT	NT	NT	NT	NT	NT	NT	NT	NT	NT	NT	NT	NT	NT	NT	NT	NT	NT	NT	NT	NT	NT	NT	NT	NT	NT	NT	NT	NT	NT	NT	NT	NT	NT	NT	NT	NT	NT
493	laxiflorin C	NT	NT	NT	NT	NT	NT	NT	NT	NT	NT	NT	NT	NT	NT	NT	NT	NT	NT	NT	NT	NT	NT	NT	NT	NT	NT	NT	NT	NT	NT	NT	NT	NT	NT	NT	NT	NT	NT	NT	NT	NT	NT	NT	NT
494	Rabdoternin A	NT	NT	NT	NT	NT	NT	NT	NT	NT	NT	NT	NT	NT	NT	NT	NT	NT	NT	NT	NT	NT	NT	NT	NT	NT	NT	NT	NT	NT	NT	NT	NT	NT	NT	NT	NT	NT	NT	NT	NT	NT	NT	NT	NT
495	Rabdoternin B	NT	NT	NT	NT	NT	NT	NT	NT	NT	NT	NT	NT	NT	NT	NT	NT	NT	NT	NT	NT	NT	NT	NT	NT	NT	NT	NT	NT	NT	NT	NT	NT	NT	NT	NT	NT	NT	NT	NT	NT	NT	NT	NT	NT
496	Isoscoparin D	NT	NT	>10	>10	NT	NT	NT	NT	NT	NT	NT	NT	>10	NT	NT	NT	NT	NT	NT	NT	NT	NT	>10	NT	NT	NT	NT	NT	NT	NT	NT	NT	NT	NT	NT	NT	NT	NT	NT	NT	NT	NT	NT	NT
497	Isoscoparin E	NT	NT	NT	NT	NT	NT	NT	NT	NT	NT	NT	NT	NT	NT	NT	NT	NT	NT	NT	NT	NT	NT	NT	NT	NT	NT	NT	NT	NT	NT	NT	NT	NT	NT	NT	NT	NT	NT	NT	NT	NT	NT	NT	NT
498	Isoscoparin F	NT	NT	9.9	3.9	NT	NT	NT	NT	NT	NT	NT	NT	>10	NT	NT	NT	NT	NT	NT	NT	NT	NT	9.2	NT	NT	NT	NT	NT	NT	NT	NT	NT	NT	NT	NT	NT	NT	NT	NT	NT	NT	NT	NT	NT
499	Isoscoparin G	NT	NT	NT	NT	NT	NT	NT	NT	NT	NT	NT	NT	NT	NT	NT	NT	NT	NT	NT	NT	NT	NT	NT	NT	NT	NT	NT	NT	NT	NT	NT	NT	NT	NT	NT	NT	NT	NT	NT	NT	NT	NT	NT	NT
500	Sculponins D	NT	NT	NT	NT	NT	NT	NT	1.12	NT	NT	1	NT	NT	NT	NT	NT	NT	NT	NT	NT	NT	1.5	NT	NT	NT	NT	NT	NT	NT	NT	NT	NT	NT	NT	NT	NT	NT	NT	NT	NT	NT	NT	NT	NT

501	Sculponins E	NT	NT	NT	NT	NT	NT	NT	1.62	NT	NT	1.85	NT	NT	NT	NT	NT	NT	NT	NT	NT	NT	2	NT	NT	NT	NT	NT	NT	NT	NT	NT	NT	NT	NT	NT	NT	NT	NT	NT	NT	NT	NT	NT	NT	NT
502	3β,6β,11α-trihydroxy-15R-acetoxy-6,20-epoxy-6,7-seco-ent-kaur16-en-1β,7-olid	NT	NT	NT	NT	NT	NT	NT	NT	NT	NT	NT	NT	NT	NT	NT	NT	NT	NT	NT	NT	NT	NT	NT	NT	NT	NT	NT	NT	NT	NT	NT	NT	NT	NT	NT	NT	NT	NT	NT	NT	NT	NT	NT	NT
503	Epinodosin	NT	NT	NT	NT	NT	NT	NT	1.62	NT	NT	2.1	NT	NT	NT	NT	NT	NT	NT	NT	NT	NT	0.56	NT	NT	NT	NT	NT	NT	NT	NT	NT	NT	NT	NT	NT	NT	NT	NT	NT	NT	NT	NT	NT	NT
504	Maoyecrystal D	NT	NT	NT	NT	NT	NT	NT	NT	NT	NT	NT	NT	NT	NT	NT	NT	NT	NT	NT	NT	NT	NT	NT	NT	NT	NT	NT	NT	NT	NT	NT	NT	NT	NT	NT	NT	NT	NT	NT	NT	NT	NT	NT	NT
505	Nodosin	NT	NT	NT	NT	NT	NT	NT	1.92	NT	NT	1.9	NT	NT	NT	NT	NT	NT	NT	NT	NT	NT	0.54	NT	NT	NT	NT	NT	NT	NT	NT	NT	NT	NT	NT	NT	NT	NT	NT	NT	NT	NT	NT	NT	NT
506	Enmein	NT	NT	NT	NT	NT	NT	NT	2.02	NT	NT	>10	NT	NT	NT	NT	NT	NT	NT	NT	NT	NT	1.62	NT	NT	NT	NT	NT	NT	NT	NT	NT	NT	NT	NT	NT	NT	NT	NT	NT	NT	NT	NT	NT	NT
507	6β,7β,11β,15β,19-pentahydroxy-7α,20-epoxy-entkaur-16-ene	NT	NT	NT	NT	NT	NT	NT	NT	NT	NT	NT	NT	NT	NT	NT	NT	NT	NT	NT	NT	NT	NT	NT	NT	NT	NT	NT	NT	NT	NT	NT	NT	NT	NT	NT	NT	NT	NT	NT	NT	NT	NT	NT	NT
508	6β,7β,15β,19-tetrahydroxy-7α,20-epoxy-*ent*-kaur-1,16-dien11-one	NT	NT	NT	NT	NT	NT	NT	NT	NT	NT	NT	NT	NT	NT	NT	NT	NT	NT	NT	NT	NT	NT	NT	NT	NT	NT	NT	NT	NT	NT	NT	NT	NT	NT	NT	NT	NT	NT	NT	NT	NT	NT	NT	NT
509	Enmenol	NT	NT	NT	NT	NT	NT	NT	NT	NT	NT	NT	NT	NT	NT	NT	NT	NT	NT	NT	NT	NT	NT	NT	NT	NT	NT	NT	NT	NT	NT	NT	NT	NT	NT	NT	NT	NT	NT	NT	NT	NT	NT	NT	NT
510	11β,19β-dihydroxy-6,19;6,20-diepoxy-6,7-seco-*ent*-kaur-16-en-15-one-1β,7-olide	NT	NT	NT	NT	NT	NT	NT	NT	NT	NT	NT	NT	NT	NT	NT	NT	NT	NT	NT	NT	NT	NT	NT	NT	NT	NT	NT	NT	NT	NT	NT	NT	NT	NT	NT	NT	NT	NT	NT	NT	NT	NT	NT	NT
511	11β,19α-dihydroxy-6,19;6,20- diepoxy-6,7-seco-*ent*-kaur-16-en-15-one-1β- 7-olid	NT	NT	NT	NT	NT	NT	NT	NT	NT	NT	NT	NT	NT	NT	NT	NT	NT	NT	NT	NT	NT	NT	NT	NT	NT	NT	NT	NT	NT	NT	NT	NT	NT	NT	NT	NT	NT	NT	NT	NT	NT	NT	NT	NT
512	11β,15α,19β-trihydroxy-6,19;6,20-diepoxy-6,7-seco-*ent*-kaur16-en-1β,7-olide	NT	NT	NT	NT	NT	NT	NT	NT	NT	NT	NT	NT	NT	NT	NT	NT	NT	NT	NT	NT	NT	NT	NT	NT	NT	NT	NT	NT	NT	NT	NT	NT	NT	NT	NT	NT	NT	NT	NT	NT	NT	NT	NT	NT
513	11β,15α,19α-trihydroxy-6,19;6,20-diepoxy6,7-seco-*ent*-kaur-16-en-1β,7-olide	NT	NT	NT	NT	NT	NT	NT	NT	NT	NT	NT	NT	NT	NT	NT	NT	NT	NT	NT	NT	NT	NT	NT	NT	NT	NT	NT	NT	NT	NT	NT	NT	NT	NT	NT	NT	NT	NT	NT	NT	NT	NT	NT	NT
514	Sculponeatin A	NT	NT	NT	NT	NT	NT	NT	1.77	NT	NT	>10	NT	NT	NT	NT	NT	NT	NT	NT	NT	NT	1.62	NT	NT	NT	NT	NT	NT	NT	NT	NT	NT	NT	NT	NT	NT	NT	NT	NT	NT	NT	NT	NT	NT
515	Sculponeatin B	NT	NT	NT	NT	NT	NT	NT	NT	NT	NT	NT	NT	NT	NT	NT	NT	NT	NT	NT	NT	NT	NT	NT	NT	NT	NT	NT	NT	NT	NT	NT	NT	NT	NT	NT	NT	NT	NT	NT	NT	NT	NT	NT	NT
516	6β,7β,15β,19-tetrahydroxy-7α,20-epoxy-*ent*-kaur-1,16-dien11-one	NT	NT	NT	NT	NT	NT	NT	NT	NT	NT	NT	NT	NT	NT	NT	NT	NT	NT	NT	NT	NT	NT	NT	NT	NT	NT	NT	NT	NT	NT	NT	NT	NT	NT	NT	NT	NT	NT	NT	NT	NT	NT	NT	NT
517	Ememogin	NT	NT	NT	NT	NT	NT	NT	NT	NT	NT	NT	NT	NT	NT	NT	NT	NT	NT	NT	NT	NT	NT	NT	NT	NT	NT	NT	NT	NT	NT	NT	NT	NT	NT	NT	NT	NT	NT	NT	NT	NT	NT	NT	NT
518	Sculponeatin C	NT	NT	NT	NT	NT	NT	NT	0.68	NT	NT	2.73	NT	NT	NT	NT	NT	NT	NT	NT	NT	NT	0.78	NT	NT	NT	NT	NT	NT	NT	NT	NT	NT	NT	NT	NT	NT	NT	NT	NT	NT	NT	NT	NT	NT
519	Sculponeatin D	NT	NT	NT	NT	NT	NT	NT	NT	NT	NT	NT	NT	NT	NT	NT	NT	NT	NT	NT	NT	NT	NT	NT	NT	NT	NT	NT	NT	NT	NT	NT	NT	NT	NT	NT	NT	NT	NT	NT	NT	NT	NT	NT	NT
520	1β,7β,13α-trihydroxy-6β,15β-diacetoxy-7α,20-epoxy-ent-kaur-16-ene	NT	NT	NT	NT	NT	NT	NT	NT	NT	NT	NT	NT	NT	NT	NT	NT	NT	NT	NT	NT	NT	NT	NT	NT	NT	NT	NT	NT	NT	NT	NT	NT	NT	NT	NT	NT	NT	NT	NT	NT	NT	NT	NT	NT
521	Thichokaurin	NT	NT	NT	NT	NT	NT	NT	NT	NT	NT	NT	NT	NT	NT	NT	NT	NT	NT	NT	NT	NT	NT	NT	NT	NT	NT	NT	NT	NT	NT	NT	NT	NT	NT	NT	NT	NT	NT	NT	NT	NT	NT	NT	NT
522	Sylvestrisides C	NT	NT	NT	NT	NT	NT	NT	NT	NT	NT	NT	NT	NT	NT	NT	NT	NT	NT	NT	NT	NT	NT	NT	NT	NT	NT	NT	NT	NT	NT	NT	NT	NT	NT	NT	NT	NT	NT	NT	NT	NT	NT	NT	NT
523	Sylvestrisides D	NT	NT	NT	NT	NT	NT	NT	NT	NT	NT	NT	NT	NT	NT	NT	NT	NT	NT	NT	NT	NT	NT	NT	NT	NT	NT	NT	NT	NT	NT	NT	NT	NT	NT	NT	NT	NT	NT	NT	NT	NT	NT	NT	NT
524	Sylvestrisides E	NT	NT	NT	NT	NT	NT	NT	NT	NT	NT	NT	NT	NT	NT	NT	NT	NT	NT	NT	NT	NT	NT	NT	NT	NT	NT	NT	NT	NT	NT	NT	NT	NT	NT	NT	NT	NT	NT	NT	NT	NT	NT	NT	NT
525	*Ent*-3α,7,15,16-tetrahydroxypimar-8(14)-ene	NT	NT	NT	NT	NT	NT	NT	NT	NT	NT	NT	NT	NT	NT	NT	NT	NT	NT	NT	NT	NT	NT	NT	NT	NT	NT	NT	NT	NT	NT	NT	NT	NT	NT	NT	NT	NT	NT	NT	NT	NT	NT	NT	NT
526	*Ent*-16- acetoxy-3α,15-dihydroxy-7-hydroperoxypimar-8(14)-en-3R-*O*-glucopyr	NT	NT	NT	NT	NT	NT	NT	NT	NT	NT	NT	NT	NT	NT	NT	NT	NT	NT	NT	NT	NT	NT	NT	NT	NT	NT	NT	NT	NT	NT	NT	NT	NT	NT	NT	NT	NT	NT	NT	NT	NT	NT	NT	NT
527	*Ent*-2,15,16-trihydroxypimar-8(14)-en-19-oic acid	NT	NT	NT	NT	NT	NT	NT	NT	NT	NT	NT	NT	NT	NT	NT	NT	NT	NT	NT	NT	NT	NT	NT	NT	NT	NT	NT	NT	NT	NT	NT	NT	NT	NT	NT	NT	NT	NT	NT	NT	NT	NT	NT	NT
528	*Ent*-2-oxo-15,16,19-trihydroxypimar-8(14)-ene	NT	NT	NT	NT	NT	NT	NT	NT	NT	NT	NT	NT	NT	NT	NT	NT	NT	NT	NT	NT	NT	NT	NT	NT	NT	NT	NT	NT	NT	NT	NT	NT	NT	NT	NT	NT	NT	NT	NT	NT	NT	NT	NT	NT
529	Kirenol	NT	NT	NT	NT	NT	NT	NT	NT	NT	NT	NT	NT	NT	NT	NT	NT	NT	NT	NT	NT	NT	NT	NT	NT	NT	NT	NT	NT	NT	NT	NT	NT	NT	NT	NT	NT	NT	NT	NT	NT	NT	NT	NT	NT
530	Darutigenol	NT	NT	NT	NT	NT	NT	NT	NT	NT	NT	NT	NT	NT	NT	NT	NT	NT	NT	NT	NT	NT	NT	NT	NT	NT	NT	NT	NT	NT	NT	NT	NT	NT	NT	NT	NT	NT	NT	NT	NT	NT	NT	NT	NT
531	*Ent*-3α,15,16,19-tetrahydroxypimar-8(14)- ene	NT	NT	NT	NT	NT	NT	NT	NT	NT	NT	NT	NT	NT	NT	NT	NT	NT	NT	NT	NT	NT	NT	NT	NT	NT	NT	NT	NT	NT	NT	NT	NT	NT	NT	NT	NT	NT	NT	NT	NT	NT	NT	NT	NT
532	Darutoside	NT	NT	NT	NT	NT	NT	NT	NT	NT	NT	NT	NT	NT	NT	NT	NT	NT	NT	NT	NT	NT	NT	NT	NT	NT	NT	NT	NT	NT	NT	NT	NT	NT	NT	NT	NT	NT	NT	NT	NT	NT	NT	NT	NT
533	*Ent*-16-acetoxy-15-hydroxypimar-8(14)- en-3α-*O*-glucopyranoside	NT	NT	NT	NT	NT	NT	NT	NT	NT	NT	NT	NT	NT	NT	NT	NT	NT	NT	NT	NT	NT	NT	NT	NT	NT	NT	NT	NT	NT	NT	NT	NT	NT	NT	NT	NT	NT	NT	NT	NT	NT	NT	NT	NT
534	Isopropylidenkirenol	NT	NT	NT	NT	NT	NT	NT	NT	NT	NT	NT	NT	NT	NT	NT	NT	NT	NT	NT	NT	NT	NT	NT	NT	NT	NT	NT	NT	NT	NT	NT	NT	NT	NT	NT	NT	NT	NT	NT	NT	NT	NT	NT	NT
535	*Ent*-3α,15,16-trihydroxypimar-8(14)-en-15,16-acetonide	NT	NT	NT	NT	NT	NT	NT	NT	NT	NT	NT	NT	NT	NT	NT	NT	NT	NT	NT	NT	NT	NT	NT	NT	NT	NT	NT	NT	NT	NT	NT	NT	NT	NT	NT	NT	NT	NT	NT	NT	NT	NT	NT	NT
536	*Ent*-3α,15,16-trihydroxypimar-8(14)-en-3α-*O*-glucopyranoside-15,16-acetonide	NT	NT	NT	NT	NT	NT	NT	NT	NT	NT	NT	NT	NT	NT	NT	NT	NT	NT	NT	NT	NT	NT	NT	NT	NT	NT	NT	NT	NT	NT	NT	NT	NT	NT	NT	NT	NT	NT	NT	NT	NT	NT	NT	NT
537	*Ent*-18-acetoxy-17-hydroxy16H-kauran-19-oic acid	NT	NT	NT	NT	NT	NT	NT	NT	NT	NT	NT	NT	NT	NT	NT	NT	NT	NT	NT	NT	NT	NT	NT	NT	NT	NT	NT	NT	NT	NT	NT	NT	NT	NT	NT	NT	NT	NT	NT	NT	NT	NT	NT	NT
538	*Ent*-18-acetoxy-17-isobutyryloxy-16- kauran-19- oic acid	NT	NT	NT	NT	NT	NT	NT	NT	NT	NT	NT	NT	NT	NT	NT	NT	NT	NT	NT	NT	NT	NT	NT	NT	NT	NT	NT	NT	NT	NT	NT	NT	NT	NT	NT	NT	NT	NT	NT	NT	NT	NT	NT	NT
539	*Ent*-18-acetoxy-16α,17-dihydroxykauran-19-oic acid	NT	NT	NT	NT	NT	NT	NT	NT	NT	NT	NT	NT	NT	NT	NT	NT	NT	NT	NT	NT	NT	NT	NT	NT	NT	NT	NT	NT	NT	NT	NT	NT	NT	NT	NT	NT	NT	NT	NT	NT	NT	NT	NT	NT
540	*Ent*-18-acetoxy-16α-hydroxy-17-isobutyryloxykauran-19-oic acid	NT	NT	NT	NT	NT	NT	NT	NT	NT	NT	NT	NT	NT	NT	NT	NT	NT	NT	NT	NT	NT	NT	NT	NT	NT	NT	NT	NT	NT	NT	NT	NT	NT	NT	NT	NT	NT	NT	NT	NT	NT	NT	NT	NT
541	*Ent-* 16α,17,18-trihydroxykauran-19-oic acid	NT	NT	NT	NT	NT	NT	NT	NT	NT	NT	NT	NT	NT	NT	NT	NT	NT	NT	NT	NT	NT	NT	NT	NT	NT	NT	NT	NT	NT	NT	NT	NT	NT	NT	NT	NT	NT	NT	NT	NT	NT	NT	NT	NT
542	*Ent*-17,18-dihydroxy-16-kauran-19-oicacid	NT	NT	NT	NT	NT	NT	NT	NT	NT	NT	NT	NT	NT	NT	NT	NT	NT	NT	NT	NT	NT	NT	NT	NT	NT	NT	NT	NT	NT	NT	NT	NT	NT	NT	NT	NT	NT	NT	NT	NT	NT	NT	NT	NT
543	*Ent*-17-isobutyryloxy-18-hydroxykauran-19-oic acid	NT	NT	NT	NT	NT	NT	NT	NT	NT	NT	NT	NT	NT	NT	NT	NT	NT	NT	NT	NT	NT	NT	NT	NT	NT	NT	NT	NT	NT	NT	NT	NT	NT	NT	NT	NT	NT	NT	NT	NT	NT	NT	NT	NT
544	*Ent*-16α,17-dihydroxykauran-19-oic acid	NT	NT	NT	NT	NT	NT	NT	NT	NT	NT	NT	NT	NT	NT	NT	NT	NT	NT	NT	NT	NT	NT	NT	NT	NT	NT	NT	NT	NT	NT	NT	NT	NT	NT	NT	NT	NT	NT	NT	NT	NT	NT	NT	NT
545	*Ent*-17-hydroxy-16α-kauran-19-oic acid	NT	NT	NT	NT	NT	NT	NT	NT	NT	NT	NT	NT	NT	NT	NT	NT	NT	NT	NT	NT	NT	NT	NT	NT	NT	NT	NT	NT	NT	NT	NT	NT	NT	NT	NT	NT	NT	NT	NT	NT	NT	NT	NT	NT
546	*Ent*-19-methyl-17- hydroxy-16α-kauran-19-oic acid	NT	NT	NT	NT	NT	NT	NT	NT	NT	NT	NT	NT	NT	NT	NT	NT	NT	NT	NT	NT	NT	NT	NT	NT	NT	NT	NT	NT	NT	NT	NT	NT	NT	NT	NT	NT	NT	NT	NT	NT	NT	NT	NT	NT
547	*Ent*-16α-kauran-17,19- dioic acid	NT	NT	NT	NT	NT	NT	NT	NT	NT	NT	NT	NT	NT	NT	NT	NT	NT	NT	NT	NT	NT	NT	NT	NT	NT	NT	NT	NT	NT	NT	NT	NT	NT	NT	NT	NT	NT	NT	NT	NT	NT	NT	NT	NT
548	16α,17-acetonide of ent-16α,17-dihydroxykauran-19-oic acid	NT	NT	NT	NT	NT	NT	NT	NT	NT	NT	NT	NT	NT	NT	NT	NT	NT	NT	NT	NT	NT	NT	NT	NT	NT	NT	NT	NT	NT	NT	NT	NT	NT	NT	NT	NT	NT	NT	NT	NT	NT	NT	NT	NT
549	16α,17-acetonide of *ent*-19- methyl-16α,17-dihydroxykauran-19-oic acid	NT	NT	NT	NT	NT	NT	NT	NT	NT	NT	NT	NT	NT	NT	NT	NT	NT	NT	NT	NT	NT	NT	NT	NT	NT	NT	NT	NT	NT	NT	NT	NT	NT	NT	NT	NT	NT	NT	NT	NT	NT	NT	NT	NT
550	Sagittine H	NT	NT	NT	NT	NT	NT	NT	NT	NT	NT	NT	NT	NT	NT	NT	NT	NT	NT	NT	NT	NT	NT	NT	NT	NT	NT	NT	NT	NT	NT	NT	NT	NT	NT	NT	NT	NT	NT	NT	NT	NT	NT	NT	NT
551	Sclareol	NT	NT	NT	NT	NT	NT	NT	NT	NT	NT	NT	NT	NT	NT	NT	NT	NT	NT	NT	NT	NT	NT	NT	NT	NT	NT	NT	NT	NT	NT	NT	NT	NT	NT	NT	NT	NT	NT	NT	NT	NT	NT	NT	NT
552	19-*β*-*L*-3'-acetoxyarabinofuranosyl- ent-kaur-16-ene-19-oate	NT	NT	NT	NT	NT	NT	NT	NT	NT	NT	NT	NT	NT	NT	NT	NT	NT	NT	NT	NT	NT	NT	NT	NT	NT	NT	NT	NT	NT	NT	NT	NT	NT	NT	NT	NT	NT	NT	NT	NT	NT	NT	NT	NT
553	5α-hydroxy-ent-rosa-15-en-19-oic acid	NT	NT	NT	NT	NT	NT	NT	NT	NT	NT	NT	NT	NT	NT	NT	NT	NT	NT	NT	NT	NT	NT	NT	NT	NT	NT	NT	NT	NT	NT	NT	NT	NT	NT	NT	NT	NT	NT	NT	NT	NT	NT	NT	NT
554	*Ent*-13-*epi*-manoyl oxide-18-ol	NT	NT	NT	NT	NT	NT	NT	NT	NT	NT	NT	NT	NT	NT	NT	NT	NT	NT	NT	NT	NT	NT	NT	NT	NT	NT	NT	NT	NT	NT	NT	NT	NT	NT	NT	NT	NT	NT	NT	NT	NT	NT	NT	NT
555	*Ent*-13-*epi*-manoyl oxide-18-oic acid	NT	NT	NT	NT	NT	NT	NT	NT	NT	NT	NT	NT	NT	NT	NT	NT	NT	NT	NT	NT	NT	NT	NT	NT	NT	NT	NT	NT	NT	NT	NT	NT	NT	NT	NT	NT	NT	NT	NT	NT	NT	NT	NT	NT
556	*Ent*-kauranol	NT	NT	NT	NT	NT	NT	NT	NT	NT	NT	NT	NT	NT	NT	NT	NT	NT	NT	NT	NT	NT	NT	NT	NT	NT	NT	NT	NT	NT	NT	NT	NT	NT	NT	NT	NT	NT	NT	NT	NT	NT	NT	NT	NT
557	19-O-*α*-*L*-arabinofuranosyl-*ent*-rosa-5,15-diene	NT	NT	NT	NT	NT	NT	NT	NT	NT	NT	NT	NT	NT	NT	NT	NT	NT	NT	NT	NT	NT	NT	NT	NT	NT	NT	NT	NT	NT	NT	NT	NT	NT	NT	NT	NT	NT	NT	NT	NT	NT	NT	NT	NT
558	Lasiodin	NT	NT	NT	NT	NT	NT	NT	NT	NT	NT	NT	NT	NT	NT	NT	NT	NT	NT	NT	NT	NT	NT	NT	NT	NT	NT	NT	NT	NT	NT	NT	NT	NT	NT	NT	NT	NT	NT	NT	NT	NT	NT	NT	NT
559	Rabdophyllin H	NT	NT	NT	NT	NT	NT	NT	NT	NT	NT	NT	NT	NT	NT	NT	NT	NT	NT	NT	NT	NT	NT	NT	NT	NT	NT	NT	NT	NT	NT	NT	NT	NT	NT	NT	NT	NT	NT	NT	NT	NT	NT	NT	NT
560	Xerophilusins L	NT	NT	NT	NT	NT	NT	NT	NT	NT	NT	NT	NT	NT	NT	NT	NT	NT	NT	NT	NT	NT	NT	NT	NT	NT	NT	NT	NT	NT	NT	NT	NT	NT	NT	NT	NT	NT	NT	NT	NT	NT	NT	NT	NT
561	Xerophilusins M	NT	NT	NT	NT	NT	NT	NT	NT	NT	NT	NT	NT	NT	NT	NT	NT	NT	NT	NT	NT	NT	NT	NT	NT	NT	NT	NT	NT	NT	NT	NT	NT	NT	NT	NT	NT	NT	NT	NT	NT	NT	NT	NT	NT
562	Xerophilusins N	NT	NT	NT	NT	NT	NT	NT	NT	NT	NT	NT	NT	NT	NT	NT	NT	NT	NT	NT	NT	NT	NT	NT	NT	NT	NT	NT	NT	NT	NT	NT	NT	NT	NT	NT	NT	NT	NT	NT	NT	NT	NT	NT	NT
563	Robdoternin A	NT	NT	NT	NT	NT	NT	NT	NT	NT	NT	NT	NT	NT	NT	NT	NT	NT	NT	NT	NT	NT	NT	NT	NT	NT	NT	NT	NT	NT	NT	NT	NT	NT	NT	NT	NT	NT	NT	NT	NT	NT	NT	NT	NT
564	Ponicidin	NT	NT	NT	NT	NT	NT	NT	NT	NT	NT	NT	NT	NT	NT	NT	NT	NT	NT	NT	NT	0.32	0.09	NT	NT	NT	NT	NT	NT	NT	NT	NT	NT	NT	NT	NT	NT	NT	NT	NT	NT	NT	NT	NT	NT
565	Dayecrystal A	NT	NT	NT	NT	NT	NT	NT	NT	NT	NT	NT	NT	NT	NT	NT	NT	NT	NT	NT	NT	NT	NT	NT	NT	NT	NT	NT	NT	NT	NT	NT	NT	NT	NT	NT	NT	NT	NT	NT	NT	NT	NT	NT	NT
566	Dayecrystal B	NT	NT	NT	NT	NT	NT	NT	NT	NT	NT	NT	NT	NT	NT	NT	NT	NT	NT	NT	NT	NT	NT	NT	NT	NT	NT	NT	NT	NT	NT	NT	NT	NT	NT	NT	NT	NT	NT	NT	NT	NT	NT	NT	NT
567	Epinodosin	NT	NT	NT	NT	NT	NT	NT	NT	NT	NT	NT	NT	NT	NT	NT	NT	NT	NT	NT	NT	NT	NT	NT	NT	NT	NT	NT	NT	NT	NT	NT	NT	NT	NT	NT	NT	NT	NT	NT	NT	NT	NT	NT	NT
568	Isodonoiol	NT	NT	NT	NT	NT	NT	NT	NT	NT	NT	NT	NT	NT	NT	NT	NT	NT	NT	NT	NT	NT	NT	NT	NT	NT	NT	NT	NT	NT	NT	NT	NT	NT	NT	NT	NT	NT	NT	NT	NT	NT	NT	NT	NT
569	lasiodonin	NT	NT	NT	NT	NT	NT	NT	NT	NT	NT	NT	NT	NT	NT	NT	NT	NT	NT	NT	NT	NT	NT	NT	NT	NT	NT	NT	NT	NT	NT	NT	NT	NT	NT	NT	NT	NT	NT	NT	NT	NT	NT	NT	NT
570	Nervosanin B	NT	NT	NT	NT	NT	NT	NT	NT	NT	NT	NT	NT	NT	NT	NT	NT	NT	NT	NT	NT	NT	NT	NT	NT	NT	NT	NT	NT	NT	NT	NT	NT	NT	NT	NT	NT	NT	NT	NT	NT	NT	NT	NT	NT
